# Bioengineered Exosome‐Loaded Immunomodulatory Bioadhesive Spray Reverses Liver Fibrosis and Metabolic Dysfunction That Triggers Beneficial Gut‐Liver Crosstalk in Chronic Fatty Liver Disease

**DOI:** 10.1002/advs.76420

**Published:** 2026-07-14

**Authors:** Triya Saha, Ayushi Mairal, Shreya Mehrotra, Shiv K. Sarin, Ashok Kumar

**Affiliations:** ^1^ Department of Biological Sciences and Bioengineering Indian Institute of Technology Kanpur Kanpur UP India; ^2^ Centre For Environmental Science and Engineering Indian Institute of Technology Kanpur Kanpur UP India; ^3^ Department of Hepatology Institute of Liver and Biliary Sciences New Delhi India; ^4^ The Mehta Family Centre for Engineering in Medicine Indian Institute of Technology Kanpur Kanpur UP India; ^5^ Centre For Nanosciences Indian Institute of Technology Kanpur Kanpur UP India; ^6^ Centre of Excellence for Materials in Medicine Gangwal School of Medical Sciences and Technology Indian Institute of Technology Kanpur Kanpur UP India

**Keywords:** bioadhesive spray, exosomes, fibrosis, gut‐liver axis, immunomodulation, MAFLD

## Abstract

Metabolic dysfunction‐associated fatty liver disease (MAFLD) is a progressive liver disorder with complex pathogenesis and no approved treatments beyond lifestyle changes. Here, we report a sprayable and biodegradable bioadhesive hydrogel loaded with exosomes derived from human mesenchymal stromal cells that provides sustained therapy localized to the liver. In a rat model of chronic MAFLD, a single application of the spray markedly improved liver pathology and systemic metabolic indices over the effects of lifestyle interventions. Specifically, collagen deposition, liver‐enzyme levels, insulin resistance, and chronic inflammation were all attenuated. Furthermore, the spray led to an increase in markers of gut‐barrier integrity and gut bacterial diversity and modulated key pathways of lipid metabolism, inflammation, and fibrosis, as we show via multi‐omics profiling of liver tissue. Our findings strongly suggest that exosomes delivered to the liver via a sprayable anti‐inflammatory hydrogel can address the multifactorial pathology of MAFLD, yielding improvements in liver histology and function, and inducing systemic metabolic homeostasis.

## Graphical abstract

Umbilical cord stem cell‐derived exosomes encapsulated in an immunomodulatory bioadhesive hydrogel spray enable localized therapy, sustained vesicle delivery, macrophage polarization, suppress systemic inflammation, and mitigate glucose and insulin homeostasis impairments in MAFLD.

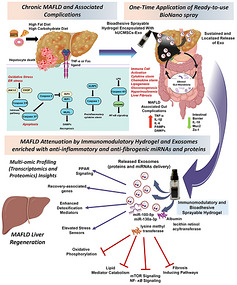

## 1 Introduction

The global surge in obesity and type 2 diabetes has driven a parallel rise in metabolic dysfunction‐associated fatty liver disease (MAFLD, formerly NAFLD) [1]. Affecting nearly one in four adults worldwide, MAFLD represents the most common chronic liver disorder [2, 3]. This multifactorial metabolic disease manifests through hepatic lipid accumulation, insulin resistance, hypertension, and persistent liver enzyme alterations, often accompanied by gut barrier disruption and microbiota imbalance [4]. Without timely intervention, MAFLD advances to metabolic dysfunction‐associated steatohepatitis (MASH), ultimately progressing to cirrhosis, liver failure, and hepatocellular carcinoma (HCC) [5].

Lifestyle modifications (diet and exercise) are the main recommendation, but are often insufficient, especially in advanced MAFLD associated with liver fibrosis. Medications like pioglitazone, metformin, resmetirom, vitamin E, and other drugs have shown limited efficacy [6, 7, 8, 9]. The lack of standardized treatment and the complex pathology of MAFLD pose major clinical challenges, underscoring the need for therapies that both halt progression and support liver regeneration. Regenerative approaches such as mesenchymal stromal cell (MSC) therapy have shown promise in attenuating liver injury conditions, largely attributed to the paracrine effects of MSCs. In particular, MSC‐derived extracellular vesicles (or exosomes) are recognized as a major mechanism of therapeutic action by MSCs. Exosomes carry bioactive cargo (miRNAs, proteins, lipids, and DNA) that can reduce inflammation and promote tissue repair depending upon their origin, which makes them attractive as a cell‐free therapy approach [10, 11, 12].

However, the clinical translation of exosomes faces several challenges, including rapid clearance from circulation (short half‐life), potential off‐target effects, and difficulty in achieving sustained localized concentrations [13, 14]. To overcome these barriers, engineering exosome‐loaded advanced biomaterial platforms for sustained and targeted release represents a promising strategy to enhance therapeutic outcomes and broaden the clinical applicability of exosome‐based interventions. For instance, human umbilical cord mesenchymal stem cells (hUCMSCs)‐derived exosomes integrated with hyaluronic acid hydrogels have shown efficacy in promoting endometrial repair in rat models [15]. Similarly, exosome‐functionalized nanocomposites have been explored for enhancing wound healing by promoting angiogenesis and depositing collagen in diabetic animal models [16]. Nevertheless, many such studies rely on freshly isolated exosomes, posing logistical hurdles for clinical translation, and are surgically invasive. Exosomes typically require fresh isolation before use, which complicates their availability in a ready‐to‐use format. Additional concerns include their limited stability under ambient conditions, suboptimal storage parameters, and challenges in achieving precise targeting.

To address these issues, our previous work showed that a bioprinted hepatic patch delivering hUCMSCs‐exosomes could alleviate fatty liver disease in a rat model [17]. However, the development of the implantable patch was perhaps not amenable to minimally invasive delivery, making its translation difficult. Building on those insights, to have enhanced therapeutic efficacy, we designed a clinically translatable off‐the‐shelf bioadhesive sprayable hydrogel (termed BioNano spray) that will provide sustained release of exosomes locally, in addition to the immunomodulatory hydrogel used as a carrier. The spray is a composite hydrogel formulated from methacrylated versions of gelatin (GelMA) and hyaluronic acid (MeHA), conjugated with dopamine to confer bioadhesive properties via in situ polymerization to polydopamine. It is applied as a liquid that is rapidly cross‐linked on the liver surface under brief light exposure (405 nm), forming a thin adherent gel. This gel encapsulating the exosomes enables their sustained release (∼95% over 4 weeks) directly on the liver. We hypothesized that this strategy would overcome the limitations associated with systemic exosome delivery by enabling stable bioadhesion to the liver surface while simultaneously providing an immunomodulatory matrix through hyaluronic acid and polydopamine, thereby offering synergistic therapeutic effects in addition to exosome‐mediated activity.

In this study, we show that a single minimally invasive application of the BioNano spray yields statistically significant improvements in liver inflammation, fibrosis, and metabolic function, outperforming conventional lifestyle interventions. It is the first demonstration of a minimally invasive localized biomaterial‐based exosome therapy for MAFLD, serving as a new therapeutic paradigm for liver fibrosis. We observed that while only polymer spray (without exosomes) alone demonstrated significant therapeutic benefits in an experimental chronic MAFLD model, the combination of immunomodulatory spray and exosomes exhibited superior outcomes. This synergistic effect resulted in reduced hepatic and systemic inflammation, improved liver, adipose tissue, and pancreatic functioning, in addition to restoring gut barrier integrity and gut microbiota diversity, thereby enhancing the potential of multimodal therapeutic approaches for effective liver fibrosis and chronic fatty liver treatment. Both in vitro and in vivo experiments demonstrated the therapy's efficacy in promoting cell survival, mediating macrophage polarization, reducing inflammation, and facilitating hepatoprotection. Our mechanistic analyses (multi‐omic profiling) suggest that the therapy induces a shift toward anti‐inflammatory immune profiles and restores homeostasis in the associated gut‐liver, liver‐adipose tissue, and liver‐pancreas axis, mainly driven by the exosomal proteins, miRNAs, and use of intrinsic immunomodulatory material. Furthermore, from a clinical translation perspective, the sprayable hydrogel was evaluated for its stability following lyophilization and was found to maintain both physical integrity and functional activity for at least six months. The key advances lie in the long‐term stability, highlighting its potential as an off‐the‐shelf therapeutic platform, offering practical advantages in terms of storage, transportation, and large‐scale clinical use. Additionally, this platform is minimally invasive, immunomodulatory, and anti‐inflammatory, which addresses key translational hurdles for exosome therapies.

## 2 Results and Discussion

### 2.1 Characterization of Isolated Exosomes and Its Proteomic Profiling

**FIGURE 1:**
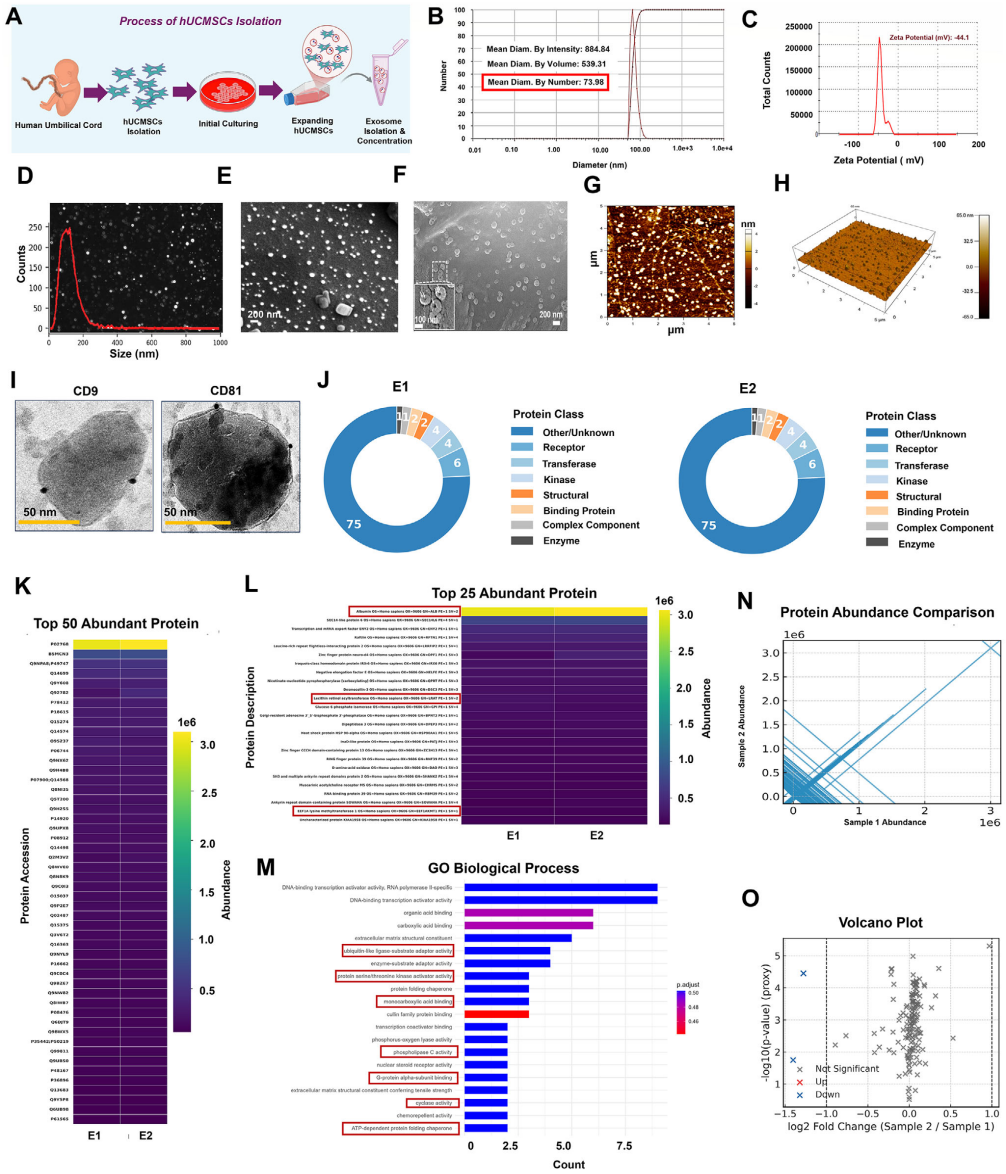
Isolation and characterization of hUCMSCs‐Exo. (A) Schematic illustration of the process of hUCMSCs‐Exo isolation; (B) Dynamic Light Scattering (DLS) analysis of exosomes; (C) Zeta potential assessment of isolated exosomes; (D) Nanoparticle tracking analysis (NTA) of the exosomes; (E) Scanning Electron Microscopy (SEM) of the exosomes; (F) Field Emission Scanning Electron Microscopy of the isolated exosomes (FE‐SEM); (G) 2D topographical image by Atomic Force Microscopy (AFM); (H) 3D image by AFM; (I) Immunogold Transmission Electron Microscopy (i‐TEM) of the exosomes for CD9 and CD81 surface marker protein; (J) Proteomic profiling to determine protein content and class by ESI LC‐MS in technical duplicates of E1 and E2; (K) Heatmap chart of top 50 abundant proteins; (L) Heatmap representation of top 25 abundant proteins; (M) Gene ontology (GO) analysis of identified proteins to determine the biological process the exosomes are involved with; (N) Protein abundance comparison between the technical duplicates of E1 and E2; (O) Volcano plot representing the fold change of abundance between E1 and E2.

Human umbilical cord mesenchymal stem cell‐derived exosomes (hUCMSCs‐Exo) carry a diverse repertoire of bioactive molecules with the potential to modulate gene expression and cellular pathways for exerting therapeutic effects. In this study, exosomes were isolated from the conditioned medium of cultured hUCMSCs as depicted in the schematic overview (Figure 1A). Dynamic light scattering (DLS) confirmed a hydrodynamic diameter of ∼75 nm (Figure 1B), while zeta potential measurements indicated a negative surface charge of −44 ± 5.44 mV (Figure 1C), suggesting colloidal stability. Nanoparticle tracking analysis (NTA) revealed that the exosomes exhibited a predominant size distribution within the range of 80–100 nm, with an average mode peak centered at 86.7 nm. The particle concentration was estimated at 2.37 ± 1.03 × 10^11^ particles/mL, corresponding to 100 µg of total protein (Figure 1D and Figure S2A). Size and morphology were further determined by a series of microscopy techniques, where SEM and FE‐SEM revealed the size of exosomes to fall in the range of 100–120 nm with spherical morphology in SEM; however, in FE‐SEM, the characteristic cup‐shaped morphology with lipid membrane was observed (Figure 1E,F). AFM analysis (2D and 3D visualization) of topography also indicated a similar size range and dome‐shaped 3D morphology of isolated exosomes (Figure 1G,H), with force mapping revealing the Young's and reduced modulus of 1.65 ± 0.54 MPa and 1.85 ± 0.61 MPa, respectively (Figure S2F,G). Morphological assessment via immunogold‐labeled transmission electron microscopy (Immuno‐TEM) showed the characteristic spherical vesicular morphology of exosomes, with an average diameter of 121.90 ± 26.5 nm. Additionally, surface expression of canonical exosomal markers CD9 and CD81 further confirmed their identity (Figure 1I). Together, these results validate the successful isolation of high‐purity, stable hUCMSCs‐Exo.

Subsequent proteomic profiling was conducted to elucidate their molecular cargo and gain mechanistic insights into their therapeutic potential, complementing the miRNA landscape of our previous report [17]. Protein isolation and identification by ESI LC‐MS in technical duplicates (E1 and E2) revealed the presence of 153 proteins (Figure 1L and Figures S1 and S2). The main protein classes identified were receptor, transferase, kinase, structural, binding protein, complex component, and enzyme (Figure 1J). Highly abundant proteins identified were albumin, lecithin retinol acyltransferase, and lysine methyl transferase, which have tremendous beneficial roles toward liver regeneration and especially play a positive role toward promoting hepatocyte growth and proliferation for regeneration and positively influence the regain of homeostasis of lipid, glucose, and insulin metabolism [18, 19] (Figure 1K,L). Gene ontology (GO) analysis revealed enrichment of proteins involved in carboxylic acid binding, ubiquitin‐like ligase adaptor activity, phospholipase C signaling, kinase activation, and ATP‐dependent chaperone function (Figure 1M–O and Figure S2). The detection of HSP90 and HSP70 further validated the presence of characteristic exosomal surface markers (Figure S2). Our prior study also investigated the presence of the canonical marker CD9 by immunoblot, which was absent in the corresponding cell lysate control [17].

MAFLD, being a systemic metabolic disease, requires effective therapy targeting the interconnected regulatory networks involved. Therefore, the use of advanced bioactive formulations as minimally invasive therapeutic platforms, combined with the synergistic effects of exosomes enriched with diverse miRNA and protein cargos to modulate multi‐organ pathological pathways, remains underexplored.

### 2.2 Development of Bioadhesive Photocrosslinkable and Sprayable Nanotherapeutics (BioNano Spray) Formulations

**FIGURE 2:**
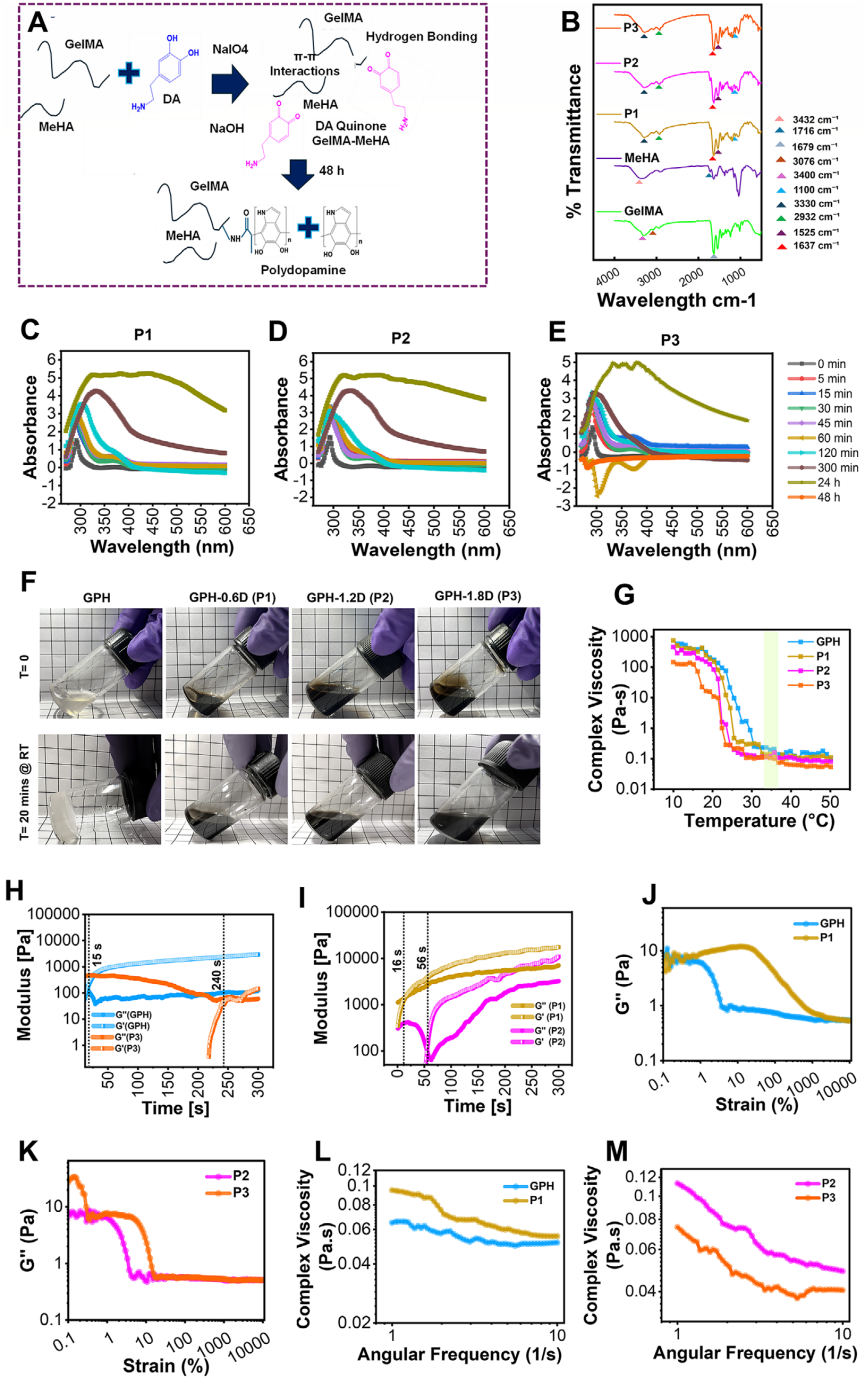
Synthesis of bioadhesive sprayable formulations and assessing their fluid properties. (A) Reaction scheme for synthesis of adhesive formulations; (B) FTIR Spectroscopy of the dopamine‐conjugated formulations and GelMA, MeHA; (C–E) UV–vis spectroscopy assessment for the in situ dopamine polymerization, *n* = 3; (F) Vial‐tilt assay of various formulations (modified and unmodified) after 20 min at RT; (G) Temperature sweep analysis of all the formulations; (H, I) Photorheological assessment of the different formulations; (J, K) Amplitude sweep analysis; (L, M) Frequency sweep analysis of the developed sprayable formulations.

To develop bioadhesive sprayable formulations amenable to minimally invasive administration, we incorporated dopamine into a methacrylated gelatin (GelMA)/ methacrylated hyaluronic acid (MeHA)/polyethylene glycol diacrylate (PEGDA) polymer blend. This facilitated the conjugation of dopamine and the subsequent in situ polymerization and oxidation of dopamine to form polydopamine, produced via the intermediate DA quinone‐GelMA/MeHA (Figure 2A). For the synthesis, the polymer blend concentration was kept constant with GelMA at 5% w/v, MeHA at 1% w/v, and PEGDA at 1% v/v. Different concentrations of dopamine hydrochloride (0.6% w/v for P1, 1.2% w/v for P2, and 1.8% w/v for P3) were added in the presence of strong alkali sodium hydroxide (NaOH) and the oxidizing agent, sodium periodate (NaIO_4_) to initiate polymerization and facilitate conjugation through *π–π*‐ stacking and hydrogen bonding (Figure 2A). All components of the spray are clinically relevant; hyaluronic acid and gelatin are used in FDA‐approved products, and polydopamine is used in treatments, ensuring its translational relevance. Successful modification was confirmed by FTIR spectroscopy, which displayed characteristic peaks for all formulations (P1, P2, and P3) at 3330, 2932, 1637, 1525, and 1100 cm^−1^, corresponding to O‐H and N‐H stretching (hydroxyl and amine groups), C‐H stretching, C═C and C═O stretching (indicating the presence of aromatic rings, quinones, and amide I), N‐H bending, aromatic ring vibrations, and C‐O stretching (phenolic groups). Additionally, GelMA FTIR spectra exhibited peaks at 1679, 3076, and 3432 cm^−1^, corresponding to amide I, N‐H, and O‐H stretching vibrations, respectively. Similarly, MeHA displayed peaks at 1716 and 3700–3200 cm^−1^, corresponding to C═O and O‐H stretching, respectively (Figure 2B). UV–vis analysis of the conjugated polymer solutions (P1, P2, and P3) showed a broad shoulder spanning 250–600 nm, with the absorbance peak gradually shifting to higher wavelengths after 48 h, indicating complete oxidation of dopamine to polydopamine (Figure 2C–E). This wavelength shift results from the addition of benzene rings to the polymer blend, reducing the bandgap. Furthermore, the increasing absorbance at 280 nm after 120 min of reaction initiation signifies dopamine oxidation and polydopamine conjugation to the GelMA/MeHA blend (Figure S3A). Post‐modification, there was a significant reduction in viscosity compared to the unmodified sprayable formulation (GPH) (Figure 2F and Figure S3).

Additionally, the vial tilt assay illustrated physical gelation of GPH at room temperature (RT) post 20 min; however, the modified formulations were still in the sol phase (Figure 2F). The reduced physical gelation in the modified formulations (P1–P3) is primarily attributed to steric hindrance introduced by bulky polydopamine (PDA) moieties, which interfere with effective polymer chain interactions and limit intermolecular association between methacryloyl groups of GelMA and MeHA. This steric effect disrupts the formation of a physically entangled network at RT, thereby maintaining the system in a sol state prior to photo‐cross‐linking. Although PDA introduces additional non‐covalent interactions, these do not effectively contribute to network formation due to steric constraints and reduced chain mobility. Similar PDA‐induced steric interference affecting polymer/hydrogel network assembly has also been reported in previous studies [20, 21, 22]. The phase transition characteristics and flow behavior of the formulations were evaluated using dynamic rheological analysis. Temperature sweep measurements (10°C to 50°C) revealed a progressive decline in complex viscosity across GPH, P1, P2, and P3, indicating an inverse correlation between viscosity and the degree of polydopamine conjugation. At 37°C, all formulations displayed reduced complex viscosity, aligning with the requirements for sprayable delivery (Figure 2G). To assess the influence of polydopamine‐mediated photo‐cross‐linking, photo‐rheological tests were conducted, showing a gradual increase in covalent gelation upon 405 nm light exposure, with gelation times of ∼15, 16, 56, and 240 s for GPH, P1, P2, and P3, respectively (Figure 2H,I). Amplitude sweep analysis established the linear viscoelastic region (LVER) threshold at approximately 10% strain for all formulations, while frequency sweep analysis confirmed stability up to an angular frequency of 10 rad/s (Figure 2J–M). Subsequently, the sprayability of all formulations was evaluated at varying spray distances (Figure S3 and Video SV1). Collectively, these rheological assessments delineate the formulations’ temperature‐dependent viscosity, cross‐linking kinetics, and mechanical stability under oscillatory and frequency variations.

### 2.3 Assessment of the Bioadhesive Property of Developed Formulations

**FIGURE 3:**
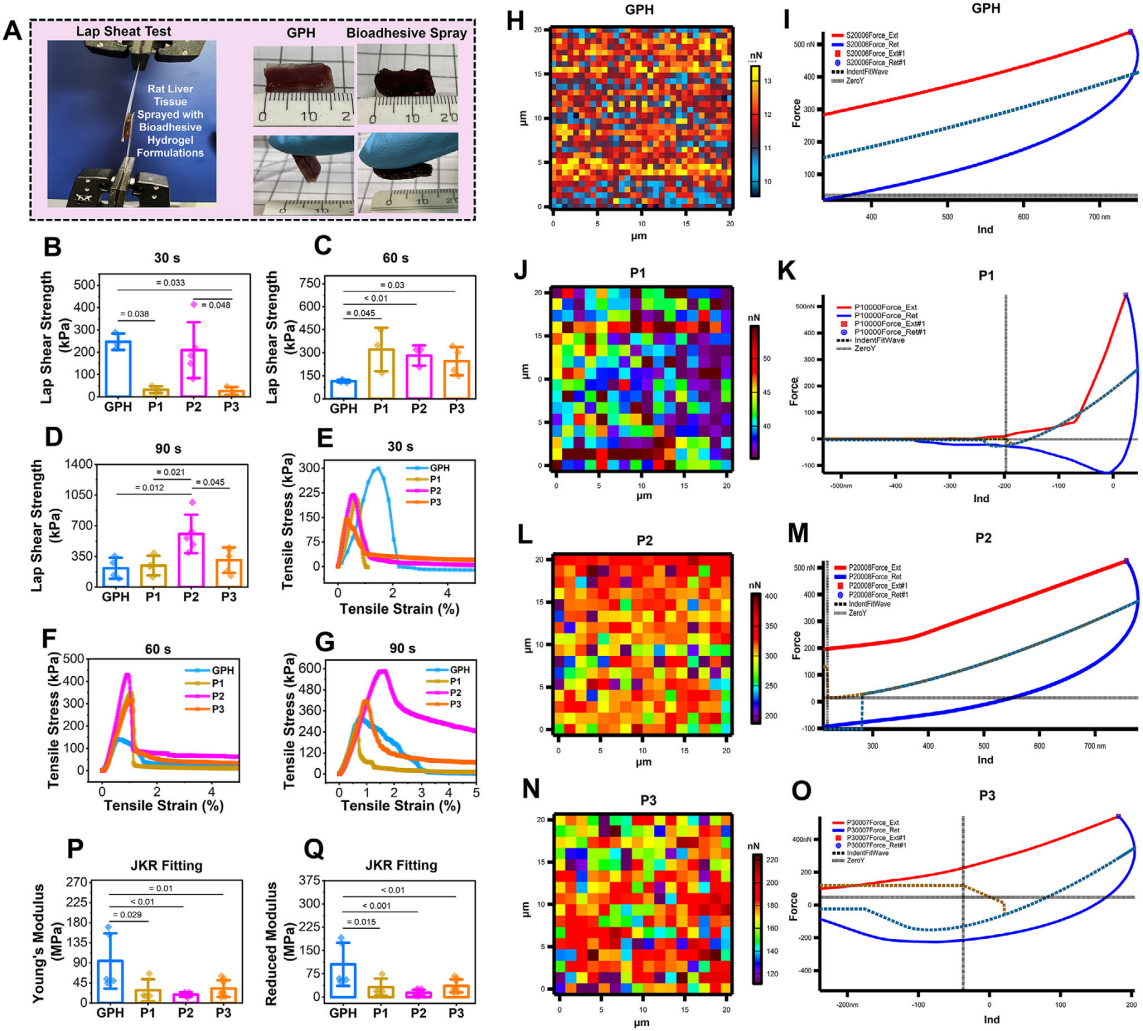
Evaluating the adhesive property of the unmodified and polydopamine‐conjugated formulations. (A) Digital images illustrating the conducted lap‐shear test and enhanced biadhesivity of modified sprayable hydrogels to the unmodified hydrogels; (B–D) Lap shear strength of the various formulations when cross‐linked for 30, 60, and 90s, respectively. Data is expressed as Mean ± S.D, n ≥ 4; (E–G) Stress–stress curves illustrating the stability and adhesivity of tissue‐adhered hydrogels when cross‐linked for 30, 60, and 90s, respectively; (H, I) Force mapping and force vs. indent graph for GPH (cross‐linked for 60s); (J, K) Force mapping and force vs. indent graph for P1(cross‐linked for 60s); (L–0) Force mapping and force vs. indent graph for P2 and P3 respectively; (P) Young's modulus (sample modulus) calculated from force mapping analysis using JKR curve fitting model; (Q) Reduced modulus calculated from force mapping analysis using JKR curve fitting model. Data is expressed as Mean ± S.D, n ≥ 3.

To investigate the bioadhesivity of polydopamine‐conjugated hydrogel formulations (GPH, P1, P2, and P3) and compare with the unmodified one (GPH), a series of experiments starting with a lap shear test was carried out. This test was designed to assess the adhesive strength of the sprayed hydrogel layer adhered after blue light illumination for three‐time durations of 30, 60, and 90 s onto the rat liver tissue (Figure 3A).

When normalized to bonded area, GPH exhibited the highest adhesive strength among groups cross‐linked for 30 s (251.1 ± 43.3 kPa), followed by P2, P1, and P3 (Figure 3B). At 60 s, the trend reversed, with P2 showing a markedly higher strength than GPH (Figure 3C). Prolonged cross‐linking (90 s) further enhanced adhesion in P2 (p = 0.012), surpassing both GPH and P3 (Figure 3D). Overall, this assessment indicated that when the cross‐linking time was 60 s, the P2 formulation displayed higher shear strength than the other modified and unmodified solutions. A gradual increase in covalent cross‐linking of GPH led to increased brittleness, resulting in lower strength compared to the modified ones. Additionally, stress–strain curves revealed a characteristic elastic region, followed by yielding and eventual failure. Among the hydrogels exposed for 30 s, GPH had the highest tensile stress, indicating greater elastic behavior and stability. For exposures of 60 and 90 s, P2 demonstrated a greater elastic nature (higher modulus, ∼435 and ∼586 kPa, respectively) before distortion, surpassing all other experimental groups at the same durations (Figure 3E–G). Burst pressure testing demonstrated superior wet adhesion of P2, effectively sealing injured rat colon tissue compared with GPH (Figure S4). Based on these assessments, considering both adhesivity and stability of the photocross‐linked soft hydrogels, the P2 formulation was selected with a 60 s exposure. The P2 formulation at 60 s cross‐linking time duration exhibited superior elastic and bioadhesive properties, as evidenced by both lap shear strength and stress–strain curves. Furthermore, affirmative conclusions were driven by force mapping analysis, where thin hydrogels of GPH, P1, P2, and P3 (60 s exposure duration) were assessed for the Young's modulus (YM) and reduced modulus (RM) using JKR fitting of Force vs. Indent curves. P2 and P3 exhibited broader retraction curves with greater negative retraction force (signifying greater adhesivity) compared to the other soft hydrogels (Figure 3H–O). However, when modulus was assessed, GPH showed significantly higher modulus (YM: ∼94 MPa, RM: ∼105 MPa) than the dopamine‐modified hydrogels, thereby signifying a softer modulus of modified hydrogel formulations (P2; YM: ∼18 MPa and RM: ∼14 MPa) (Figure 3P,Q). Polydopamine incorporation generated a heterogeneous and mechanically dynamic network, where increased chain mobility and PDA‐induced steric hindrance reduced the localized nanoscale stiffness measured by AFM, while catechol‐mediated intermolecular interactions, enhanced energy dissipation, and improved load transfer contributed to higher bulk tensile stiffness and macroscopic mechanical integrity of the hydrogels. Similar scale‐dependent differences between local AFM‐derived modulus and bulk mechanical properties have been reported previously for heterogeneous hydrogel systems and soft biomaterials [23, 24]. Overall, wet tissue adhesivity in the polydopamine‐conjugated formulation (P2) is attributed to the oxidative self‐polymerization of dopamine, wherein catechol groups are converted to quinones under mildly alkaline/oxidative conditions, followed by Michael addition and Schiff base reactions. This results in a heterogeneous, cross‐linked PDA network containing indole‐ and pyrrole‐like structures with catechol/quinone functionalities in multiple oxidation states. The generated quinones readily form covalent bonds with nucleophilic residues on tissue surfaces, enabling strong interfacial anchoring. In parallel, non‐covalent interactions, including *π–π* stacking with aromatic amino acids (tyrosine, phenylalanine, tryptophan) and hydrophobic interactions, reduce interfacial hydration and further enhance adhesion. Similar catechol‐mediated adhesive mechanisms have been extensively reported in mussel‐inspired bioadhesive systems, which show enhanced interfacial anchoring and adhesion under wet physiological environments [25, 26]. Collectively, these results highlight the elastic and adhesive behavior of the modified hydrogels, with P2 displaying the most favorable combination of softness, stability, and resilience after 60 s of covalent cross‐linking.

### 2.4 In vitro Assessment of the Bioadhesive Sprayable Formulations and Fabrication of an off‐the‐shelf BioNano Spray

**FIGURE 4:**
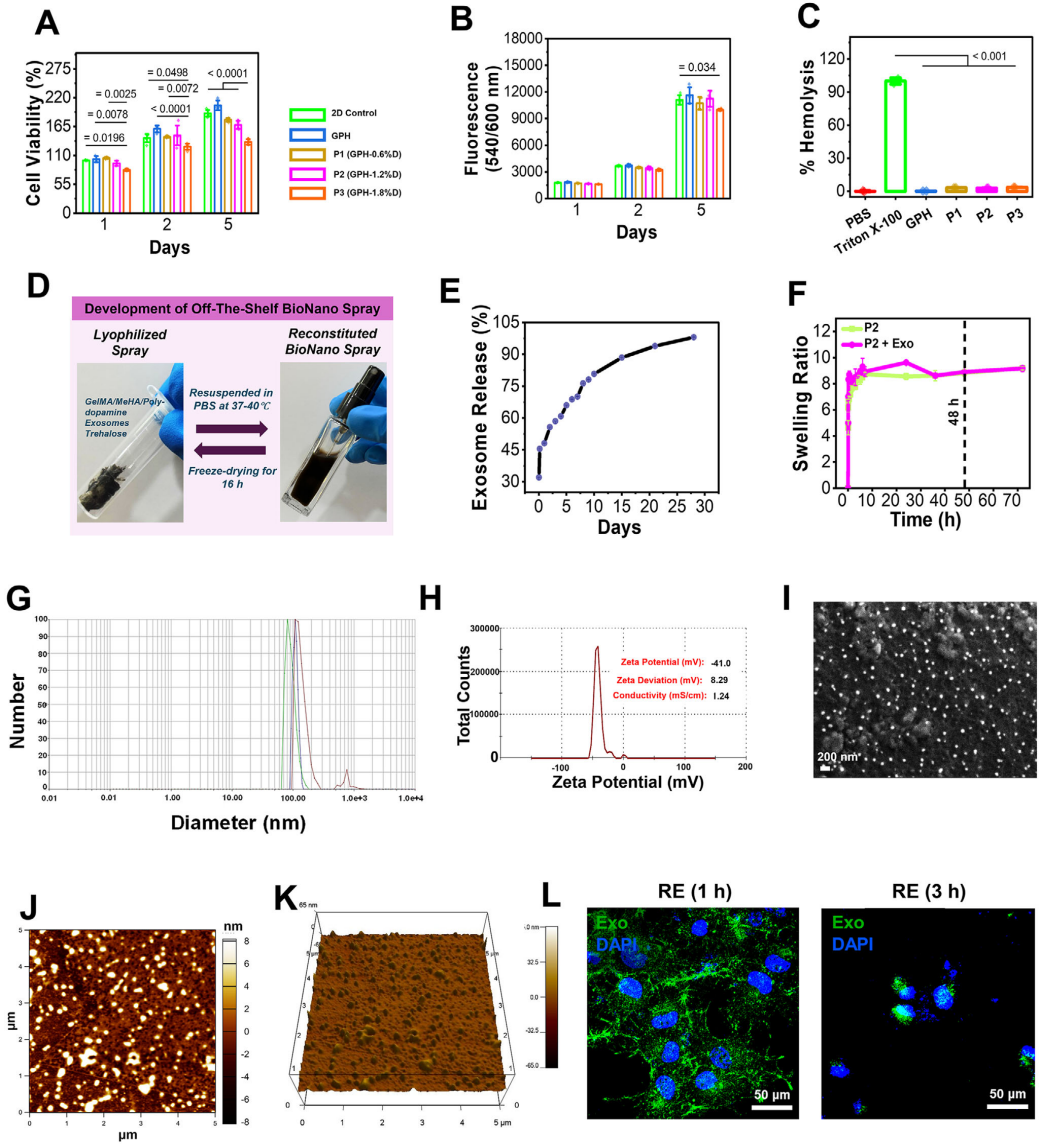
Cytocompatibility and stability of the off‐the‐shelf BioNano spray approach. (A, B) MTT assay and resazurin assay of IHH cells in the presence of various sprayable formulations. Data is expressed as Mean ± S.D, *n* = 4; (C) Hemolysis assay to assess the compatibility of the different sprayable solutions. Data is expressed as Mean ± S.D, n = 2;  (D) Digital images and schematic representation for the synthesis of BioNano spray and fabrication of its off‐the‐shelf form; (E) Exosome release from P2 hydrogels over 28 days; (F) Swelling ability assessment of P2 and P2 encapsulated with exosomes over 72 h; (G) DLS analysis to assess the hydrodynamic size of the released exosomes (RE) from hydrogels; (H) Zeta potential evaluation of the RE; (I) SEM assessment of the RE; (J, K) AFM imaging of the RE, *n* = 3; (L) Internalization efficacy of the RE by hepatic stellate cells (LX‐2) at 1 and 3 h, *n* = 3.

After a detailed physicochemical assessment of the bioactive formulations, it was imperative to determine their ability to allow for cellular growth and proliferation. For a sprayable hydrogel application and adherence to any internal organ and subsequent acceptance by the body for therapy, it must be biocompatible. Resazurin and MTT assays showed that P1 (GPH‐0.6%D) and P2 (GPH‐1.2%D) maintained hepatocyte viability comparable to controls, whereas P3 (GPH‐1.8%D) significantly reduced viability by day 5 (*p* < 0.01), indicating growth inhibition beyond 1.2% w/v polydopamine (Figure 4A,B). This trend was additionally reflected in live/dead staining, where robust cell survival was observed for GPH, P1, and P2, but reduced for P3 (Figure S5). Actin/DAPI cytoskeletal staining further revealed healthy, well‐spread morphologies of cells treated with GPH, P1, and P2, underscoring their favorable cell‐material interactions (Figure S5).

Hemocompatibility testing demonstrated negligible hemolysis% for all formulations (GPH: 0.17% ± 0%; P1: 3.17% ± 0.02%; P2: 2.87% ± 0.09%; P3: 3.48% ± 0.09%), in stark contrast to the positive control (Triton X‐100; 100 ± 2.83%), indicating their suitability for blood‐contacting applications (Figure 4C and Figure S5). Based on the bio‐adhesiveness and cytocompatibility assessment modified polymer of P2, along with GPH (as control), was assessed for the functional efficacy of albumin synthesis by the growing cells in the presence of the formulations. This depicted comparable albumin production (∼300 ng/mL on day 5) across all groups, suggesting no significant effect on the cells in the presence of the formulations (Figure S5).

To ensure clinical translatability and long‐term stability, trehalose was incorporated as a cryoprotectant during lyophilization. The resulting freeze‐dried formulation offers a stable and portable format suitable for large‐scale production, transport, and on‐demand clinical use, while enabling reversible sol transition upon rehydration in prewarmed PBS or saline at 37–40°C for 15–20 min (Figure 4D). Furthermore, post‐blue light cross‐linking of the sprayable formulation, the hydrogel allowed for sustained release of encapsulated hUCMSCs‐Exo (∼95% in 28 days, extrapolation of in vivo retention), thereby overcoming the drawbacks associated with burst release of exosomes via conventional delivery methods (Figure 4E).

Stability evaluation of the hydrogels through swelling and degradation studies showed a swelling ratio of ∼9 after 48 h for both P2 and P2 + Exo groups (Figure 4F). In parallel, degradation analysis in PBS revealed that after 28 days, the hydrogels retained 86.02% ± 2.3% (P2) and 91.3% ± 7.75% (P2 + Exo) of their initial weight, underscoring the structural stability and durability of the formulations (Figure 4F and Figure S5). Preservation of the structural and functional integrity of exosomes post‐storage is crucial. DLS and zeta potential analyses showed that released exosomes (RE) maintained a size of 100–120 nm and a negative charge of ∼ −41 mV, comparable to freshly isolated exosomes (FE) (Figure 4G–K).

Consistently, SEM and AFM confirmed similar morphology and dimensions between RE and FE (Figure 4I‐K). Efficient cellular uptake of exosomes by target organ cells is essential for modulating gene and protein expression. Hepatic stellate cells (LX‐2) demonstrated rapid internalization of RE within 1 h, even after 6 months of storage of BioNano spray (Figure 4L). This is also consistent with previous reports demonstrating the efficient internalization of exosomes by multiple hepatic cell populations, including hepatocytes, hepatic stellate cells, and macrophages/Kupffer cells, thereby aiding broad modulation of the liver microenvironment [27, 28, 29, 30, 31]. These findings underscore the preserved bioactivity and sustained release capability of the lyophilized, off‐the‐shelf BioNano spray.

### 2.5 Assessment of the Intrinsic Ability of Biospray and BioNano Spray to Modulate Macrophage Polarization

**FIGURE 5:**
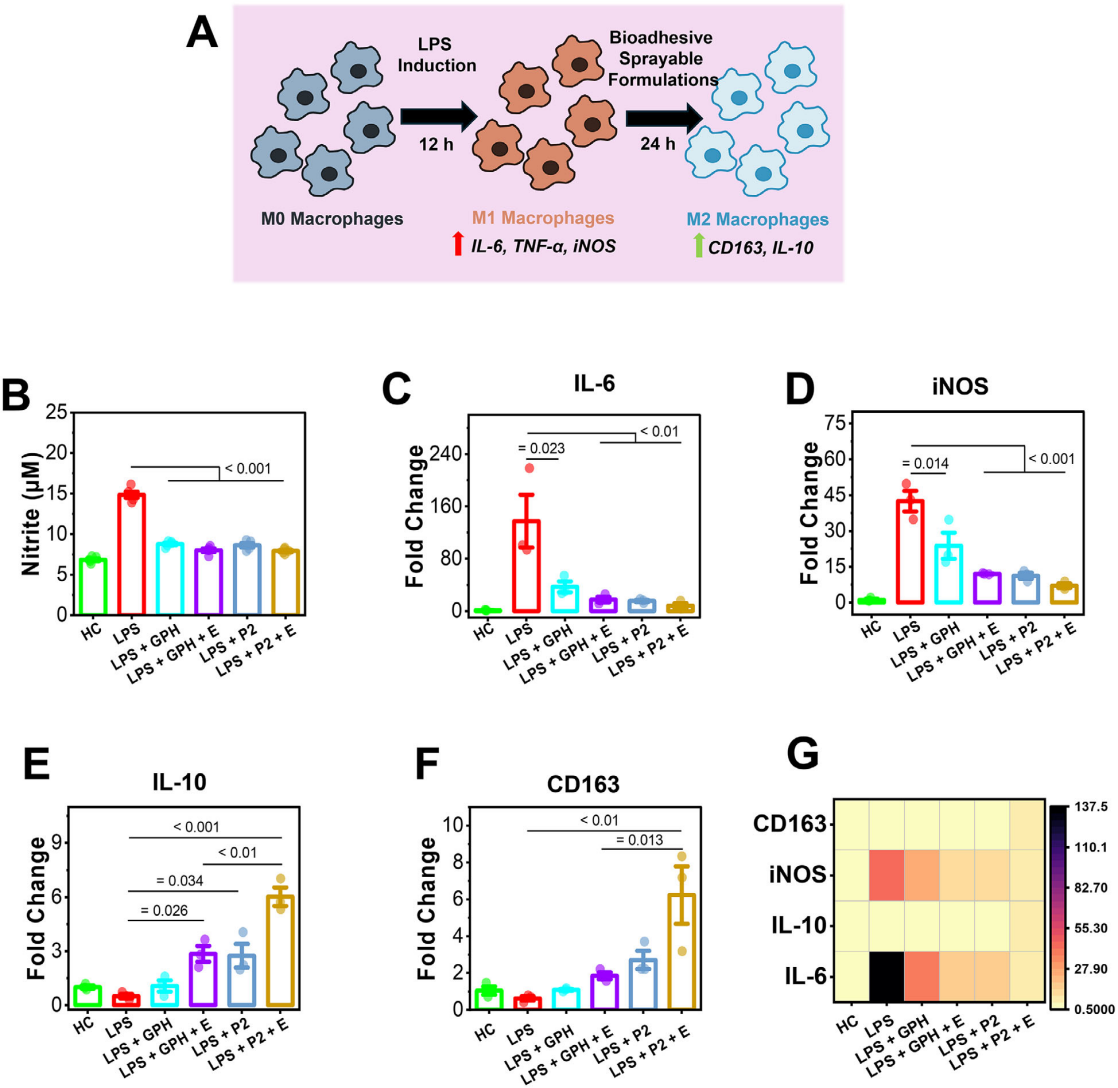
Evaluating the potential of unmodified and polydopamine‐conjugated hydrogel formulations in combination with or without hUCMSCs‐Exo to promote macrophage (RAW 264.7) polarization from M1 to M2 phenotype. (A) Graphical representation of the phenomenon of phenotypic switch of macrophages, key orchestrators of hepatic immune homeostasis, exhibit remarkable plasticity, transitioning between pro‐inflammatory M1 (IL‐6, iNOS) and anti‐inflammatory M2 (IL‐10, CD163) phenotypes associated markers involved with macrophage polarization; (B) Quantification of released nitric oxide using Griess assay, *n* = 3; (C, D) Gene expression analysis of the pro‐inflammatory (IL‐6 and iNOS) marker genes with/without hUCMSCs‐Exo; (E, F) Gene expression changes in anti‐inflammatory cytokine (IL‐10) and M2 macrophage marker CD163. Data is expressed as Mean ± S.E, *n* = 3; (G) Heatmap profile of the assessed genes across several experimental groups.

In the advanced stage of MAFLD, persistent activation of pro‐inflammatory M1 macrophages sustains hepatic injury, whereas a phenotypic switch toward anti‐inflammatory M2 cells promotes resolution and tissue repair [32]. The intrinsic immunomodulatory ability of the P2 formulation as a biospray presents a promising strategy to modulate the immune response through macrophage polarization and anti‐inflammatory effects (Figure 5A).

In this study, to assess the immunomodulation aspect of dopamine‐conjugated formulations, the RAW 264.7 cells were treated with P2 and GPH formulations, with/without the exosomes (rich in bioactive miRNAs and proteins). RAW 264.7 macrophages treated with P2 (GPH‐1.2%D) and GPH, with or without exosomes, revealed via Griess assay that P2 intrinsically suppressed NO production in LPS‐stimulated cells, indicating inherent immunomodulatory potential (Figure 5B).

Gene expression profiling further confirmed this shift: all treatment groups downregulated iNOS and IL‐6 (LPS+GPH: 37.06 ± 8.42, LPS + GPH + Exo: 17.52 ± 4.22, LPS + P2: 15.31 ± 1.75, LPS + P2 + Exo: 8.15 ± 3.75‐fold changes), while upregulating IL‐10 and CD163 (Figure 5C–G). Notably, P2 + Exo induced the highest IL‐10 expression (*p* < 0.01; 6.02 ± 0.51) and CD163 (*p* = 0.013; 6.23 ± 1.56) expression, outperforming GPH + Exo (IL‐10: 2.85 ± 0.44; CD163: 1.84 ± 0.19) (Figure 5C–G).

Morphological analysis supported these findings: F‐actin/DAPI staining revealed that LPS‐stimulated cells became enlarged, elongated, and displayed nuclear distortion, hallmarks of inflammatory activation. Treatment groups, however, showed a marked restoration of normal cell morphology, with P2‐based formulations matching or exceeding the performance of GPH + Exo. P2 + Exo, in particular, demonstrated the most pronounced architectural recovery (Figure S6).

Immunofluorescence analysis and its quantification corroborated these findings; CD163 expression was notably increased, and iNOS reduced across all treatments, with exosomes providing an additive or complementary effect in amplifying P2's effect (Figure S6). Our data suggest that the therapeutic effect is associated with macrophage phenotype switching toward M2, which is known to promote fibrosis resolution. From detailed assessments, P2 formulation with (BioNano spray)/without exosomes (Biospray) was further investigated in a preclinical model of chronic MAFLD.

### 2.6 Preclinical Evaluation of Developed Cell‐Free Sprayable Bio‐Adhesive Formulations to Attenuate Lipid Metabolism Impairment in MAFLD

**FIGURE 6:**
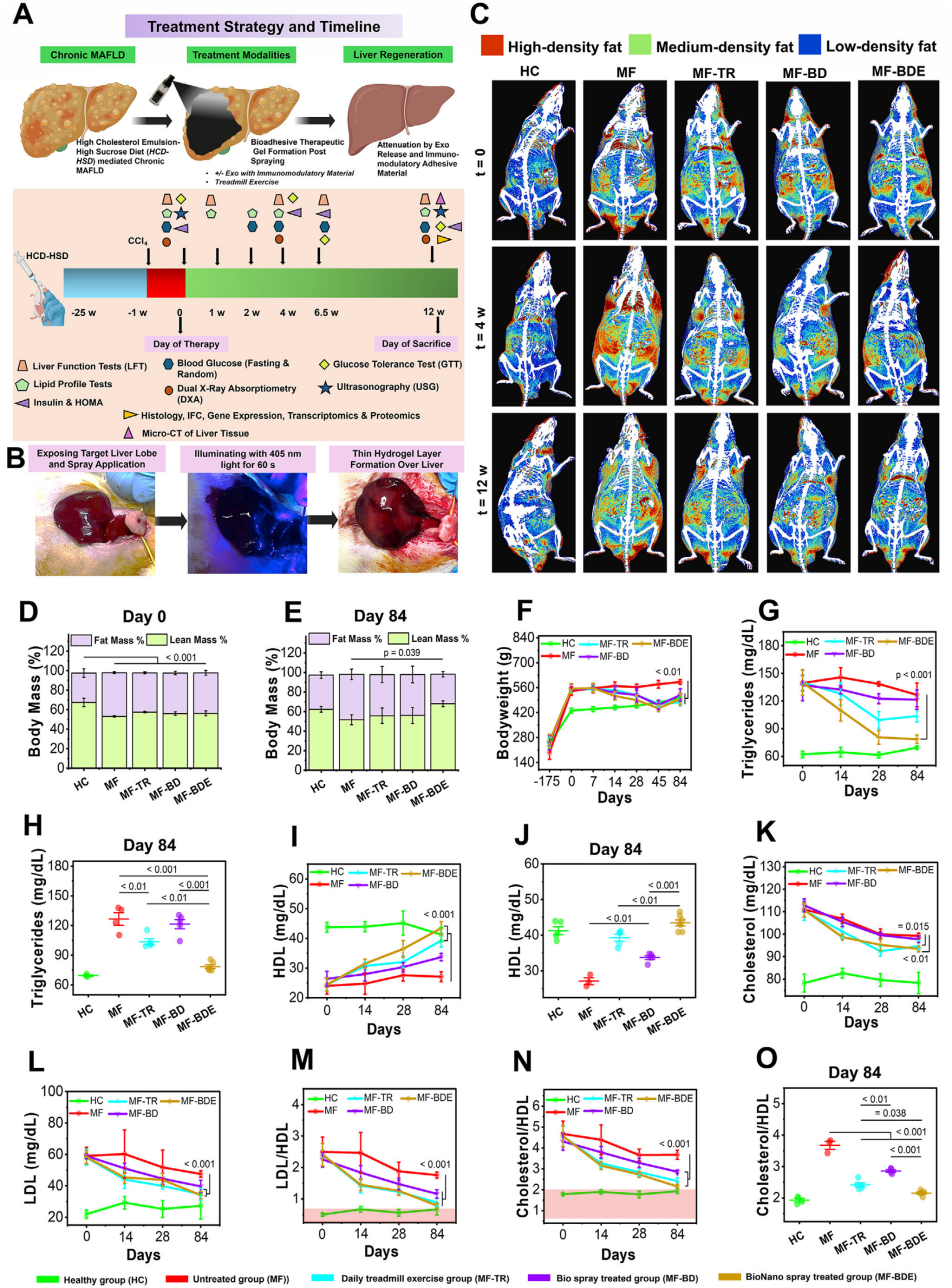
In vivo evaluation of our developed bioengineered intervention to attenuate dysregulated lipid metabolism and biochemical parameters. (A) Schematic representation of the employed laparotomy surgical intervention and timeline for in vivo assessment in the chronic MAFLD animals; (B) Digital images representing the application of adhesive sprays and adherence mediated post‐covalent cross‐linking onto liver tissue; (C) Fat composition analysis on weeks 0, 4, and 12, n ≥ 3; (D, E) Quantitative evaluation of the body mass% of all the treatment groups and controls on week 0 and 12 respectively. Data is expressed as Mean ± S.D, n ≥ 3; (F) Bodyweight profile of the experimental groups over 84 days post therapeutic intervention. Data is expressed as Mean ± S.D, n ≥ 5; (G) Serum triglycerides (TGA) levels of the various groups. Data is expressed as Mean ± S.D, n ≥ 3; (H) Serum TGA level on day 84 of assessment. Data is expressed as Mean ± S.E, n ≥ 3; (I) Serum high‐density lipoprotein (HDL) profile over 84 days. Data is expressed as Mean ± S.D, n ≥ 4; (J) Serum HDL level on day 84 of evaluation. Data is expressed as Mean ± S.E, n ≥ 3; (K) Serum cholesterol levels of the different animal groups over 84 days post‐intervention; (L) Serum low‐density lipoprotein (LDL) levels over 84 days of assessment; (M) Serum LDL/HDL ratio post‐minimally invasive surgical intervention; (N) Cholesterol/HDL ratio of experimental groups over 84 days. Data is expressed as Mean ± S.D, n ≥ 3; (O) Graph depicting the cholesterol/HDL ratio on day 84 of evaluation. Data is expressed as Mean ± S.E, n ≥ 3.

Therapeutic potential of the BioNano spray was assessed in a chronic MAFLD rat model, developed via high‐fat cholesterol emulsion and high‐sucrose diet (HCD‐HSD) for 24 weeks, further aggravated by carbon tetrachloride (CCl_4_)administration (3 mL/kg) for 1 week (enhanced severity from our previous report) [17] (Figure 6A). A 24‐week HCD establishes a robust metabolic disease phenotype (steatosis, inflammation, and insulin resistance), while an additional short‐term, low‐dose CCl_4_ exposure serves to synchronize and modestly accelerate fibrosis progression without overriding the metabolic context. This combined approach enables consistent induction of fibrotic endpoints within a practical timeframe while preserving disease relevance [33, 34]. Animals were then randomly assigned to various experimental groups, followed by localized application of P2 (MF‐BD; Biospray) or P2 + Exo (MF‐BDE; BioNano spray) formulations onto the right liver lobe, followed by 405 nm light illumination for 60 s (Figure 6B). The sprayable nature of the hydrogel formulation enables precise and uniform coverage of the liver surface and is amenable to laparoscopy. However, in the present study, administration was performed via open laparotomy rather than through a laparoscopic approach. Post‐intervention, animals were monitored for 12 weeks, with the pre‐surgical time‐point designated t = 0 (day 0). Experimental design, group allocation, and animal numbers are detailed in Table S2. The regeneration ability of the developed intervention was evaluated by tracking lipid parameters and body composition before and after minimally invasive surgery over 12 weeks.

Dual‐energy x‐ray absorptiometry (DXA) revealed substantial improvements in fat distribution among treated groups, with a marked reduction in red (high‐density fat) and green (medium‐density fat) signals, alongside a decrease in subcutaneous fat as evident in the x‐ray images (Figure 6C). Both treadmill exercise (MF‐TR) and only‐material spray (Biospray; MF‐BD) groups showed comparable reductions, while the BioNano spray‐treated group (MF‐BDE) exhibited a substantial improvement compared to the untreated control (MF). Notably, the intrinsic immunomodulatory properties of bioadhesive spray resulted in at‐par efficacy with the daily exercise positive control group.

Fat mass (%) significantly decreased across all treated groups, whereas lean mass (%) increased, reaching 68.09% ± 3% in MF‐BDE, followed by ∼55% in MF‐TR and MF‐BD, compared to 51% ± 5.19% in MF after 12 weeks (Figure 6D,E). Reduction in bodyweight and attenuation in the serum lipid profile aligned with DXA findings, demonstrating significantly reduced triglyceride levels in all treatment groups, with MF‐BDE (78.58 ± 1.83 mg/dL) outperforming MF‐TR (103.75 ± 2.97 mg/dL) and MF‐BD (121.42 ± 4.63 mg/dL) (Figure 6F–H). HDL, which typically reduces in MAFLD, was significantly elevated in MF‐BDE (43.4 ± 2.16 mg/dL), with a moderate increase observed in MF‐TR (39.2 ± 2.17 mg/dL) and MF‐BD (33.6 ± 1.21 mg/dL) after 84 days (Figure 6I,J). Total cholesterol was seen to reduce in MF‐BDE (*p* < 0.01) and MF‐TR (p = 0.015), with a similar pattern for LDL (Figure 6K,L). Furthermore, cardiovascular risk markers, LDL/HDL and cholesterol/HDL ratios, were substantially lowered, reaching near‐physiological ranges: cholesterol/HDL (2.2–2.3) and LDL/HDL (0.2–0.6), with statistically significant differences between MF‐BDE and MF‐BD (Figure 6M–O).

Collectively, these assessments demonstrate that these outcomes are superior to those achieved via lifestyle interventions alone, which indicates that the therapeutic effect cannot be explained by weight loss or diet control.

### 2.7 Histopathological and Microvasculature Analysis to Assess Efficacy in Restoring Hepatic Microstructure

**FIGURE 7:**
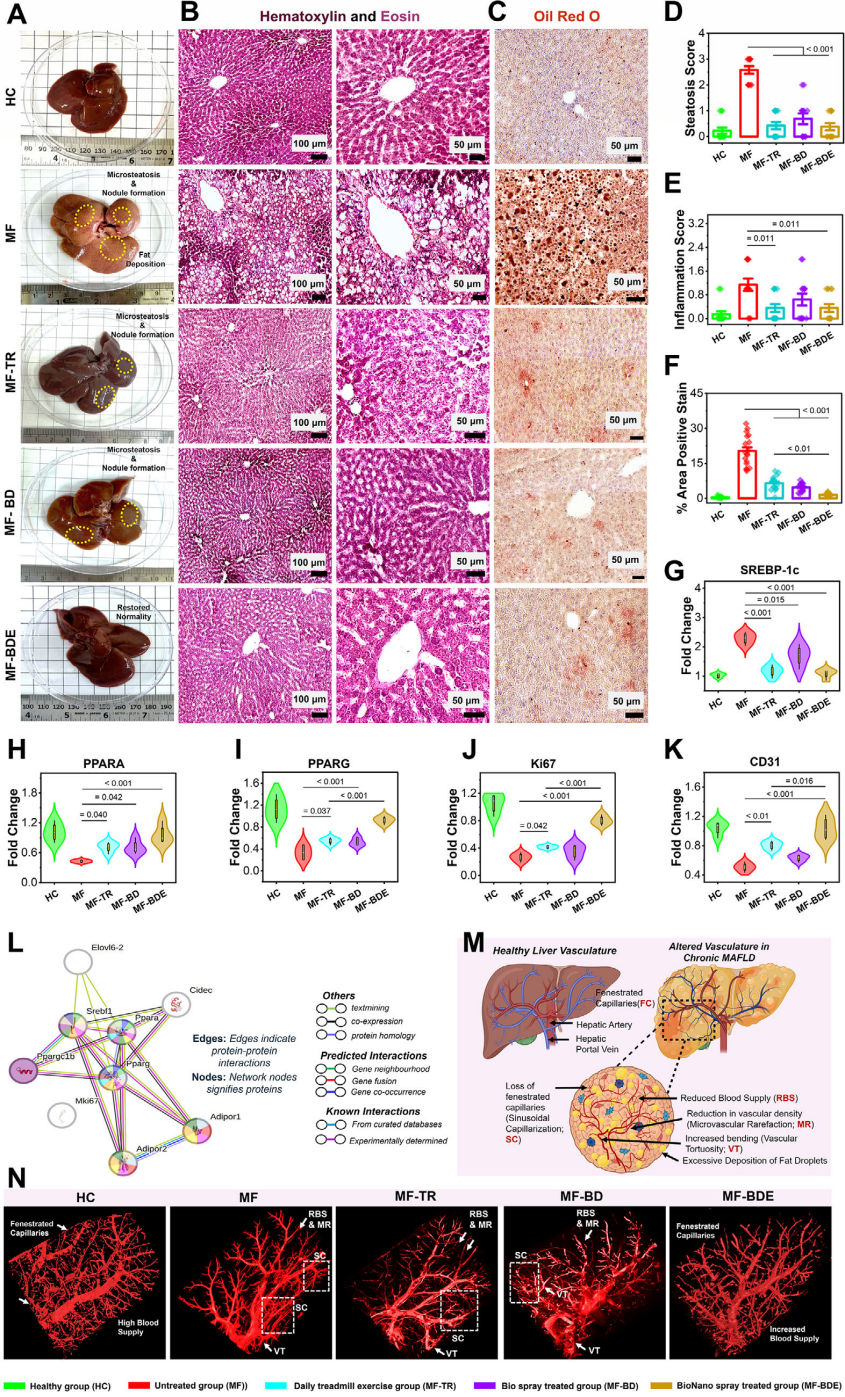
Histopathological assessment and alterations in microvasculature and lipid metabolism genes in the various experimental groups post‐intervention. (A) Digital images depicting the extracted liver tissue after 12 weeks; (B) Hematoxylin and eosin (H&E) staining of the extracted liver tissues; (C) Oil Red O (ORO) staining for visualization of lipid droplets on the tissue of different groups, *n* = 3; (D, E) Quantification of H & E images to evaluate steatosis and inflammation score respectively; (F) ORO images quantification to determine the area positive stain. Data is expressed as Mean ± S.E, n ≥ 10; (G–I) Gene expression changes of critical lipid metabolism regulators, Sterol Regulatory Element‐Binding Protein‐1c (SREBP‐1c), Peroxisome Proliferator‐Activated Receptor Alpha (PPARA), and Peroxisome Proliferator‐Activated Receptor Gamma (PPARG), respectively; (J, K) Gene expression changes in Ki67 and CD31, respectively. Data is expressed as Mean ± S.D, violin depicts the probability density, white dot indicates the median and yellow bar shows the interquartile range (25%–75%), n ≥ 3; (L) Deciphering the calculated protein‐protein interactions between proteins of the differentially expressed lipid metabolism genes and associated proteins, using the STRING database; (M) Schematic illustration of the microvasculature alterations during progressive MAFLD and liver fibrosis. In this pathological state, lipid‐laden hepatocytes and progressive fibrosis compress the sinusoidal architecture, leading to diminished vascular density and compromised hepatic perfusion. Sinusoidal endothelial cells lose their fenestrations and acquire basement membrane deposition, hallmarks of capillarization, thereby impairing bi‐directional exchange between blood and hepatocytes. Although hypoxia‐driven VEGF signaling is activated, the ensuing angiogenesis is aberrant, resulting in disorganized and tortuous neovessels with limited functional efficacy; (N) Micro‐CT assessment of liver microvasculature of the extracted right lobe for the various groups, *n* = 3.

Chronic MAFLD is defined by hallmark histopathological features, including hepatocyte ballooning, steatosis, inflammatory infiltration, peripheral nuclear displacement, and intracellular lipid accumulation [35]. After 12 weeks of intervention, gross morphological examination revealed marked attenuation of microsteatosis and nodular lesions in all treated groups compared with untreated MF controls (Figure 7A). In vivo degradation assessment demonstrated that by 30 days post‐application, only minimal residual hydrogel was detectable, confirming its efficient biodegradation under physiological conditions (Figure S7). Histopathological analysis by hematoxylin and eosin (H&E) staining confirmed these improvements, showing reduced hepatocyte ballooning, diminished steatosis, and attenuated inflammatory cell infiltration across different liver lobes in the experimental groups (Figure 7B and Figure S8). Among the interventions, the BioNano spray‐treated group (MF‐BDE) achieved the most complete architectural restoration, with preserved lobular organization and minimal cytoplasmic vacuolation (Figure 7B). Histological scoring (CRN criteria, pathologist‐blinded scoring) showed that steatosis was reduced by ∼85% and inflammation by ∼70% in the BioNano spray‐treated group (MF‐BDE: steatosis: 0.38 ± 0.14; inflammation: 0.36 ± 0.13) compared with negative controls MF (2.58 ± 0.15 and 1.14 ± 0.21, respectively; *p* < 0.001) (Figure 7D,E). The Biospray alone (MF‐BD) and lifestyle intervention (MF‐TR) groups also improved, with MF‐BD showing steatosis of 0.69 ± 0.2 and MF‐TR 0.42 ± 0.14, though both remained less effective than MF‐BDE. Additionally, H&E analysis of kidney and spleen tissues at 2 and 12 weeks post‐treatment demonstrated preserved normal tissue architecture in the BioNano spray‐treated group (MF‐BDE), comparable to healthy controls, indicating the absence of significant short or long‐term systemic toxicity (Figure S9). Oil Red O staining and quantification further confirmed lipid clearance, with the MF‐BDE group exhibiting ∼77% lower lipid area (1.5% ± 0.2%) than MF‐TR (6.49% ± 0.78%; *p* < 0.01) and ∼68% lower than MF‐BD (4.69% ± 0.43%) (Figure 7C,F).

At the molecular level, MAFLD progression is associated with upregulation of SREBP‐1c and suppression of PPARA and PPARG. One‐time sprayable bioadhesive administration downregulated SREBP‐1c across all groups, with the most pronounced suppression in BioNano spray‐treated group, MF‐BDE (∼2.5‐fold reduction vs. untreated animals), while PPARA and PPARG expression were significantly restored, indicating transcriptional reactivation of lipid oxidation and metabolic homeostasis. BioNano spray treatment (MF‐BDE) outperformed the treadmill exercise group; MF‐TR (*p* < 0.001), highlighting its combinatorial advantage (Figure 7G–I and Table S8). Given that chronic MAFLD often transitions to MASH via impaired hepatocyte regeneration and sinusoidal microvascular loss, we assessed Ki67 and CD31 expression. MF‐BDE restored both markers to near‐healthy levels (*p* < 0.001), suggesting recovery of regenerative capacity and endothelial fenestration. MF‐BD and MF‐TR showed similar but less complete improvements (Figure 7J,K).

To resolve regulatory mechanisms, we performed STRING‐based PPI analysis of differentially expressed genes, revealing a highly interconnected network (9 nodes, 19 edges, local clustering coefficient = 0.746) enriched in adiponectin signaling, insulin resistance modulation, AMPK pathway activation, and regulation of triglyceride sequestration and fatty acid metabolism. Central network hubs included CIDE‐3, ADIPOR1, and ADIPOR2, representing potential co‐regulatory targets for enhanced metabolic correction (Figure 7L and Figure S10). PPI‐based insights provide a system‐level understanding of the mechanistic underpinnings of the observed phenotypic improvements, underscoring the multifactorial benefits of our engineered strategy.

Progressive MAFLD, accompanied by extensive hepatic microvascular remodeling, is characterized by sinusoidal rarefaction, capillarization, and endothelial dysfunction (detailed in the legend of Figure 7M). Micro‐CT revealed severe microvascular abnormalities in negative controls (MF), including sinusoidal collapse, rarefaction, capillarization, and tortuous neovessels, features consistent with aberrant, hypoxia‐driven angiogenesis. Treadmill (MF‐TR) and Biospray (MF‐BD) groups showed partial restoration, with reduced but persistent vascular rarefaction and tortuosity. This suggests that the only material‐based therapy and lifestyle intervention also modulate lipid metabolism homeostasis to a certain extent. In contrast, BioNano spray group (MF‐BDE) exhibited near‐complete recovery, marked by higher vascular density, reappearance of fenestrated capillaries, and improved perfusion, indicating effective reversal of endothelial dysfunction (Figure 7N). These findings underscore the capacity of BioNano spray to restore microvascular integrity and potentially interrupt the self‐perpetuating cycle of hypoxia, inflammation, and fibrosis in advanced MAFLD.

Taken together, these histological findings, gene expression studies, PPI analysis, and microvasculature assessment, when integrated with systemic lipid profile data and body composition analyses, provide compelling evidence of the potential of our developed minimally invasive strategy of BioNano spray in mitigating lipid accumulation and restoring microarchitectural integrity. The therapeutic benefits likely arise from the combined anti‐inflammatory and pro‐regenerative actions of the bioactive hydrogel and exosomal cargo (identified proteins and miRNAs), which together modulate lipid metabolism pathways.

### 2.8 In vivo Efficacy of Therapeutic Strategies in Mitigating Oxidative Stress and Restoring Liver Function

**FIGURE 8:**
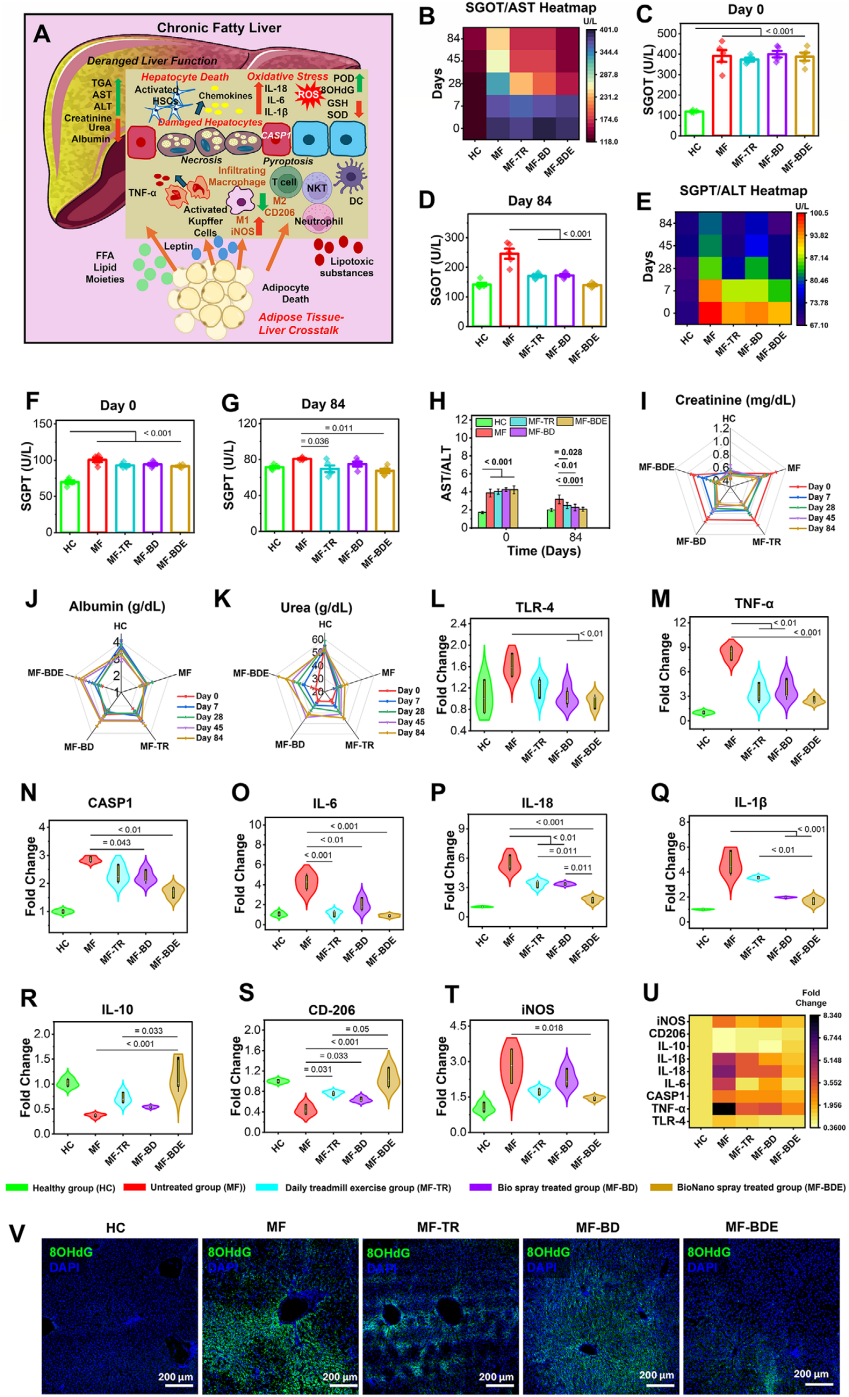
Assessing biochemical parameters (liver function test; LFT) and genes and proteins associated with liver injury. (A) Schematic illustration of chronic MAFLD‐induced inflammation and oxidative stress resulting in an altered LFT profile. Pathophysiologically, excess free fatty acids (FFAs) fuel ROS overproduction in hepatocytes, activating resident Kupffer and hepatic stellate cells (HSCs). This initiates an inflammatory fibrogenic cascade, marked by pro‐inflammatory macrophage infiltration and upregulation of cytokines (IL‐6, IL‐1β, IL‐18, TNF‐α); (B) Heatmap representation of changes in AST/SGOT levels over 84 days; (C) AST levels on day 0; (D) AST levels on day 84 of evaluation. Data is expressed as Mean ± S.E, n ≥ 4; (E) Heatmap illustration of changes in ALT/SGPT levels in experimental groups over 84 days; (F, G) ALT levels on day 0 and 84, respectively. Data is expressed as Mean ± S.E, n ≥ 4; (H) AST/ALT ratio on days 0 and 84 post‐therapeutic intervention. Data is expressed as Mean ± S.D, *n* = 5; (I) Serum creatinine levels over 84 days. Data is expressed as Mean ± S.D, n ≥ 5; (J, K) Changes in serum albumin and urea levels in the various groups over 84 days, respectively. Data is expressed as Mean ± S.D, n ≥ 4; (L–Q) Gene expression variations of pro‐inflammatory genes (TLR‐4, TNF‐α, CASP1, IL‐6, IL‐18, and IL‐1β) associated with liver injury and disease progression; (R‐T) Changes in the expression of critical genes involved with anti‐inflammation and macrophage polarization (IL‐10, CD206, and iNOS); Data is expressed as Mean ± S.D, violin depicts the probability density, white dot indicates the median and yellow bar shows the interquartile range (25%–75%), n ≥ 3; (U) Heatmap depiction of the changes in genes associated with inflammation, oxidative stress, and macrophage polarization, across the experimental groups; (V) Immunofluorescence assessment of oxidative stress marker of 8‐hydroxy‐2’‐deoxyguanosine, *n* = 3.

Advanced MAFLD is characterized by hepatocellular injury and systemic metabolic imbalance, manifested by elevated serum AST, ALT, and creatinine, along with reduced albumin and urea levels [36, 37, 38] (Figure 8A).

Day 0 (before intervention) indicated abnormal liver injury parameters with AST (391.3 ± 28.9 U/L) and ALT (100.45 ± 3.11 U/L) significantly elevated relative to healthy controls (HC). By day 84, liver injury parameter (AST) was restored toward healthy levels in all treated groups, with BioNano spray‐treated group (MF‐BDE) achieving the most pronounced decline (139.7 ± 3.26 U/L, *p* < 0.001) (Figure 8B–D and Figure S11). ALT levels followed a similar trend, with MF‐BDE showing a significant decrease by day 28 (71.7 ± 3.8 U/L, p = 0.032) and further improvement by day 84 (67.4 ± 2.4 U/L, *p = 0.011*), followed by MF‐TR and MF‐BD (Figure 8E–G and Figure S11). The difference between the biomaterial‐only (MF‐BD) and hydrogel‐exosome (BioNano spray; MF‐BDE) groups was not statistically significant for ALT at week 12, indicating that the biomaterial itself had a strong effect on this measure. Normalization of AST/ALT ratios across all interventions indicated a transition toward hepatic recovery (Figure 8H). All treatment modalities improved the liver functioning to some degree by day 84, but the BioNano spray group containing exosomes achieved the largest improvement and the earliest improvement. Serum creatinine, initially elevated (0.93 ± 0.06 mg/dL), declined significantly in MF‐BDE by day 45 (0.55 ± 0.06 mg/dL), with MF‐BD (0.62 ± 0.05 mg/dL, *p = 0.027*) outperforming lifestyle intervention (MF‐TR) (Figure 8I). Liver synthetic capacity also improved, as reflected by higher albumin and urea in MF‐BDE (3.6 ± 0.26 g/dL and 51.2 ± 6 mg/dL, respectively), approaching HC values. Interestingly, MF‐BD (only material; Biospray) produced biochemical improvements comparable to MF‐TR, highlighting the intrinsic therapeutic capacity of the biomaterial platform (Figure 8J,K).

To explore immunometabolic regulation, pro‐ and anti‐inflammatory gene expression was profiled. The untreated MAFLD group (MF) displayed marked upregulation of TLR4 (∼1.6‐fold) and TNF‐α (∼8.5‐fold) compared to HC, indicating elevated innate immune activation. These were significantly suppressed (*p* < 0.01) in all treated groups, with MF‐BDE and MF‐BD reaching near‐baseline levels, followed by MF‐TR (Figure 8L,M). CASP1 expression emulated this trend, reinforcing the anti‐inflammatory potential of the interventions (Figure 8N). Chronic inflammation mediators IL‐6, IL‐18, and IL‐1β were markedly downregulated in all groups, with IL‐1β levels significantly lower in MF‐BDE than MF‐TR (p < 0.01), suggesting stronger immunomodulation by MF‐BDE (Figure 8O–Q).

Notably, the BioNano spray‐treated group (MF‐BDE) exhibited elevated IL‐10 levels, consistent with a protective anti‐inflammatory milieu (Figure 8R). Macrophage polarization analysis revealed a marked shift toward the reparative M2 phenotype, indicated by increased CD206 expression in MF‐BDE, accompanied by significant downregulation of the pro‐inflammatory M1 marker iNOS (p = 0.018) (Figure 8S–U). A similar trend was observed in the immunostaining for 8OHdG, where the BioNano spray group exhibited notably reduced oxidative stress marker expression compared to the other treatment groups (Figure 8V). Collectively, these results indicate that the sprayable therapy not only restored liver function and metabolic balance but also rewired the inflammatory microenvironment, offering a multifaceted and holistic strategy against advanced MAFLD, in comparison to treadmill exercise and only‐material (Biospray) groups. It is noteworthy that even the only‐material spray (without exosomes) conferred significant benefit, which highlights the expected intrinsic anti‐inflammatory nature of the hydrogel.

### 2.9 Efficacy of our Developed Minimally Invasive Intervention to Alleviate MAFLD‐Associated Progressive Liver Fibrosis

**FIGURE 9:**
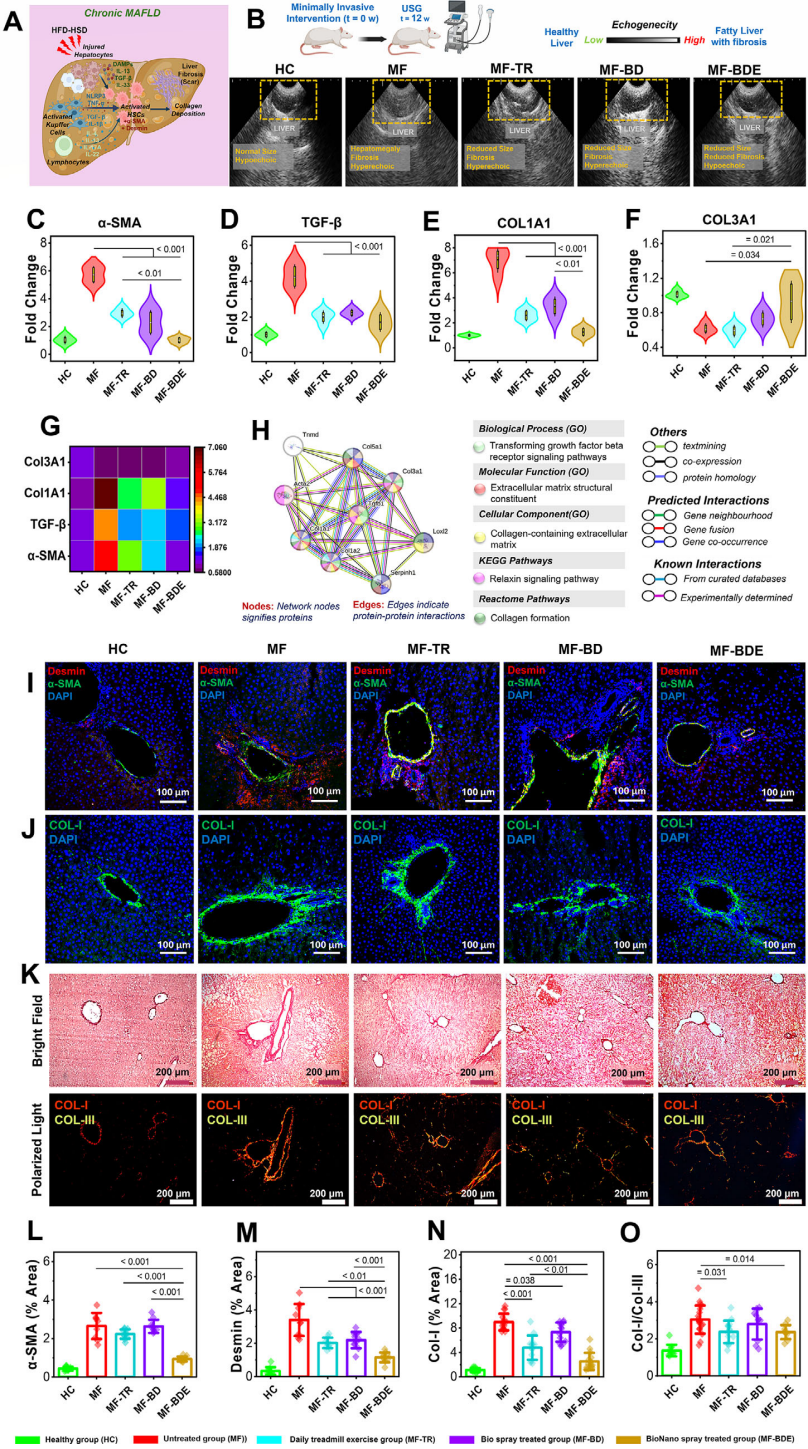
Assessment of fibrosis reversal following bioengineered minimally invasive strategy. (A) Schematic representation of the mechanism of progressive MAFLD‐associated fibrosis; (B) Ultrasound imaging (USI) of the animals to evaluate liver fibrosis and fatty liver grade, *n* = 3; (C–F) Changes in the gene expression profile of critical regulators (α‐SMA, TGF‐β, COL1A1, COL3A1) of liver fibrosis, Data is expressed as Mean ± S.D, violin depicts the probability density, white dot indicates the median and yellow bar shows the interquartile range (25%–75%), n ≥ 3; (G) Heatmap representation of the changes in gene expression profile across various groups; (H) STRING database analysis to predict protein‐protein interactions based on the differential expression pattern of fibrotic genes and other related proteins; (I) Immuno‐fluorescence imaging of characteristic marker (α‐SMA/Desmin) of activated hepatic stellate cells (promote fibrosis), *n* = 3; (J) Expression of Col‐1 protein in different groups, *n* = 3; (K) Picrosirius staining to assess collagen distribution under bright‐field and polarized light, *n* = 3; (L) Quantification of α‐SMA protein expression; (M) Quantification of desmin expression from immuno‐stained images; (N) Quantification of collagen‐I positive stained area in all the experimental groups; (O) Col‐I/Col‐III ratio quantified from picrosirius stained images. Data is expressed as Mean ± SE, n ≥ 7.

Chronic inflammation plays a pivotal role in MAFLD progression, driving hepatic stellate cell (HSC) activation and excessive collagen type I (Col‐1) deposition, ultimately leading to fibrotic scarring of liver tissue [39]. The extent of fibrosis is considered a key predictor of liver‐related morbidity and mortality in MAFLD [40, 41, 42] (Figure 9A). Fibrosis regression and hepatic stellate cell (HSC) modulation were assessed via a series of experiments, starting with ultrasound imaging which demonstrated hepatomegaly with hyperechoic lesions, transcriptional upregulation of TGF‐β, α‐SMA, COL1A1, and COL3A1, and elevated COL1A1/COL3A1 ratios in negative control group (MF), consistent with active fibrogenesis. The MF‐BDE group (treated with BioNano spray) demonstrated marked reduction in echogenicity, liver size, and profibrotic gene expression, achieving near‐normal COL1A1 suppression (∼6‐fold vs. MF, *p* < 0.001) and ∼5‐fold α‐SMA reduction. MF‐BD and MF‐TR induced partial improvements, with persistent hyperechogenicity and residual lesions (Figure 9B–G).

PPI analysis (STRING, score > 0.9) revealed a tightly clustered network (9 nodes, 33 edges) enriched for collagen fibril formation, ECM organization, metabolic regulation, and TGF‐β signaling, with central hubs including Col1a2, Col5a1, Loxl2, Serpin H1, and Tnmd (Figure 9H, Figure S12 and Table S9). Immunostaining revealed ∼65% reduction in HSC activation (α‐SMA: 0.93% ± 0.04% in MF‐BDE vs. 2.66% ± 0.2% in MF; *p* < 0.001) and near‐baseline desmin levels in MF‐BDE‐treated livers. Collagen‐I deposition was reduced by ∼72% (2.5% ± 0.36% in MF‐BDE vs. 8.99% ± 0.35% in MF), confirming marked attenuation of fibrosis (Figure 9I,J, L–N). Picrosirius red staining demonstrated a matrix shift toward collagen‐III enrichment, with MF‐BDE showing the lowest Col‐I/Col‐III ratio (2.36 ± 0.11; p = 0.014), indicative of a reparative ECM phenotype (Figure 9K,O). Collectively, these multi‐modal data provide compelling evidence that the BioNano spray effectively modulates key fibrogenic pathways, suppresses HSC activation, and restores ECM homeostasis, thus offering a promising therapeutic avenue for reversing liver fibrosis in MAFLD. Interestingly, only material and lifestyle intervention also resulted in notable attenuation of the degree of fibrosis, highlighting the beneficial outcome of targeting localized inflammation, resulting in not only regulating the systemic inflammatory cascade but also downregulating the activated fibrogenic pathways. Addition of exosomes, further enhanced outcomes, for instance, a higher abundance of miR‐100‐5p in our exosomes, acts as a key regulator, mitigating systemic inflammation and fibrosis by targeting the mTOR‐NF‐κB axis [43, 44]. Suppression of mTOR signaling downregulated pro‐inflammatory mediators (TNF‐α, IL‐6, IL‐1β, iNOS) and fibrotic genes. The developed integrative therapy comprising FDA‐approved immunomodulatory material and therapeutic exosomes attenuates inflammatory signaling, lipid accumulation, and downstream fibrotic remodeling in MAFLD. This also suggests that exosome uptake by Kupffer cells may contribute to immunomodulation and polarization toward an anti‐inflammatory M2 phenotype, thereby attenuating hepatic stellate cell activation and fibrosis progression, consistent with previous reports highlighting the therapeutic significance of targeting Kupffer cell‐mediated pathways in liver fibrosis [45, 46, 47].

### 2.10 Bioadhesive Sprayable Hydrogels Restored Glucose and Insulin Metabolism Impairments

Impaired glucose metabolism and persistent insulin resistance are characteristic co‐morbidities of MAFLD, though the temporal relationship between hepatic steatosis and insulin resistance remains debated [48, 49]. Disrupted liver‐adipose‐pancreas communication impairs insulin clearance, elevating circulating glucose and increasing FFAs, which further exacerbate hepatic lipotoxicity and inflammation. This cascade induces glucotoxicity, oxidative stress, and mitochondrial dysfunction, impairing insulin resistance and insulin sensitivity [50, 51].

**FIGURE 10:**
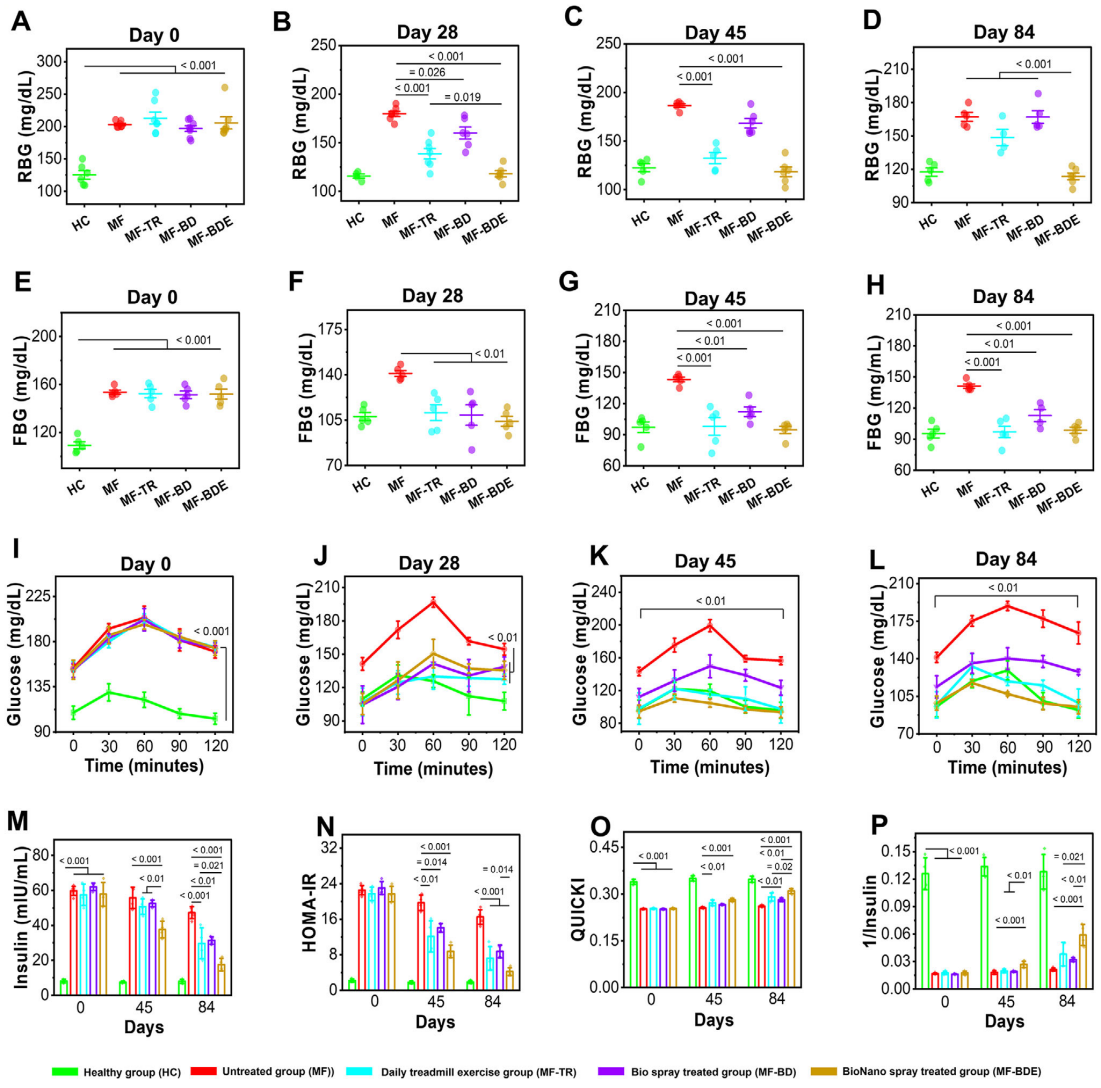
Evaluating the glucose and insulin metabolism parameters post‐intervention. (A–D) Random blood glucose (RBG) levels in the experimental groups over 84 days. Data is expressed as Mean ± S.E., n ≥ 4; (E–H) Fasting blood glucose (FBG) levels on days 0, 28, 45, and 84, respectively. Data is expressed as Mean ± S.E., *n* = 5; (I–L) Glucose tolerance test (GTT) assessment of the different animal groups over 84 days; Data is expressed as Mean ± S.D., *n* = 5; (M) Changes in the fasting insulin levels on days 0, 45, and 84; (N) Homeostatic model‐derived insulin resistance (HOMA‐IR) parameters over 84 days; (O) Quantitative insulin sensitivity check index (QUICKI) over 84 days post‐intervention; (P) Insulin sensitivity parameter expressed by 1/Insulin ratio of all the experimental groups. Data is expressed as Mean ± S.D., n ≥ 4.

Random blood glucose (RBG) levels were reduced by approximately 29% in the BioNano spray‐treated group (MF‐BDE: 113 ± 3.77 mg/dL vs. 167.2 ± 3.29 mg/dL in negative control; *p* < 0.001), 17% in the treadmill exercise group (MF‐TR: 148.7 ± 7.34 mg/dL), and 4% in material‐only (MF‐BD: 167 ± 5.67 mg/dL) (Figure 10A–D and Figure S13). In contrast, lifestyle intervention (MF‐TR; *p* < 0.001) and bioengineered therapies, MF‐BD (p = 0.026) and MF‐BDE (*p* < 0.001), restored glycemic control as early as day 28 in comparison to respective levels of MF (RBG: MF‐TR: 137.7 ± 7.11 mg/dL, MF‐BD: 160 ± 5.93 mg/dL, MF‐BDE: 118 ± 3.9 mg/dL) (Figure 10A–H). Parallel reductions in fasting blood glucose (FBG) (*p* < 0.01) reaffirmed the therapeutic efficacy of these interventions in correcting metabolic dysfunction, with BioNano spray treatment (MF‐BDE) and lifestyle intervention (MF‐TR) exhibiting the most pronounced benefits (Figure 10E‐H).

Oral glucose tolerance test (GTT) further substantiated the improvements in glucose metabolism. At baseline (day 0), all the diseased animals assigned to different groups exhibited impaired glucose clearance within 120 min (Figure 10I). However, by day 45, all the intervened groups demonstrated restored glucose tolerance, with MF‐BDE and MF‐TR showing near‐healthy glycemic responses (Figure 10J,K). Notably, the untreated MF group not only displayed elevated blood glucose but also failed to clear GTT even on day 84, despite cessation of the HCD‐HSD regimen, highlighting the persistence of metabolic dysfunction in the absence of therapeutic intervention (Figure 10L). This also indicated the beneficial role of lifestyle modification (daily exercise) and one‐time application of bioengineered intervention to control and regulate the glucose metabolism homeostasis. Moreover, the data suggest that targeted delivery of anti‐inflammatory, immunomodulatory, biomaterial‐based hUCMSC‐derived exosomes can mitigate inflammation and oxidative stress, thereby stabilizing liver‐pancreas‐adipose tissue crosstalk.

Progression of MAFLD is often characterized by persistent hyperinsulinemia, diminished insulin sensitivity, and compensatory insulin resistance to counteract glucose dysregulation. Insulin concentrations remained persistently elevated in the negative control group (MF) across all time points (55.7 ± 6.1 mIU/mL at day 45; Figure 10M). In contrast, BioNano spray treatment (MF‐BDE) reduced insulin levels drastically. By day 84, additional treatment groups also showed substantial attenuation of hyperinsulinemia, although levels remained higher than those observed in MF‐BDE (Figure 10M). Only with exosomes did HOMA‐IR improve to near‐normal levels, suggesting that exosomal proteins and miRNAs provided an added metabolic regulatory effect (Figure 10N). Insulin sensitivity, assessed by QUICKI (physiological range: 0.35–0.45) and 1/insulin, revealed a marked increase in QUICKI values for MF‐BDE (*p* < 0.01; 0.28 ± 0.005), MF‐TR (0.27 ± 0.002), and MF‐BD (0.26 ± 0.002) on day 45. By day 84, QUICKI values in MF‐BDE converged toward those of MF‐TR and MF‐BD (*p* < 0.01), remaining substantially higher than in the negative control MF (Figure 10O,P). However, the exosome‐loaded spray generally outperformed the matrix alone on key metrics (such as greater insulin‐sensitivity improvements and glucose metabolism). All these evaluations signified the potential role of immunomodulation to restore the glucose and insulin dysregulations, as demonstrated by Biospray and BioNano spray treatments, along with the positive influence of lifestyle modification to restore glucose and insulin imbalance.

### 2.11 Assessing the Ability of Bioengineered Intervention to Modulate the Gut‐Liver Axis Dysbiosis

**FIGURE 11:**
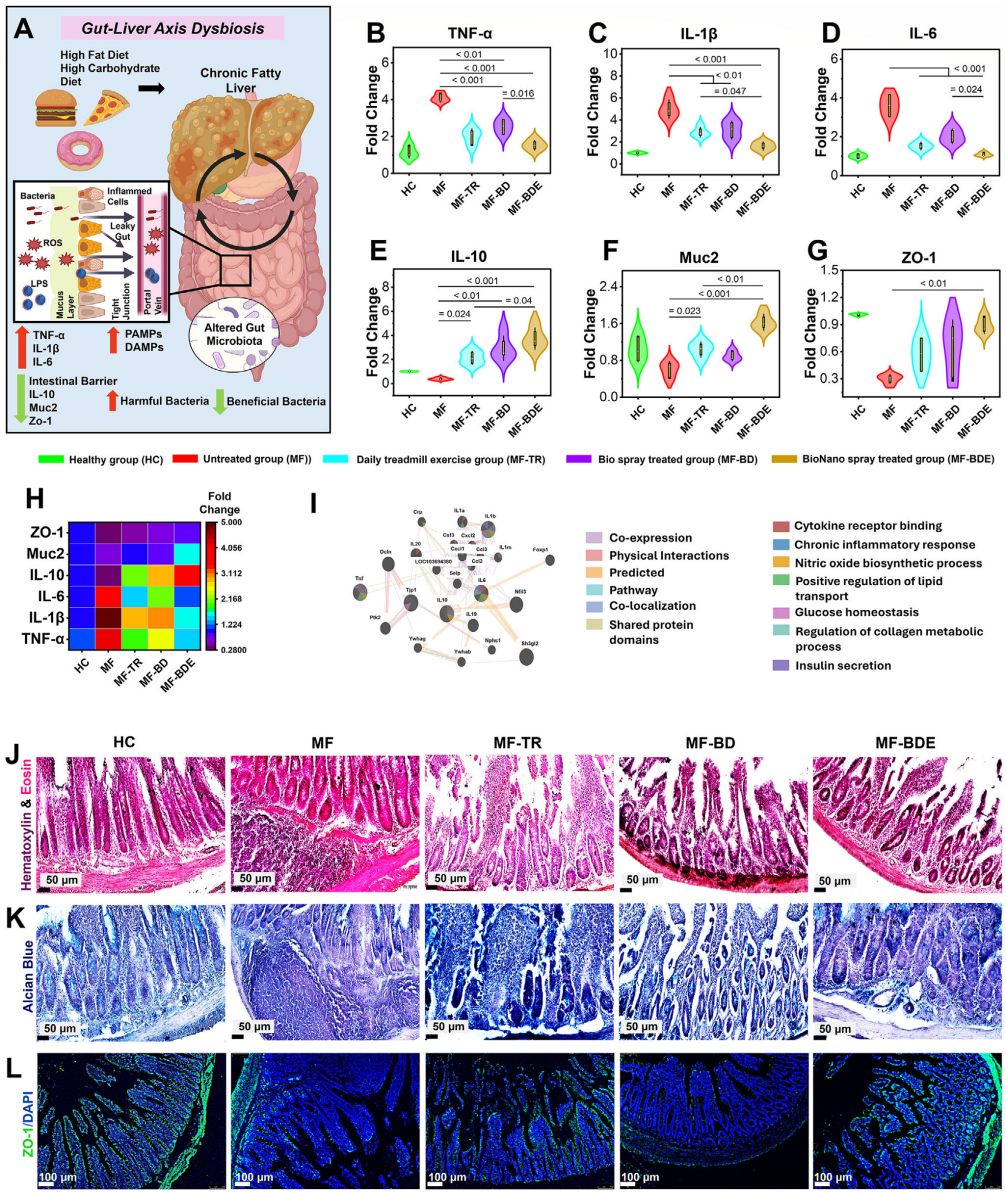
Evaluating the potential of developed therapeutic intervention to modulate the gut‐liver crosstalk. (A) Schematic illustration of altered gut barrier integrity and microbiome via gut‐liver axis, where microbial dysbiosis, particularly within the distal ileum, compromises intestinal epithelial integrity, commonly referred to as a “leaky gut”, and facilitates the translocation of pathogen‐associated molecular patterns (PAMPs), including lipopolysaccharides (LPS), bacteria, and reactive oxygen species (ROS), into the portal circulation. This translocation acts as a potent trigger for hepatic innate immune activation, predominantly via toll‐like receptor (TLR) signaling pathways, sustaining chronic low‐grade inflammation across both hepatic and intestinal compartments. In parallel, alterations in microbial composition and metabolite profiles, such as bile acids and short‐chain fatty acids, further disrupt hepatic metabolic homeostasis and contribute to immune dysregulation, reinforcing the inflammatory, fibrotic cascade characteristic of advanced MAFLD; (B–D) Gene expression alterations of pro‐inflammatory cytokines in the ileum (TNF‐α, IL‐1β, and IL‐6 in the experimental groups, respectively; (E) Expression changes of anti‐inflammatory cytokine, IL‐10; (F‐G) Changes in the genes (Muc2 and ZO‐1) modulating mucin layer and gut‐barrier integrity respectively, Data is expressed as Mean ± S.D, violin depicts the probability density, white dot indicates the median and yellow bar shows the interquartile range (25%–75%), *n* = 3; (H) Heatmap representing the gene expression alterations across the experimental groups; (I) GeneMANIA analysis of the differentially expressed genes to modulate related genes and proteins; (J) H&E staining of the ileum of different experimental groups; (K) Alcian Blue staining revealing the mucin layer alterations in the ileum; (L) Alterations in the protein expression of tight junction protein, Zonula Occludens‐1 (ZO‐1), *n* = 3.

Emerging evidence underscores the pivotal role of gut‐liver axis dysregulation in the pathogenesis and progression of chronic MAFLD [52, 53, 54] (Figure 11A). In our study, it was observed that the treated groups with BioNano Spray (MF‐BDE) and only material Biospray (MF‐BD) demonstrated a multifaceted role in modulating key markers associated not only with gut inflammation but also with mucosal integrity. Pro‐inflammatory cytokines TNF‐α, IL‐1β, and IL‐6 showed pronounced levels in the ileum of untreated animals (MF). On the other hand, MF‐BDE illustrated notable (*p* < 0.001) lower levels, values approaching the healthy gut expression levels. Surprisingly, the treadmill exercise group (MF‐TR) and MF‐BD demonstrated comparable outcomes toward attenuating inflammatory cytokine levels (Figure 11B–D). Furthermore, the anti‐inflammatory cytokine level of IL‐10 was also significantly upregulated (*p* < 0.001) in the combinatorial therapy group of BioNano spray (MF‐BDE, ∼9 fold to negative controls), in comparison to other treatment modalities (∼1.5 fold higher to treadmill exercise group and Biospray). This suggests the possible role of treating the liver directly can alleviate the gut inflammation acting via the gut‐liver axis (Figure 11E). Mucin (Muc2) (*p* < 0.001) and zonula occludens‐1 (ZO‐1) expression (*p* < 0.01) were interestingly notably upregulated in MF‐BDE (∼2.7‐fold higher Muc2 expression; ∼3‐fold higher ZO‐1 expression) compared to untreated animals (MF). Similarly, the lifestyle intervention group also exhibited recovery comparable to one‐time application of only hydrogel spray devoid of exosomes (Biospray), although with a lower effect than BioNano spray (*p* < 0.01) (Figure 11F–H). A heatmap profile illustrates the differential gene expression profile across different experimental groups (Figure 11H). To identify additional gene targets influenced by the intervention, network analysis using GeneMANIA integrated co‐expression, physical interactions, and pathway mapping revealing a cluster of co‐regulated genes enriched in cytokine receptor binding, inflammatory response, nitric oxide synthesis, lipid transport, glucose homeostasis, insulin secretion, and collagen metabolism. These assessments suggest that the therapeutic effects extend beyond immune‐mediated gut barrier restoration to encompass metabolic regulation across the liver‐pancreas and liver‐adipose axes (Figure 11I).

These findings were corroborated by histological analyses, which revealed pronounced inflammation in the distal ileum of the MF group (untreated animals), characterized by dense immune cell infiltration. In contrast, treated groups exhibited marked restoration of ileal microarchitecture, with the MF‐BDE group showing the most prominent recovery, including well‐preserved villi, lamina propria, and crypt structures (Figure 11J). Alcian Blue staining revealed reduced Muc‐2 expression and mucin layer disruption in the negative controls (MF), whereas treatment, particularly with the BioNano spray (MF‐BDE), markedly restored mucosal integrity (Figure 11K). Consistently, ZO‐1 immunostaining showed severe tight junction loss in MF animals, with substantial recovery following MF‐BDE treatment. Notably, lifestyle intervention alone (MF‐TR) failed to re‐establish gut‐liver axis homeostasis, underscoring the superior therapeutic efficacy of the combinatorial regimen (Figure 11L). Taken together, BioNano spray not only modulates liver functioning but also regulates gut barrier integrity and inflammation by targeting localized persistent inflammation in the liver, driven by the hydrogel formulation and encapsulated exosomes. Therefore, by treating the master regulator (liver), it directly restored the gut‐liver axis‐associated complications. The reduction in ileal inflammatory cytokines and restoration of mucin and tight‐junction proteins in the treated rats indicate that healing the liver can break the vicious circle of gut leakiness and endotoxemia that fuels MAFLD. This finding supports bi‐directional gut‐liver crosstalk: improving liver inflammation likely reduced the systemic and portal inflammatory mediators that impair the gut barrier, thereby improving gut integrity, which in turn would limit further liver injury. Such positive feedback loops could partly explain the substantial improvement that we observed in both organs.

### 2.12 BioNano Spray Modulates the Dysbiotic Gut Microbiome Resulting in Gut Homeostasis

**FIGURE 12:**
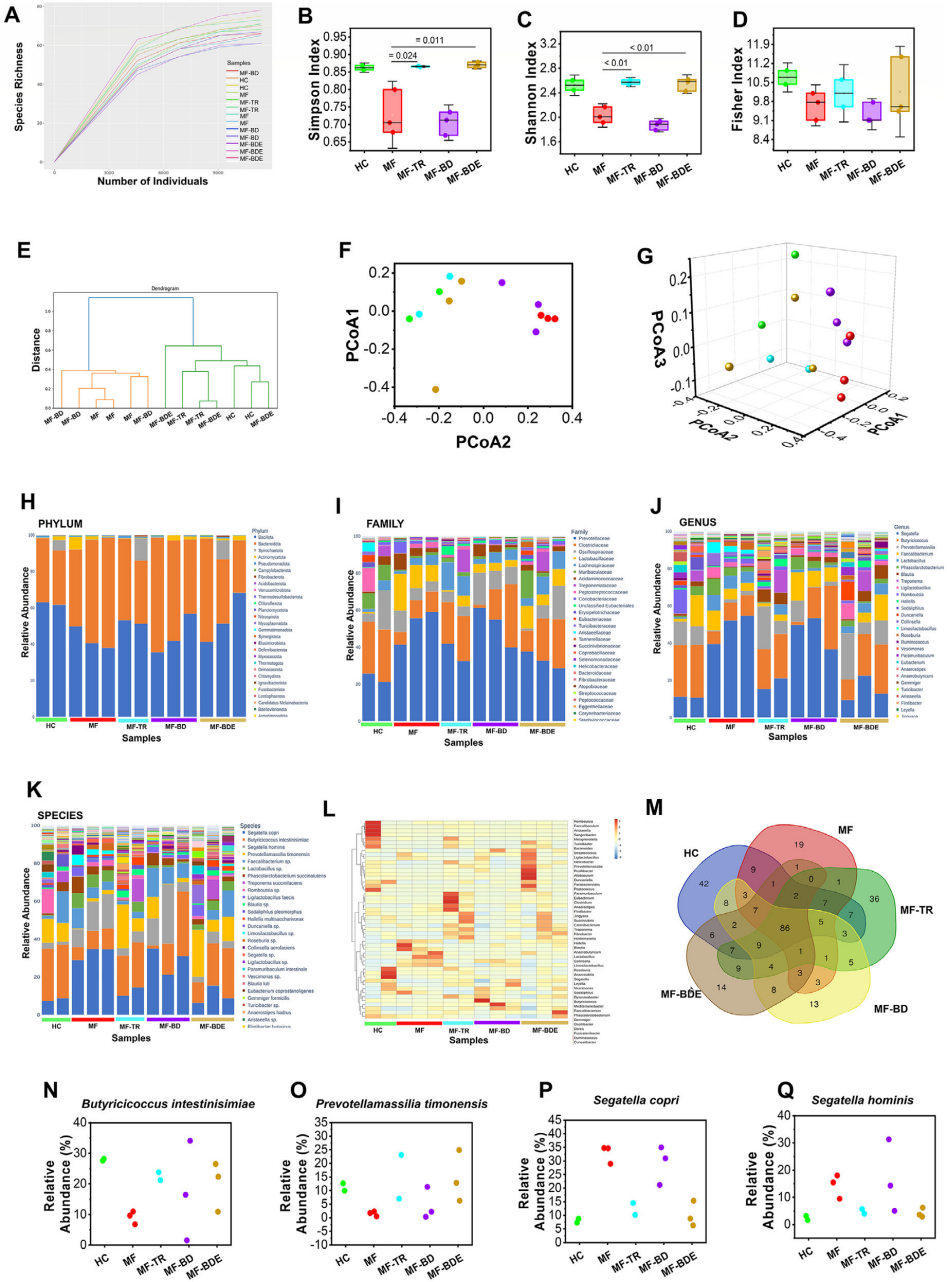
16S rRNA sequencing analysis of fecal samples to assess the gut microbiome remodeling. (A) Alpha diversity depicting the species richness graph of various groups; (B–D) Alpha diversity analysis profile expressed in Simpson index, Shannon index, and Fisher index, respectively; (E) Dendrogram representation depicting the similarity/dissimilarity of gut microbiome profile in the experimental groups; (F, G) Bray‐Curtis dissimilarity represented in 2D and 3D graphs; (H–K) Relative abundance variations in experimental groups at phylum, family, genus and species level; (L) Heatmap profile of gut microbiome analysis; (M) Venn diagram depicting the shared and unique microbiome population across the different groups; (N–Q) Changes in the relative abundance (%) of *Butyricicoccus intestinisimiae*, *Prevotellamassilia timonensis*, *Segatella copri*, and *Segatella hominis*, signifying the shift in microbiome content, n ≥ 2.

Modulating the gut‐liver axis, leading to microbiome remodeling, can be an emerging and comprehensive strategy for overall recovery from fatty liver‐related co‐morbidities [55, 56]. Studies of 16S rRNA sequencing revealed that BioNano spray treatment (MF‐BDE) significantly increased microbial species richness and diversity within the gut, comparable to that of healthy controls (Figure 12A). This was followed by significant restoration mediated by the lifestyle intervention group (MF‐TR). Specifically, alpha diversity, as depicted by the Simpson (SI) and Shannon index (SHI), was markedly (*p* < 0.01) elevated in the BioNano spray group (SI: 0.87 ± 0.007; SHI: 2.54 ± 0.1) and treadmill group (MF‐TR; SI: 0.86 ± 0.002, SHI: 2.57 ± 0.05) groups compared to the lower diversity in the negative control, MF(SI: 0.72 ± 0.063; SHI: 2.02 ± 0.12). This indicates a shift toward a more diverse and resilient microbial ecosystem. Such diversity is associated with improved metabolic and immunological functions, which are critical for intestinal health (Figure 12B–D). Moreover, dendrogram analysis revealed that the microbiome of the MF‐BDE group closely resembled that of healthy (Figure 12E). A similar trend was observed for beta diversity analysis, including PCoA1, PCoA2, and PCoA3 based on Bray‐Curtis dissimilarity, demonstrating distinct clustering between treated and untreated groups (with close clustering between healthy and BioNano spray‐treated group), indicating a treatment‐induced shift in the overall microbial composition (Figure 12F,G). Notably, the BioNano spray and treadmill exercise group clustered closely with the healthy group, suggesting a normalization of the microbiome and diversity. Taxonomic profiling revealed profound alterations in the gut microbiota of MAFLD animals, with consistent enrichment of pro‐inflammatory taxa and depletion of beneficial butyrate producers (Figure 12H–M and Figures S14 and S15). At the phylum level, MF group exhibited marked dysbiosis with reduced Bacillota (42.7 ± 6.19 vs. HC: 62.31 ± 1.03) and increase in Bacteroidota (53.19 ± 9.53 vs. HC: 32.49 ± 3.65) (Figure 12H). Prevotellaceae were enriched (51.97 ± 9.38 vs. HC: 23.42 ± 3.2), while Clostridiaceae declined (9.18 ± 2.18 vs. HC: 28.13 ± 0.23) (Figure 12I), suggesting controls, with a shorter distance, compared to the MF group which was farther apart (Figure 12E), suggesting disruption of short‐chain fatty acid (SCFA) balance and promotion of systemic inflammation. This is consistent with prior reports on detailed study of microbiota imbalance that heightens inflammation and SCFA imbalance [57, 58, 59]. At the species level, *Butyricicoccus intestinisimiae* (9.13 ± 2.14 vs. HC: 27.90 ± 0.43) and *Prevotellamassilia timonensis* (1.5 ± 0.92 vs. HC: 11.29 ± 1.89) were depleted, whereas *Segatella copri* (32.78 ± 3.32 vs. HC: 8.03 ± 1.04) and *Segatella hominis* (14.32 ± 4.4 vs. HC: 2.41 ± 1.09) increased (Figure 12N–Q), taxa associated with insulin resistance, inflammation, and lipid dysregulation [60, 61, 62, 63].

Post‐intervention, both the treadmill exercise group and BioNano spray‐treated group showed reversal of these shifts, with restored Bacillota and Bacteroidota populations, normalization of Prevotellaceae and Clostridiaceae, suppression of pathogenic *Segatella* sp., and recovery of butyrate producers, with BioNano spray‐treated group (MF‐BDE) exhibiting the most complete restoration (Figure 12M–Q).

These results indicate that therapeutic remodeling of the microbiota re‐establishes a SCFA‐enriched, anti‐inflammatory state that mitigates MAFLD progression. In this study, the idea of treating the main organ (liver) directly can help reduce co‐morbidities involving not just the adipose tissue, pancreas, but also the gut‐liver axis via the portal vein, are based on integrative correlations among hepatic recovery, reduced inflammatory burden, improved intestinal barrier integrity, and microbiome modulation, rather than direct mechanistic causation. In this context, a one‐time application of BioNano spray provides a next‐generation and efficient treatment alternative to traditional lifestyle changes (MF‐TR) and existing strategies.

### 2.13 Altered Transcriptomic and Proteomic Insights Mediated by BioNano Spray‐Induced Metabolic Reprogramming

**FIGURE 13:**
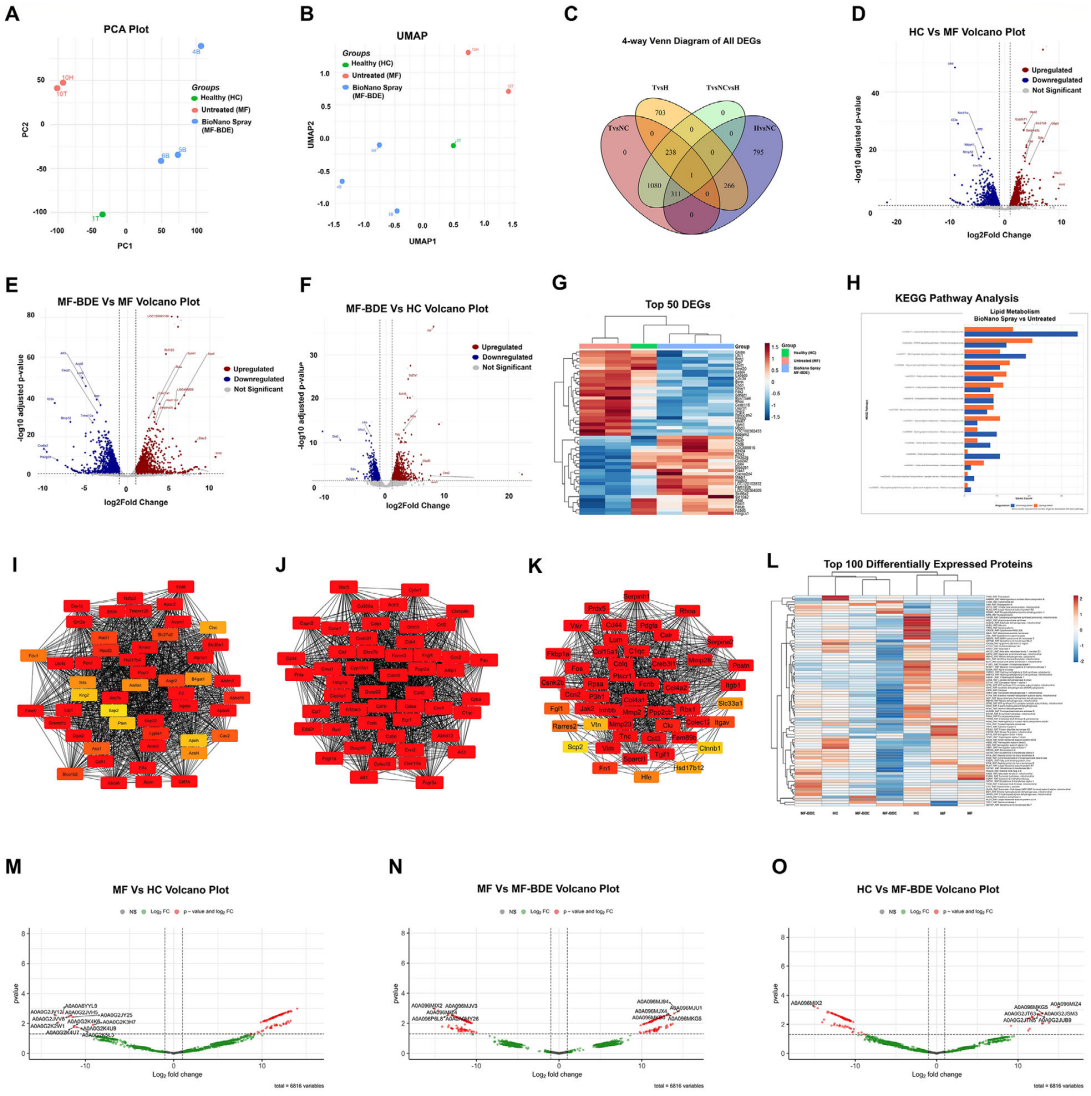
Multi‐omic profiling to assess the transcriptomic and proteomic changes post‐ therapeutic intervention. (A, B) PCA and UMAP plot from transcriptomic profiling depicts the clustering and variability among the experimental groups; (C) Venn diagram showing the different shared and unique genes expressed by the groups; (D–F) Volcano plots displaying the upregulated and downregulated genes between healthy vs. untreated, BioNano spray treated vs. untreated and BioNano spray vs. healthy respectively; (G) Heatmap profile of top 50 differentially expressed genes (DEGs) among the experimental groups; (H) KEGG analysis depicting the modulated pathways related to lipid metabolism in the treatment group; (I–K) Regulatory network analysis depicting the altered genes by our treatment approach to modulate lipid metabolism, inflammation, and fibrosis; (L) Heatmap profile of top 100 differentially expressed proteins from proteomic profiling; (M–O) Volcano plots displaying the upregulated and downregulated expression of proteins between untreated vs. healthy, negative vs. BioNano spray and healthy vs. BioNano spray experimental groups respectively.

The therapeutic intervention, with or without hUCMSCs‐Exo, broadly modulated core molecular pathways, restored multi‐organ function, and reshaped inter‐organ gene‐regulatory networks. To further delineate these mechanisms, transcriptomic and proteomic profiling of liver tissues was performed after 12 weeks of treatment. Principal component analysis (PCA) of the transcriptomic data revealed a clear separation between untreated animals (MF) and BioNano Spray‐treated group (MF‐BDE) along PC1, indicating substantial transcriptional divergence, while healthy controls (HC) clustered distinctly from both groups along PC1 and PC2 (Figure 13A). Tight clustering within each group suggested low technical variability. Uniform manifold approximation and projection (UMAP) analysis reinforced these trends, with the HC group positioned centrally, closer to MF‐BDE than MF, suggesting that the intervention partially restores, but does not fully recapitulate, the healthy transcriptional state (Figure 13B). Venn diagram further depicted the shared and unique transcripts among the experimental groups (Figure 13C). Volcano plots comparing healthy and negative controls highlighted strong upregulation of genes linked to glucose homeostasis (Sds), detoxification and lipid transport (Obp3), and amine metabolism (Inmt) in healthy animals, with downregulation in genes of stress and inflammatory mediators (Niban1, Atf3, Il23a, Mmp12) and a peroxisomal fatty acid transporter (Abcb1a) (Figure 13D). Comparison of BioNano Spray vs. untreated ones revealed pronounced induction of immune‐ and stress‐related genes (LOC120093108, Rnf125, Serce1, Aox4; all with ‐log_10_ p > 60), alongside suppression of inflammatory mediators (Atf3, Csrp2, Lcn2, Mmp12) (Figure 13E). Overlap in Atf3 and Mmp12 suppression across healthy vs. negative controls and BioNano spray vs. untreated group comparisons suggest remodeling of inflammatory pathways post‐intervention. Finally, BioNano Spray vs. healthy group comparisons revealed selective upregulation of recovery‐associated genes such as Stac3 (calcium signaling and mitochondrial activity, indicative of metabolic restoration) and Inmt (indoleamine detoxification, suggesting improved gut‐liver axis homeostasis), while genes related to oxidative phosphorylation (Cox6a2) and lipid mediator metabolism (Pla2g2d) were suppressed, in some cases below healthy baseline levels (Figure 13F). Together, these data, KEGG pathway analysis and GO, indicate that MF‐BDE treatment drives a complex transcriptional reprogramming, activating compensatory stress and immune pathways while concurrently silencing inflammatory and metabolic regulators (targeting metabolism of lipid, glucose, and insulin, alongside inducing matrix remodeling), ultimately creating a “quiescent” hepatic environment distinct from both untreated and healthy states (Figure 13H and Figures S16–S20). Similarly, heatmap and regulatory network analysis reveal similar observations of reprogramming the metabolic functioning and inflammatory signaling (Figure 13G,I–K).

To delve deeper into the differentially translated proteins post‐intervention, proteomic profiling revealed a cascade of differentially regulated proteins. Heatmap profiles and volcano plots depict the changes in untreated controls compared to healthy animals, displaying impaired detoxification and xenobiotic metabolism (reduced SULT1A1, AKR1D1), dysregulated amino acid and nitrogen metabolism (downregulated MAAI, ARG1, and CGL), loss of hepatoprotective signaling (low expression of CGL and ARG1; urea cycle dysfunction) and compromised adaptive immunity (suppressed IgG2B protein expression). Additionally, there was heightened mitochondrial stress and excess metabolic flux evident from upregulated proteins associated with fatty acid buffering (FABP1), gluconeogenesis (AATM), detoxification of reactive metabolites (COMT), stress defense (HSPA9), and redox shuttling (FH, MDHc) (Figure 13L–O). This pattern highlights the shift from a protective, detoxifying, and immune‐balanced liver toward a dysfunctional, inflamed, and metabolically unstable organ during MAFLD. In contrast, BioNano spray (MF‐BDE) treatment markedly reversed these alterations at the proteomic level, underscoring its therapeutic efficacy. Multi‐omic profiling revealed that MAFLD livers exhibited lipid and redox reprogramming, with increased FABP1, TCA/redox enzymes (FH, MDHc, GOT2), HSPA9, and detoxification mediators GSTs and COMT. Conversely, bile‐acid and xenobiotic metabolism (SULT1A1, AKR1D1), amino acid catabolism (MAAI, SDS), and urea‐cycle enzymes (ARG1, CGL, CTH) were suppressed. Stress sensors (ATF3, NIBAN1) and innate immune mediators (IL23A, MMP12) were elevated, while adaptive immune marker IgG2B declined.

Collectively, these signatures define a lipotoxic, redox‐stressed state prioritizing fatty acid oxidation and partial detoxification at the expense of nitrogen balance and immune regulation. MF‐BDE restored metabolic and immune homeostasis, likely through the combined anti‐inflammatory and pro‐regenerative actions of its bioactive hydrogel and exosomal miRNAs.

## 3 Conclusions

In this study, we have designed a ready‐to‐use bioadhesive spray formulation, with or without hUCMSCs‐Exo, as a localized, clinically relevant, amenable to minimally invasive therapy for progressive MAFLD involving persistent inflammation and liver fibrosis. This approach provided sustained exosome release directly at the target site, overcoming the short circulation time and rapid clearance associated with systemic administration. The therapeutic outcome (greater than conventional lifestyle intervention) was driven by the complementary effect of bioactive matrix (hyaluronic acid and polydopamine), exosomal proteins, and miRNAs, enabling modulation of multiple interconnected disease pathways mainly regulating inflammation and fatty acid trafficking. The BioNano spray‐based hydrogel formulation, once sprayed, remained adhered to the liver surface post‐covalent attachment mediated by exposure to 405 nm light. The approach is amenable to minimally invasive laparoscopic deployment, combined with biocompatibility, bioadhesion, immunomodulatory activity, and controlled release capability. Multi‐omic profiling (transcriptomic and proteomic studies) showed that MAFLD livers shift toward lipid metabolism dysregulation, with increased expression of FABP1, FH, and HSPA9, but lose detoxification and nitrogen balance, with reduced expression of SULT1A1 and ARG1, thereby driving inflammation via ATF3 and IL23A. Our intervention restored these disrupted pathways, promoting multi‐organ crosstalk, driving to mitigate hepatic fibrosis, correct disturbances in glucose and insulin homeostasis, mitigate gut inflammation, reinforce intestinal barrier integrity, modulate gut microbial composition, and promote recovery of disrupted hepatic microvasculature. Exercise and diet are known to improve MAFLD modestly, but rarely lead to fibrosis regression, whereas our intervention led to substantial reductions in fibrosis in 12 weeks.

Our study emphasizes the importance of an integrated, holistic, multi‐modal approach to address the complex inter‐organ communication driving MAFLD progression. Developed therapeutic intervention not only proved to be superior to the traditional strategy of lifestyle modification (daily exercise) but also demonstrated notable potential for attenuating inflammation, fibrosis, and lipid metabolism impairment, even without exosomes. Although we have shown correlations consistent with local hepatic treatment, macrophage polarization, and improved gut barrier function, direct causal relationships were not established in this study and will be addressed in future investigations. Proteomics showed many candidates, and future work could isolate the role of specific cargo (using loaded nanoparticles to mimic key exosomal signals). Future investigations will also focus on adapting and validating the delivery system for laparoscopic application to strengthen its clinical translatability. In summary, this study provides proof‐of‐concept evidence that a localized regenerative therapy can address the multifactorial pathology of MAFLD. By directly treating the liver (metabolic regulator), we alleviated systemic dysregulation. With further investigation at the cellular and functional level to identify the definitive roles, in vivo biodistribution and uptake of released exosomes, validation and safety profiling, the therapeutic strategy presented in this study could pave the way for new treatments for advanced liver fibrosis and metabolic disease.

## 4 Materials and Methodology

Detailed information on materials and methodology is provided in the Supporting Information.

### 4.1 Isolation, Characterization, and Proteomic Profiling of Isolated Exosomes

#### 4.1.1 Culturing of Human Umbilical Cord Mesenchymal Stem Cells and Isolation of Exosomes

Human umbilical cord‐derived mesenchymal stem cells (hUCMSCs, ILBS, Delhi) were expanded to passages 3–4 and cultured to 70%–80% confluency in α‐MEM (10% v/v FBS, 1% v/v antibiotic‐antimycotic). Medium was replaced with serum‐free medium (1% v/v antibiotic‐antimycotic), and cells were incubated for 48 h. Conditioned medium was sequentially centrifuged at 2000 g (10 min) and 16000 g (45 min) to remove debris and microvesicles, then concentrated using 100 kDa Amicon Ultra filters (Sigma–Aldrich) at 2000 g [64, 65].

#### 4.1.2 Protein Concentration Evaluation of Isolated Exosomes

Exosomal protein concentration was determined using the Pierce BCA Protein Assay Kit (Thermo Fisher Scientific) using the manufacturer's protocol. BSA standards were prepared for calibration, and 25 µL of each sample or standard was mixed with 200 µL BCA working reagent (50:1, Reagent A: B), incubated at 37°C for 30 min, and absorbance was measured at 562 nm in a 96‐well plate [65].

#### 4.1.3 Dynamic Light Scattering (DLS) Analysis of Extracted Exosomes

Hydrodynamic diameter and surface charge of exosomes were measured by dynamic light scattering (Malvern Zetasizer ZS90). Approximately 80 µg of exosomes were suspended in 1 mL Milli‐Q water, and measurements were performed in triplicate at 25°C [64].

#### 4.1.4 Nanoparticle Tracking Analysis (NTA) of Exosomes to Assess Size and Concentration

Particle size distribution and concentration of exosomes were assessed using a NanoSight NS300 (Malvern Panalytical, UK) with a 488 nm laser. Samples were analyzed in triplicate at camera level 14 and detection threshold 10. Each run comprised a 30 s video, processed using NTA software (v3.2) [64, 65, 66].

#### 4.1.5 Scanning Electron Microscopy (SEM) and Field Emission Scanning Electron Microscopy (FE‐SEM) of Isolated Exosomes

Exosome morphology and size were visualized by SEM (Zeiss EVO 18, Germany). Samples were fixed in 2% w/v paraformaldehyde at 4°C for 6 h, serially diluted (1:50–1:200) in filtered Milli‐Q water, drop‐cast onto plasma‐cleaned coverslips, air‐dried, and sputter‐coated with gold for 60 s. Imaging was performed at 10 kV, and particle dimensions were quantified using ImageJ. FE‐SEM imaging was conducted on 1:50 diluted samples prepared using the same protocol.

#### 4.1.6 Immunogold Transmission Electron Microscopy (TEM) Analysis of Exosomes to Assess Size and Presence of Exosome Surface Protein Markers

Exosomes were fixed in 2% w/v paraformaldehyde for 7 min at room temperature (RT), and 7 µL aliquots were adsorbed onto holey carbon grids (10 min, repeated thrice). Grids were rinsed with PBS, quenched with 50 mm glycine, permeabilized with 0.001% v/v Triton X‐100 (15 min), and blocked with 0.5% w/v BSA (5 min). Primary antibodies against CD9 and CD81 (1:20, Santa Cruz) in 0.5% w/v BSA were applied for 5 h, followed by gold‐conjugated secondary antibodies (1:20, Sigma–Aldrich) for 2 h. After PBS washes, grids were air‐dried and imaged using a TEM (FEI Tecnai G2 12 Twin, USA) at 120 kV.

#### 4.1.7 Proteomic Profiling and Protein Expression Analysis Isolated from hUCMSCs‐Exo

Proteomic analysis was performed on 10^10^ exosome particles (two replicates, E1 and E2) by Biokart India Pvt. Ltd. Exosome pellets were rinsed with MS‐grade water, lysed in three successive cycles, and proteins were precipitated overnight with acetone. The precipitates were recovered by centrifugation and resuspended in 50 mm ammonium bicarbonate with 0.1% w/v SDS. For each sample, 100 µg protein was reduced with 100 mm DTT (95°C, 1 h, 400 rpm), alkylated with 250 mm iodoacetamide (45 min, RT, dark), and digested overnight with trypsin (37°C). Peptides were vacuum‐dried, reconstituted in 0.1% v/v formic acid, desalted, and centrifuged (13 000 g). A 10 µL aliquot was injected onto a UPLC BEH C18 column (40°C; flow rate, 0.3 mL/min; Buffer A, water + 0.1% v/v formic acid; Buffer B, acetonitrile + 0.1% v/v formic acid). Separation was followed by MS and MS/MS acquisition on a Q‐TOF mass spectrometer (MassLynx 4.1, Waters). Peptide identification was performed using Progenesis software by matching MS/MS spectra to theoretical tryptic peptide databases. Gene Ontology (GO) enrichment was conducted to assign biological process, molecular function, and cellular component categories.

### 4.2 Development of Bioadhesive Sprayable Exosome‐Laden Hydrogel Formulations

#### 4.2.1 Synthesis of Methacrylated Gelatin and Methacrylated Hyaluronic Acid

Gelatin methacryloyl (GelMA) was synthesized following a modified standard protocol. Gelatin (10% w/v, Type A, porcine skin) was dissolved in Dulbecco's phosphate‐buffered saline (DPBS) under gentle heating, after which methacrylic anhydride (3 mL) was added dropwise with continuous stirring. The reaction proceeded for 1 h and was terminated by adding twice the initial reaction volume of DPBS. The product was then dialysed (12–14 kDa MWCO membrane) against distilled water for 5 days, lyophilized, and stored at 4°C until use [17]. Methacrylated hyaluronic acid (MeHA) was prepared with slight modifications to an established report. Hyaluronic acid (1% w/v) was dissolved in distilled water, and methacrylic anhydride (1 mL) was added dropwise under stirring. The pH was adjusted to 8–10, and the reaction was maintained overnight at 4°C. The product was dialyzed (12–14 kDa MWCO) for 3 days against distilled water, freeze‐dried, and stored at RT [17].

#### 4.2.2 Characterization of Synthesized Polymers by Spectroscopic Analysis

FTIR spectra details are provided in the Supporting Information. In brief, the spectra were recorded in the range of 500–4000 cm^−1^ using Bruker Tensor II, Bremen, Germany [67].

#### 4.2.3 Development of Smart Photocrosslinkable Bioadhesive Biospray and BioNano Spray Formulations

The developed photocrosslinkable sprayable Biospray formulations were composed of MeHA, GelMA, PEGDA, and dopamine, followed by the addition of photoinitiator LAP at a concentration of 3 mg/mL post completion of reaction. Three concentrations of dopamine were utilized to formulate sprayable formulations, namely 0.6% w/v (GPH‐0.6%D; P1), 1.2% w/v (GPH‐1.2%D; P2), and 1.8% w/v (GPH‐1.8%D; P3). The different concentrations were reacted with GelMA‐MeHA‐PEGDA (5% w/v GelMA, 1% w/v MeHA, and 1% v/v PEGDA) to initiate the polymerization and covalent attachment of dopamine in the presence of sodium hydroxide, and a strong oxidizing agent, sodium periodate (0.006% w/v). The finalized concentration of polymer formulation (P2) with exosome particles (10^10^ particles/mL) was termed BioNano Spray (bioadhesive sprayable nanotherapeutics spray), and without exosomes, P2 was named Biospray in this study.

#### 4.2.4 UV–vis Characterization of the Developed Sprayable Hydrogel Formulations

Ultraviolet–visible (UV–vis) spectra were acquired using a Varioskan Flash spectrophotometer (Thermo Fisher Scientific, USA) to monitor the incorporation of dopamine and it's in situ polymerization or oxidation to polydopamine within the GelMA/MeHA polymeric formulation. Sprayable formulations containing 0% (w/v) dopamine served as blanks. For each measurement, 200 µL of the sample was transferred to the designated measurement plate, and absorbance spectra (250–600 nm) were recorded at defined intervals over 48 h. To assess the conjugation of polydopamine to GelMA, formulations (P1, P2, and P3) were dialyzed (12–14 kDa MWCO membrane) against deionized water for 48 h to remove unbound small molecules. Dialyzed samples were centrifuged (3500 rpm, 5 min), and the supernatants were loaded into 96‐well plates for UV–vis analysis. Absorbance profiles were recorded from 0 min to 48 h to track peak shifts and oxidation kinetics of the incorporated polydopamine.

#### 4.2.5 Characterization of the Synthesized Polydopamine‐Conjugated Sprayable Bioadhesive Formulations by FTIR Spectroscopy

Lyophilized formulations containing varying concentrations of polydopamine were analyzed to confirm successful incorporation and in situ polymerization using FTIR spectroscopy. Spectra were recorded in the range of 500–4000 cm^−^^1^ with a Fourier transform infrared spectrometer (Bruker, Tensor II, Bremen, Germany).

#### 4.2.6 Rheological Characterization of Developed Photocrosslinkable Sprayable Formulations

In our study, hydrogel formulation (GPH, P1, P2, and P3) along with trehalose (2% w/v) and exosomes at a concentration of 10^10^ particles/mL, was used to develop the final bioactive formulations. Rheological properties of the different bioink formulations were evaluated using a rheometer (MCR 101, Anton Paar, Austria) to investigate their flow properties under various conditions. The analysis included: (i) Temperature Sweep: Measurements of complex viscosity were recorded over a temperature range of 10–50°C to assess thermally induced transitions.; (ii) Amplitude Sweep: To determine the linear viscoelastic region (LVER) and viscoelastic moduli, tests were performed at a constant angular frequency (ω = 10 rad/s) and varying shear strain (γ = 0.01–10000%) at 37°C ; (iii) Frequency Sweep: Conducted at a fixed shear strain of 10% with angular frequency ranging from 1 to 100 rad/s to assess the changes in storage and loss modulus; (iv) Photo‐Rheology: The influence of visible light exposure on the storage and loss modulus was monitored over time at 37°C to understand photo‐cross‐linking behavior of the bioinks upon exposure to 405 nm blue light.

#### 4.2.7 Lap Shear Test of the Sprayable Hydrogels

The adhesive strength of hydrogel layers formed on rat liver tissue was quantified using a lap shear test on an Instron universal testing machine (Instron, USA) equipped with a 50 N load cell. Liver specimens sprayed with BioNano spray were sandwiched between two acrylic sheets, maintaining a bonding region of 5 mm × 5 mm. An even layer of the test formulation was applied to both tissue surfaces, followed by placement of the acrylic sheets and photopolymerization under 405 nm light for 30, 60, or 90 s. This process yielded a thin hydrogel film at the tissue interface. Testing was conducted at RT with a constant crosshead speed of 1 mm/min. Load and extension were continuously recorded until complete bond failure. Each condition was evaluated in quintuplicate (*n* = 5) to ensure reproducibility.

#### 4.2.8 Force Mapping of the Photocrosslinkable Hydrogel Films by Atomic Force Microscopy (AFM) Analysis

Nanomechanical characterization of the samples was conducted using AFM force mapping. Samples were immobilized on pre‐cleaned glass coverslips by evenly spreading a thin layer of the test material, followed by air drying for 1–2 h to ensure firm adherence. Measurements were carried out on an Asylum Research MFP‐3D AFM system (Oxford Instruments, England) operating in contact force mapping mode. A soft silicon cantilever with a nominal spring constant of 1.86 nN/nm and an Amp InvOLS of 95.40 nm/V was employed. The spring constant was calibrated via the thermal noise method. Force mapping was performed over 20 × 20 µm regions, applying a maximum load of 700 nN. The force‐indentation data were analyzed using Johnson‐Kendall‐Roberts (JKR) fitting to determine both the sample modulus and reduced modulus. Each sample was assessed in at least three independent regions to ensure reproducibility. Data acquisition, processing, and curve fitting were performed using the manufacturer's proprietary software.

#### 4.2.9 Fabrication of off‐the‐shelf Bioactive Sprayable Bioadhesive Formulations

To develop an off‐the‐shelf lyophilized form of BioNano spray, the final formulation of P2 with exosomes at a concentration of 10^10^ particles/mL, trehalose (2% w/v), was mixed with 0.3% w/v LAP. Thereafter, the formulation was frozen at −80°C for 6 h, followed by lyophilization for 16 h to obtain a foam version of spray, which was stable at 4°C for at least 6 months. To further reverse into sprayable form, the lyophilized powder can be reconstituted in an appropriate volume of saline/PBS at 37°C–40°C for further therapeutic administration.

#### 4.2.10 Water Retention Ability of the Sprayable Polymer‐Based Hydrogels

Hydrogel formulations, with or without exosomes, were cast in rectangular PDMS molds and photo‐cross‐linked under 405 nm light for 60 s. Lyophilized samples (*n* = 5) were weighed (W_d_) and immersed in DPBS at RT. At predetermined intervals over 72 h, hydrogels were removed, gently blotted to remove excess PBS, weighed (W_s_), and returned to the medium. Water uptake was calculated for each time point. The swelling ratio (SR) was calculated using the equation:

$$\mathrm{Swelling}\;\mathrm{Ratio}\;=\frac{W_{s}-W_{d}}{W_{d}}$$

where, *W_s_* = weight at any given point, and *W_d_* = initial dry weight.

#### 4.2.11 In vitro Degradation Analysis in the Presence of PBS

Hydrogel stability was evaluated in DPBS at 37°C over 28 days. At defined intervals, samples were removed, the medium discarded, and hydrogels were freeze‐dried for 16 h. Residual dry weight (W_2_) was recorded and compared to the initial dry weight (W_1_) to calculate degradation (%) using

$$\text{Weight Loss}\;\left(\%\right)=\frac{W_{1}-W_{2}}{W_{1}}\times 100$$

W_1_ and W_2_ represent the weight of hydrogels at the initial time point and at any given time point, respectively.

#### 4.2.12 Exosome Release From BioNano Spray‐Based Hydrogel

To evaluate the sustained release profile of exosomes from sprayable hydrogels, exosomes were incorporated at a concentration of 10^10^ particles/mL into the prepolymer solution and cast into PDMS molds to form soft hydrogels via photo‐cross‐linking (exposure to 405 nm for 60 s). The formed hydrogels were incubated in 1 mL of PBS at 37°C, and the release profile was analyzed at predefined time points. Prior to release studies, hydrogels were frozen for 5–6 h and subsequently lyophilized for 16 h. At each sampling point, the PBS supernatant was collected and replenished with fresh PBS. The amount of exosome‐associated protein released over 28 days was quantified using a BCA protein assay kit (Pierce^TM^, Thermo Fisher Scientific).

#### 4.2.13 Exosome Internalization Studies in Hepatic Stellate Cells

Internalization of hydrogel‐released exosomes (RE) by LX‐2 cells was assessed using confocal microscopy. LX‐2 cells were cultured in DMEM/F12 with 4% v/v FBS and 1% v/v antibiotic‐antimycotic, seeded at 5 × 10^3^ cells per 35 mm confocal dish, and incubated for 24 h at 37°C. Exosomes were labeled with calcein‐AM (15 min), and unbound dye was removed via centrifugal filtration. RE (30 µg/mL) was added and incubated for 1 h and 3 h. Cells were fixed in 4% w/v paraformaldehyde and counterstained with DAPI. Imaging was performed on a Leica SP8 confocal microscope (Germany) to evaluate uptake and cell morphology.

#### 4.2.14 Cytocompatibility Assessment of the Developed Hydrogel Formulations

Biocompatibility of GPH, P1, P2, and P3 was evaluated using immortalized human hepatocytes (IHH) over five days. Cells (1 × 10^5^/well) were seeded, and viability was assessed on days 1, 2, and 5 via resazurin and MTT assays. For resazurin, constructs were incubated with Alamar Blue (8 µL/mL) for 3 h at 37°C, and fluorescence (Ex 540 nm/Em 600 nm) was measured. MTT assays followed standard protocols.

#### 4.2.15 Live‐Dead Assay

Treated 2D cultures were rinsed with PBS, incubated with calcein‐AM and PI in PBS for 30 min at 37°C per the manufacturer's instructions, washed thrice with PBS, and imaged under hydrated conditions using a confocal microscope (Leica SPII, Germany). Viable cells fluoresced green, while dead cells fluoresced red.

#### 4.2.16 Visualization of Cellular Morphology of the Growing Cells

F‐actin/DAPI staining was performed to assess tissue organization and cell morphology. Samples (days 2 and 5) were fixed in 4% w/v paraformaldehyde for 30 min, rinsed thrice with DPBS, permeabilized in 0.% v/v Triton X‐100 containing 1% w/v BSA, and stained with Alexa Fluor 488‐phalloidin (1:40) for 30 min at RT. Nuclei were counterstained with DAPI (1:500), and constructs were imaged using a confocal microscope (Leica SPII, Germany).

#### 4.2.17 Evaluation of Human Albumin Synthesis by In vitro Cultured Cells

Human albumin secretion by cells cultured in the presence of the hydrogel formulations (2D control, GPH, and P2) was quantified using an ELISA kit.On days 2 and 5, spent media from each sample were collected, and albumin levels were determined following the manufacturer's protocol.

#### 4.2.18 In vitro Immunocompatibility Assessment by Griess Assay

RAW 264.7 murine macrophages were maintained in high‐glucose DMEM supplemented with 10% v/v fetal bovine serum and 1% v/v antibiotic‐antimycotic solution at 37°C in 5% CO_2_. Nitric oxide production was assessed indirectly by quantifying nitrite (NO_2_^−1^) via the Griess‐Saville method. Cells were treated with different formulations, with or without hUCMSC‐derived exosomes, in the presence of LPS (1 µg/mL) for 24 h. Untreated cells served as negative controls and LPS‐only cells as positive controls. Post‐treatment, 100 µL of supernatant was mixed with 100 µL of freshly prepared Griess reagent (1% sulfanilamide and 0.1% N‐(1‐naphthyl)ethylenediamine dihydrochloride in 2.5% phosphoric acid) in a 96‐well plate. After 15 min at RT, absorbance was recorded at 540 nm, and nitrite concentrations were determined from a sodium nitrite standard curve.

### 4.3 Induction of MAFLD and Employed Therapeutic Intervention

#### 4.3.1 Generation of Chronic Metabolic Dysfunction‐Associated Fatty Liver Disease (MAFLD) in Rats

Male Sprague‐Dawley rats (*n* = 45; 4–6 weeks old, 150–180 g) were procured from CSIR‐CDRI, Lucknow, and housed under controlled laboratory conditions. Following a one‐week acclimatization, NAFLD was induced via oral gavage of a cholesterol‐rich emulsion diet (20 mL/kg/day) for 24 weeks, with modification from our previous report [17] (composition in Table S1). Control animals received an equal volume of saline. To exacerbate hepatic injury, CCl_4_ was administered intraperitoneally at a cumulative dose of 3 mL/kg, divided into three doses over one week. Rats had ad libitum access to standard chow, water, and 30% w/v sucrose solution throughout the study. Animals were randomly divided into equal numbers into five experimental groups (*n* = 9 each group), including negative (untreated MAFLD; MF) and healthy control (HC) animals. Overall, the five experimental groups were healthy control (HC), untreated chronic MAFLD (MF), MAFLD + treadmill exercise (MF‐TR), MAFLD + bioadhesive spray formulation devoid of exosomes (MF‐BD), and MAFLD + BioNano spray (spray containing exosomes; MF‐BDE). For the treadmill exercise group, the speed parameters were 8–18 m/min, with ramp time 300 s; at 10° inclination for 6 days/week with 20 min for first 3 weeks, 30 min for next 3 weeks, 45 min for last 6 weeks. Table S2 illustrates a detailed description of the number of animals used for different experimental evaluations.

#### 4.3.2 Minimally Invasive Surgical Intervention of the Chronic Fatty Liver Disease (MAFLD) Induced Animals

Animals from the respective experimental groups underwent surgical intervention on the right liver lobe. Briefly, anesthesia was induced and maintained with isoflurane (2%–4% v/v) delivered in 2% v/v medical‐grade oxygen. The abdominal region was shaved and disinfected with povidone‐iodine solution. The incision site was marked just below the sternum, running parallel to the ribcage. A midline laparotomy was performed, and the skin was gently separated from the underlying muscle layer. Thereafter, with the help of saline‐moistened cotton tips, the right liver lobe was exposed, and after spraying the formulation, the liver was exposed to 405 nm light for 60 s, to ensure cross‐linking and adherence onto liver tissue. Following the procedure, the incision was closed using 4‐0 vicryl sutures for the muscle layer and 5‐0 silk sutures for the skin, after which povidone‐iodine was applied over the surgical site. Postoperatively, animals received intramuscular ceftriaxone (40 mg/kg) for antibiotic prophylaxis and tramadol (5 mg/kg) for analgesia. They were placed on a heating pad and observed until full recovery from anesthesia.

### 4.4 Assessment of the Efficacy of our Therapeutic Approach to Alleviate Chronic MAFLD

#### 4.4.1 Dual X‐Ray Absorptiometry (DXA) and Lipid Profile Evaluation of the Animals

Body composition (fat mass %, lean mass %, and tissue fat %) was assessed using dual‐energy x‐ray absorptiometry (DXA; Medikors InAlyzer). DXA scans generated color‐coded images of tissue density (red: high‐fat, green: medium, blue: low). Animals were anesthetized with isoflurane (2%–4% v/v in 2% oxygen) during scanning.

Serum lipid profiling was performed to evaluate metabolic alterations. Fasting blood was collected from the retro‐orbital sinus at different time points. Total cholesterol, triglycerides, HDL, and LDL were measured using an automated biochemical analyzer, with LDL calculated via the Friedewald equation. Detailed methodology is provided in the methodology section.

#### 4.4.2 Biochemical Assessment of Liver Function Test After Minimally Invasive Surgical Intervention

Following the minimally invasive administration of the hydrogel formulations, blood samples were collected from the retro‐orbital sinus at days 0, 7, 28, 45, and 84, as previously described, under non‐fasting conditions. The pre‐surgical time point served as day 0. Serum obtained from these samples was analyzed for liver function markers using a biochemical analyzer (Erba CHEM‐7, Mannheim, Germany). Parameters measured included aspartate transaminase (SGOT/AST), alanine transaminase (SGPT/ALT), albumin, urea, and creatinine.

#### 4.4.3 Micro‐Computed Tomography (µCT) of Liver Tissues to Visualize the Microvasculature

Liver microvasculature was visualized using a silicone‐based contrast agent (Vascupaint, MediLumine^TM^) followed by µCT imaging. Under anesthesia heparin was injected intraperitoneally (6 mL/kg body weight), a thoracotomy was performed, and an 18G needle was inserted into the left ventricle with outflow enabled via the right atrium. The vasculature was first flushed with saline, then perfused with Vascupaint until complete filling was evident. Animals were stored at 4°C overnight for polymer curing, after which livers were excised, scanned by µCT, and reconstructed for 3D vascular analysis.

#### 4.4.4 Assessment of Glucose Homeostasis and Insulin Resistance

Perturbations in glucose regulation and insulin resistance, hallmark features of progressive MAFLD, were evaluated through fasting blood glucose (FBG), random blood glucose (RBG), glucose tolerance tests (GTT), serum insulin measurements, and HOMA assessments. FBG (post 12–16 h fasting) and RBG (afternoon) were recorded on days 0, 14, 28, 45, and 84 using the tail‐prick method with a glucometer. GTTs were performed on days 0, 28, 45, and 84 following a 12–16 h fast; animals received sucrose (1.5 g/kg, oral gavage), and blood glucose was measured at 0, 30, 60, 90, and 120 min. Delayed clearance indicated impaired glucose handling. Fasting serum insulin was quantified on days 0, 45, and 84, and insulin resistance/sensitivity indices were calculated using HOMA, QUICKI, and 1/insulin models to provide quantitative measures of metabolic dysfunction. The detailed formulas used for this experiment were:

HOMA−IR:[Insulin(mIU/mL)xFastingglucose(mg/dL)]/405

QUICKI:1/[loginsulin(mIU/mL)+logbaselineglucose(mg/dL)]

HOMA−IS:1/insulin(mIU/mL)

#### 4.4.5 Ultrasonographic Assessment of Liver Condition

Liver morphology was evaluated in control and experimental groups (*n* = 3 each) on day 0 and 12‐weeks post‐implantation. Animals were anesthetized with isoflurane (2%–4% v/v in 2% oxygen), shaved abdomen, and coupling gel applied before imaging. Scans were performed using a 7.5–10 MHz linear‐array transducer in B‐mode, with consistent gain and TGC settings (frequency: 4; FPS:4; power: 70%). Transverse and longitudinal views focused on hepatic tissue, assessing echogenicity, homogeneity, and structural heterogeneity. This non‐invasive approach enabled longitudinal monitoring and guided histological and molecular endpoint analyses.

#### 4.4.6 Animal Sacrifice and Histology and Immunohistochemistry (IHC) Analysis

At 12 weeks post‐intervention, treated liver lobes were excised, fixed in 4% w/v PFA overnight, processed for cryosectioning (10%–20% sucrose gradient), and prepared cryoblocks in OCT embedding medium. Sections (8–10 µm) were cut and mounted for staining. Histological evaluation included H&E, Oil Red O, and Picrosirius Red, with collagen I/III differentiation under polarized light. Further, histological images were quantified for steatosis and inflammation grade (Tables S3 and S4). For immunofluorescence, sections underwent antigen retrieval, permeabilization, and blocking, followed by overnight incubation with primary antibodies (8‐OHdG, α‐SMA, desmin, collagen I) and fluorescent secondary antibodies (Alexa Fluor 488/594). Images were captured using a Leica SP5 confocal microscope at 20x and 40x magnification.

#### 4.4.7 Gene Expression Analysis

After 12 weeks of intervention, animals were sacrificed, and the right liver lobe was excised, sectioned, snap‐frozen in liquid nitrogen, and stored at −80°C for molecular assays. The remaining liver tissue of the right lobe was processed for histopathological evaluation. Total RNA was extracted from frozen tissues using TRIzol reagent, quantified by NanoDrop, and reverse transcribed into cDNA. Gene expression profiling was performed by qRT‐PCR using primers for inflammatory, fibrotic, and metabolic markers (TNF‐α, TLR4, Caspase‐1, IL‐1β, IL‐18, PPARG, PPARA, SREBP‐1c, COL1A1, COL3A1, α‐SMA, TGF‐β1, iNOS, CD206, IL‐10, IL‐6, CD31, Ki67), with β‐actin as the reference. Relative expression was calculated using the 2–^ΔΔCt^ method and normalized to healthy controls [17]. The primer sequences are detailed in Tables S5 and S6.

#### 4.4.8 Protein‐Protein Interaction Network Analysis by STRING

Protein‐protein interaction (PPI) networks were constructed using STRING v12.0 (Search Tool for the Retrieval of Interacting Genes/Proteins), which integrates experimental evidence, computational predictions, co‐expression data, curated pathway resources, and literature mining. Target proteins implicated in metabolic dysregulation and fibrogenesis, PPARG, PPARA, SREBP‐1c, collagen I, collagen III, α‐SMA, and TGF‐β, were queried to generate high‐confidence interaction maps (Tables S8‐S9). Functional enrichment within STRING identified associated biological processes and signaling pathways. Network topologies and enrichment outputs were evaluated to delineate molecular interactions contributing to hepatic steatosis and fibrosis [68, 69, 70].

#### 4.4.9 Proteomic Analysis of Extracted Liver Tissue From Various Experimental Groups

Excised liver tissues were snap‐frozen (80°C) and processed in technical duplicates at Centyle Biotech Pvt. Ltd. Proteins were precipitated (TCA‐acetone, 4°C), resolved by SDS‐PAGE, and visualized with Coomassie stain. Selected bands were reduced (TCEP), alkylated (iodoacetamide), and digested with trypsin. Peptides were desalted, vacuum‐dried, reconstituted, and analyzed on an Agilent G6550B LC‐MS system coupled to a 1260 HPLC using a 60 min gradient on a C18 column. Further, detailed methodology is provided in the Supporting Information.

### 4.5 Statistical Analysis

All experiments were carried out in triplicate to ensure reproducibility. Data were expressed as mean ± standard deviation or mean ± standard error, as appropriate. Statistical comparisons among groups were conducted using one‐way or two‐way analysis of variance (ANOVA), followed by Tukey's honestly significant difference (HSD) post hoc test for multiple comparisons, with significance thresholds set at *p* < 0.05, *p* < 0.01, and *p* < 0.001. The analysis was performed using OriginPro 2021. The datasets exhibited approximately normal distributions with comparable variance across groups. Experiments were independently repeated multiple times, and the data shown represent the complete dataset from a single representative independent experiment. No samples were excluded from the analysis.

## Author Contributions

**Triya Saha**: conceptualization, data curation, formal analysis, methodology, validation, visualization, writing; original draft preparation, review, and editing. **Ayushi Mairal**: methodology and formal analysis. **Shreya Mehrotra**: methodology. **Shiv K. Sarin**: reviewing and editing the original manuscript. **Ashok Kumar**: conceptualization, investigation, project administration, funding acquisition, resources, validation, supervision, original draft review, and editing.

## Funding

This work was supported by the Science and Engineering Research Board (SERB) (IPA/2020/000026; SPR/2021/000544), the Indian Council of Medical Research (ICMR) (IIRPSG‐2024‐01‐06602; File No. 8/SG/Gastro/NM/DevRes/2024), the Ministry of Human Resource Development (MHRD), and the Indian Institute of Technology Kanpur.

## Ethics Statement

All animal studies were conducted in accordance with the guidelines of the Committee for the Control and Supervision of Experiments on Animals (CPCSEA), under the approval of the Institutional Animal Ethics Committee, Indian Institute of Technology Kanpur (Protocol No. IITK/IAEC/2022/1142 and IITK/IAEC/2025/1264). Animals were maintained under standard conditions with unrestricted access to pellet diet, water, and 20% w/v sucrose, and were cared for in compliance with institutional regulations. Human umbilical cord mesenchymal stem cells used in this study were gifted by the Institute of Liver and Biliary Sciences, Delhi (IEC/2022/92/MA02; ILBS). This study did not involve human participants, patients, or clinical data; therefore, patient or participant consent and clinical trial registration were not required.

## Conflicts of Interest

The authors declare no conflicts of interest.

## Supporting information




**Supporting File 1**: advs76420‐sup‐0001‐SuppMat.docx.

**Supporting File 2**: advs76420‐sup‐0002‐VideoS1.mp4.

**Supporting File 3**: advs76420‐sup‐0003‐VideoS2.mp4.

**Supporting File 4**: advs76420‐sup‐0004‐VideoS3.mp4.

**Supporting File 5**: advs76420‐sup‐0005‐VideoS4.mp4.

**Supporting File 6**: advs76420‐sup‐0006‐VideoS5.mp4.

**Supporting File 7**: advs76420‐sup‐0007‐VideoS6.mp4.

**Supporting File 8**: advs76420‐sup‐0008‐VideoS7.mp4.

**Supporting File 9**: advs76420‐sup‐0009‐VideoS8.mp4.

**Supporting File 10**: advs76420‐sup‐0010‐VideoS9.mp4.

## Data Availability

The data that support the findings of this study are available from the corresponding author upon reasonable request.
