# Supplementary material for: Bioengineered Exosome‐Loaded Immunomodulatory Bioadhesive Spray Reverses Liver Fibrosis and Metabolic Dysfunction That Triggers Beneficial Gut‐Liver Crosstalk in Chronic Fatty Liver Disease
Source: Adv Sci (Weinh). 2026 Jul 14:e76420. Online ahead of print. doi: 10.1002/advs.76420 (PMC13367107; doi:10.1002/advs.76420)
Supplement: Supplementary file 1 — Supporting File 1: advs76420‐sup‐0001‐SuppMat.docx. [file ADVS-9999-e76420-s002.docx]

**Supporting Information**

**Bioengineered Exosome-Loaded Immunomodulatory Bioadhesive Spray Reverses Liver Fibrosis and Metabolic Dysfunction That Triggers Beneficial Gut-Liver Crosstalk in Chronic Fatty Liver Disease**

Triya Saha^1^, Ayushi Mairal^1^, Shreya Mehrotra^1,2^, Shiv K. Sarin^3^, Ashok Kumar ^1,2,4,5,6^*

*^1^Department of Biological Sciences and Bioengineering; Indian Institute of Technology Kanpur, Kanpur, UP, India*

*^2^ Centre for Environmental Science and Engineering, Indian Institute of Technology Kanpur, Kanpur, UP, India*

*^3^ Department of Hepatology, Institute of Liver and Biliary Sciences, New Delhi, India*

*^4^ The Mehta Family Centre for Engineering in Medicine, Indian Institute of Technology Kanpur, Kanpur, UP, India*

*^5^ Centre for Nanosciences, Indian Institute of Technology Kanpur, Kanpur, UP, India*

*^6^ Centre of Excellence for Materials in Medicine, Gangwal School of Medical Sciences and Technology, Indian Institute of Technology Kanpur, Kanpur, UP, India*

*Correspondence should be addressed to

**Prof. Ashok Kumar**

Department of Biological Sciences and Bioengineering
Indian Institute of Technology Kanpur,
Kanpur-208016, UP, India
Email: [ashokkum@iitk.ac.in](mailto:ashokkum@iitk.ac.in)
Phone: +91-512-2594051

**1. Experimental Section**

**Materials**

Hyaluronic acid sodium salt (from Streptococcus equi), methacrylic anhydride, porcine skin Type A gelatin, poly(ethylene glycol) diacrylate (Mn ~250), dopamine hydrochloride, sodium periodate, Dulbecco’s Modified Eagle Medium (DMEM), α-Minimum Essential Medium (α-MEM), and diamidino-2-phenylindole dihydrochloride (DAPI) were obtained from Sigma-Aldrich (St. Louis, MO, USA). Vascupaint® silicone rubber injection compounds (lead-free) were sourced from MediLumine™. Gibco® (Thermo Fisher Scientific, USA) supplied fetal bovine serum (FBS), Insulin-Transferrin-Selenium supplement (ITS-G), GlutaMAX™, MEM non-essential amino acid solution (100X), and antibiotic-antimycotic solution (100X). The human serum albumin ELISA kit and monoclonal anti-collagen type I antibody were also purchased from Sigma-Aldrich. Primary antibodies against α-smooth muscle actin (α-SMA), and 8-hydroxy-2'-deoxyguanosine (8-OHdG) were procured from Santa Cruz Biotechnology. Additional antibodies, anti-desmin (rabbit), Alexa Fluor 488-conjugated phalloidin, goat anti-rabbit IgG H&L (Alexa Fluor 594), and goat anti-mouse IgG H&L (Alexa Fluor 488), were obtained from Abcam. The Pierce™ BCA protein assay kit was purchased from Thermo Fisher Scientific. Unless otherwise specified, all other chemicals and analytical-grade reagents were procured from Merck (Mumbai, India). Tissue culture-grade plasticware was obtained from Thermo Fisher Scientific. Immortalized human hepatocytes (IHH), human hepatic stellate cells (LX-2), and human umbilical cord-derived mesenchymal stem cells (hUCMSCs) were kindly provided by the Institute of Liver and Biliary Sciences (ILBS), New Delhi. Cells were cultured in α-MEM or DMEM/F12 supplemented with 10% (v/v) FBS and 1% (v/v) penicillin-streptomycin under standard conditions (37 ℃, 5% CO2, humidified atmosphere).

**1.1 Isolation, characterization, and sequencing of human umbilical cord mesenchymal stem cell-derived exosomes (hUCMSCs)**

*1.1.1 Culturing of human umbilical cord mesenchymal stem cells and isolation of exosomes*

Human umbilical cord-derived mesenchymal stem cells (hUCMSCs; obtained from ILBS, Delhi) were expanded up to passages 3-4 and cultured until 70-80% confluency. Upon reaching this stage, the complete growth medium (α-MEM supplemented with 10% v/v FBS and 1% v/v antibiotic-antimycotic) was changed with serum-free medium containing 1% v/v antibiotic-antimycotic, followed by incubation for 48 h. The conditioned medium was collected and sequentially centrifuged at 2000 g for 10 min to remove debris and at 16,000 g for 45 min to eliminate larger vesicles. The supernatant was subsequently concentrated using Amicon® Ultra centrifugal filters (100 kDa MWCO; Sigma-Aldrich) by repeated centrifugation at 2000 g [1-3].

*1.1.2 Protein concentration evaluation of isolated exosomes*

The protein concentration of the purified exosomes was quantified using the bicinchoninic acid (BCA) assay, following manufacturer’s instructions for the Pierce™ BCA Protein Assay Kit (Thermo Fisher Scientific). Standard curve was prepared by using different concentrations of bovine serum albumin (BSA). Briefly, 25 µL of each exosome sample or bovine serum albumin (BSA) standard was mixed with 200 µL of freshly prepared bicinchoninic acid (BCA) working reagent (Reagent A:Reagent B = 50:1) and incubated at 37 ℃ for 30 min. Post incubation, 200 µL of the reaction mixture was pipetted to a 96-well plate, and absorbance was recorded at 562 nm [2].

*1.1.3 Dynamic light scattering (DLS) analysis of exosomes*

The hydrodynamic size and surface charge (zeta potential) of isolated exosomes were analyzed using dynamic light scattering (DLS). Approximately 80 µg of exosomes were resuspended in 1 mL of Milli-Q water and assessed with a Malvern Zetasizer ZS90. Measurements of zeta potential, reflecting colloidal stability, were performed in triplicate at 25 ℃ [3].

*1.1.4 Nanoparticle tracking analysis (NTA) of exosomes to assess size and concentration*

The distribution of particle (exosomes) size and concentration were analyzed using a NanoSight NS300 system (Malvern Panalytical, UK) equipped with a 488 nm laser. Each sample was measured in triplicate at a camera level of 14 and a detection threshold of 10. For every run, 30 s videos were captured and analyzed using NTA software (v3.2) [3].

*1.1.5 Scanning Electron Microscopy and Field Emission Scanning Electron Microscopy (SEM) of isolated exosomes*

The morphology and approximate size of exosomes were examined using scanning electron microscopy (SEM). Samples were fixed in 2% (w/v) paraformaldehyde and incubated at 4 ℃ for 6 h, followed by serial dilutions (1:50, 1:100, and 1:200) in filtered Milli-Q water. Diluted samples were drop-cast onto plasma-cleaned coverslips, air-dried, and sputter-coated with gold for 60 s. Imaging was performed using a Zeiss EVO 18 SEM (Germany) at 10 kV accelerating voltage, and particle size analysis was carried out using ImageJ software. For FE-SEM imaging, exosomes (1:50 dilution) were prepared using the same fixation and drop-casting procedure as for SEM.

*1.1.6 Immunogold Transmission Electron Microscopy (TEM) analysis of exosomes to assess size and presence of exosome surface protein markers*

For immunogold labeling, exosome samples were firstly fixed in 2% (w/v) paraformaldehyde (PFA) for 7 min at room temperature (RT). A 7 µL aliquot of the fixed suspension was placed on clean parafilm, and holey carbon grids were floated on the drops for 10 min; this adsorption step was repeated three times. Grids were washed thrice with 1X PBS (5 min each) and then incubated in 50 mM glycine in PBS for 3 min to quench any residual fixative. Permeabilization was achieved by treating the grids with 0.001% (v/v) Triton X-100 for 15 min. Non-specific binding was minimized by blocking with 0.5% (w/v) BSA for 5 min at RT. The grids were then incubated for 5 h at RT with primary antibodies against CD9 and CD81 (1:20 dilution; Santa Cruz) prepared in 0.5% w/v BSA. Unbound antibodies were removed by washing three times with 1X Dulbecco’s PBS (DPBS) for 5 min each. A 2 h incubation with gold-conjugated secondary antibody at RT (1:20; Sigma-Aldrich) followed, after which grids were rinsed three times with 1X PBS, air-dried, and visualized using a transmission electron microscope (FEI Tecnai G2 12 Twin, USA) operating at 120 kV.

*1.1.7 Atomic Force Microscopy (AFM) analysis of isolated exosomes*

Nanomechanical characterization of the exosome samples was performed using AFM force mapping. Samples were drop-cast on pre-cleaned glass at various dilutions ranging between 1:50-1:200, then air drying for 1-2 h to ensure secure adherence. Measurements were taken on an Asylum Research MFP-3D AFM system (Oxford Instruments, England) operating in contact force mapping mode. A soft silicon cantilever with a nominal spring constant of 1.86 nN/nm and an Amp InvOLS of 95.40 nm/V was used. The spring constant was calibrated using the thermal noise method. Force mapping was performed over 20 x 20 µm regions, applying a maximum load of 700 nN. The force-indentation data were analyzed using Hertz fitting to determine both the sample modulus (Young’s modulus) and reduced modulus. Each sample was assessed in at least three independent regions to ensure reproducibility. Data acquisition, processing, and curve fitting were performed using the manufacturer’s proprietary software.

*1.1.8 Protein extraction and subsequent proteomic profiling of exosomes isolated from hUCMSCs*

Exosomal protein analysis was performed using electrospray ionization liquid chromatography-mass spectrometry (ESI LC-MS) with 10^10^ particles per sample, involving two experimental replicates (E1 and E2). The procedure was carried out by Biokart India Pvt. Ltd., specializing in next-generation sequencing and proteomics. After initial quality assessment, the exosome samples were centrifuged, and the pellets were rinsed with MS-grade water. Protein extraction was performed in three successive cycles using lysis buffers, and the combined supernatants underwent overnight acetone precipitation. The precipitated proteins were collected by centrifugation and dissolved in 50 mM ammonium bicarbonate containing 0.1% w/v SDS.

A total of 100 µg of protein was first reduced using 100 mM dithiothreitol (DTT) at 95 ℃ for 1 h (400 rpm), followed by alkylation with 250 mM iodoacetamide for 45 min at RT in the dark. The proteins were then enzymatically digested overnight with trypsin at 37 ℃. Resulting peptides were vacuum-dried, reconstituted in 50 µL of 0.1% formic acid (FA), and desalted. After centrifugation at 13,000 g, 10 µL of the peptide solution was loaded onto a C18 UPLC column for separation. Mass spectrometric analysis was performed using a Q-TOF instrument, with data acquired via MassLynx 4.1 (Waters). Peptides were identified by matching MS/MS spectra against theoretical tryptic peptide databases using Progenesis software (Waters).

***LC Parameters:***

*Column:* UPLC BEH C18 Column

*Solvent:* *Buffer A:* Water 0.1% FA; Buffer B: ACN 0.1% FA

*Flow Rate:* 0.3mL/min

*Sample Injection:* 10μL

*Column Oven Temp****:*** 40 ℃

Overall, a LC-MS/MS method was used for peptide profiling of the samples. The peptides generated by trypsin digestion of the sample were analyzed by QTOF MSMS. The raw data were searched against the database using analysis software, and the final results were analyzed accordingly. Based on the raw data, bioinformatic analysis was carried out to determine the biological functions, molecular functions, and cellular components (GO analysis).

**1. 2 Development of bioadhesive sprayable exosome-laden formulations**

*1.2.1 Synthesis of methacrylated gelatin (GelMA) and methacrylated hyaluronic acid (MeHA)*

GelMA was synthesized using a modified version of a standard protocol. Briefly, 10% w/v gelatin (Type A, porcine skin-derived) was dissolved in Dulbecco’s phosphate-buffered saline (DPBS) under gentle heating. Methacrylic anhydride (3 mL) was added slowly with continuous stirring, and the reaction was maintained for 1 h. The reaction was terminated by adding twice the initial volume of DPBS. The product was purified by dialysis using distilled water using a 12-14 kDa molecular weight cut-off membrane for 5 days, and then post-freezing was lyophilized for 48 h. The lyophilized GelMA was stored at 4 ℃ until further use.

MeHA was prepared following a slightly modified literature method. A 1% w/v hyaluronic acid solution was made in distilled water, and 1 mL of methacrylic anhydride was added dropwise with stirring. The pH of the solution was adjusted to 8-10, and the reaction was carried out overnight at 4 ℃. Purification was performed *via* dialysis for 3 days using a 12-14 kDa membrane, followed by freeze-drying. The obtained MeHA powder was stored at 4 ℃ until use.

*1.2.2 Characterization of synthesized polymers by spectroscopic analysis*

Fourier-transform infrared (FTIR) spectra were acquired in the 500-4000 cm^-1^ range using a Tensor II spectrometer (Bruker, Bremen, Germany) [4].

*1.2.3 Development of smart photocrosslinkable bioadhesive Biospray and BioNano spray formulations*

The developed photocrosslinkable sprayable Biospray formulations were composed of MeHA, GelMA, PEGDA, and dopamine, followed by dialysis of the formulation for 48 h using 12-14 kDa dialysis membrane. It was followed by the addition of photoinitiator LAP at a concentration of 3 mg/mL to form the final formulation. Three concentrations of dopamine were utilized to formulate sprayable formulations, namely 0.6 % w/v (GPH-0.6%D, P1), 1.2 % w/v (GPH-1.2%D, P2), and 1.8 % w/v (GPH-1.8%D, P3). The different concentrations were reacted with GelMA-MeHA (5% w/v GelMA, 1 % w/v MeHA, and 1% v/v PEGDA) to initiate the polymerization and covalent attachment of dopamine in the presence of sodium hydroxide, and a strong oxidizing agent, sodium periodate (0.006 % w/v). The finalized concentration of polymer formulation (P2) with exosome particles (10^10^ particles/mL) is termed BioNano Spray (bioadhesive sprayable nanotherapeutics spray), and without exosomes, P2 is named Biospray in this study.

*1.2.4 UV-Visible characterization of the developed sprayable hydrogel formulations*

UV-vis spectra were recorded using a UV-vis spectrophotometer (Varioskan® Flash, ThermoFisher, USA) at multiple time intervals to monitor dopamine incorporation and its in-situ polymerization and oxidation into polydopamine within the GelMA/MeHA polymeric system. Sprayable formulations without dopamine (0% w/v) served as blanks. For each reading, 200 µL of the sample was placed on the measurement surface, and absorbance was recorded over the UV-vis range for up to 48 h. To confirm the stable binding of polydopamine to GelMA, various formulations (P1, P2, and P3) were dialyzed against deionized water for 2 days using a 12-14 kDa membrane to remove unbound small molecules. The dialyzed samples were then centrifuged at 3500 rpm for 5 min, and the supernatant was transferred to a 96-well plate for UV-vis analysis (250-600 nm) at defined time points (0-48 h) to assess peak shifts and the progressive oxidation of dopamine to polydopamine.

*1.2.5 Characterization of the synthesized dopamine-conjugated sprayable bioadhesive formulations by FTIR spectroscopy*

Lyophilized formulations containing varying concentrations of polydopamine were analyzed to confirm successful incorporation and in-situ polymerization using FTIR spectroscopy. Spectra were recorded in the range of 500-4000 cm⁻¹ with a Fourier transform infrared spectrometer (Bruker, Tensor II, Bremen, Germany).

*1.2.6 Rheological characterization of developed photocrosslinkable sprayable formulations*

In our study, hydrogel formulation (GPH, P1, P2, and P3) along with trehalose (2% w/v) and exosomes at a concentration of 10^10^ particles/mL, was used to develop the final bioactive formulations. Rheological properties of the different bioink formulations were evaluated using a rheometer (MCR 101, Anton Paar, Austria) to investigate their flow properties under various conditions. The analysis included: (i) Temperature Sweep: Measurements of complex viscosity were recorded over a temperature range of 10-50 ℃ to assess thermally induced transitions; (ii) Amplitude Sweep: he linear viscoelastic region (LVER) and viscoelastic moduli were evaluated at a constant angular frequency of 10 rad/s while varying the shear strain from 0.01% to 10,000% at 37 ℃; (iii) Frequency Sweep: Measurements were carried out at a fixed shear strain of 10%, with the angular frequency varied between 1 and 100 rad/s to analyze changes in the storage and loss moduli; (iv) Photo-Rheology: The influence of visible light exposure on the storage and loss modulus was monitored over time at 37 ℃ to understand photo-crosslinking behavior of the bioinks upon exposure to 405 nm blue light.

*1.2.7 Lap Shear test of the sprayable hydrogels to assess the tissue adhesion*

The adhesive strength of the hydrogel layer adhered to rat liver tissue samples was evaluated using a lap shear test performed on an Instron universal testing machine equipped with a 50 N load cell. Briefly, liver tissue sprayed with BioNano spray was covered on both sides with acrylic plastic sheets. The bonding region between the substrates was maintained at an overlap area of 5 mm x 5 mm. It was followed by exposure to 405 nm light for different durations of 30 s, 60 s, and 90 s. Following exposure to the blue light, it led to formation of a thin hydrogel layer. The test was conducted at room temperature with a constant crosshead speed of 1 mm/min. The applied load and extension were recorded continuously until complete failure of the bond. All tests were conducted in n ≥ 4 to ensure reproducibility. Lap shear strength was calculated using the following formula:

Lap Shear Strength ($\tau)=$ F/A

where:

$\tau$= lap shear strength (MPa or N/mm²)

$F$= maximum load at failure (N) from the tensile testing machine

$A$= bonded overlap area = (bond width × overlap length) (mm²)

*1.2.8 Force mapping of the photocrosslinkable hydrogel films by Atomic Force Microscopy (AFM) analysis*

Nanomechanical characterization of the samples was performed using AFM force mapping. Samples were immobilized on pre-cleaned glass coverslips by spreading a thin layer of the test material evenly, then air drying for 1-2 h to ensure secure adherence. Measurements were taken on an Asylum Research MFP-3D AFM system (Oxford Instruments, England) operating in contact force mapping mode. A soft silicon cantilever with a nominal spring constant of 1.86 nN/nm and an Amp InvOLS of 95.40 nm/V was used. The spring constant was calibrated using the thermal noise method. Force mapping was performed over 20 x 20 µm regions, applying a maximum load of 700 nN. The force-indentation data were analyzed using Johnson-Kendall-Roberts (JKR) fitting to determine both the sample modulus and reduced modulus. Each sample was assessed in at least three independent regions to ensure reproducibility. Data acquisition, processing, and curve fitting were performed using the manufacturer’s proprietary software.

*1.2.9 Fabrication of off-the-shelf bioactive sprayable bioadhesive formulations*

To develop an off-the-shelf lyophilized form of BioNano spray, the final formulation of P2 with exosomes at a concentration of 10^10^ particles/mL, trehalose (2% w/v), was mixed with 0.3% w/v LAP. Thereafter, the formulation was frozen at -80 ℃ for 6 h, followed by process of lyophilization for 16 h to result in a foam version of spray, which is stable at 4 ℃ for at least 6 months. To further reverse into sprayable form, the lyophilized powder can be reconstituted in an appropriate volume of saline/PBS at 37-40 ℃ for further therapeutic administration.

*1.2.10 Water retention ability of the sprayable polymer-based hydrogels*

For swelling analysis, the final hydrogel formulations, with and without exosomes, were cast into rectangular PDMS molds and photo-crosslinked under 405 nm light for 60 s. The resulting hydrogels were lyophilized and subsequently immersed in Dulbecco’s phosphate-buffered saline (DPBS) to evaluate water uptake over 72 h. Prior to immersion, each lyophilized hydrogel (n = 5) was weighed (W₀). At designated time points, samples were removed, gently blotted with tissue to remove excess surface PBS, weighed (Wt), and returned to DPBS. This process was repeated for the full 72 h period.

The swelling ratio (SR) was calculated using the equation:

$$Swelling Ratio=\frac{W_{s}-W_{d}}{W_{d}}$$

Where, *W_s_*= weight at any given point, and *W_d_*= initial dry weight

*1.2.11 In vitro degradation analysis in the presence of PBS*

The stability of the hydrogels for a prolonged time period was assessed in Dulbecco’s phosphate-buffered saline (DPBS) over 28 days. Samples were incubated at 37 ℃, and at specified intervals, the surrounding DPBS was removed. The hydrogels were then freeze-dried for 16 h and weighed to determine the remaining mass. Degradation (%) was calculated by comparing the residual dry weight (W_2_) at each time point with the initial dry weight (W_1_) using the equation:

$$Weight Loss \left( \% \right)=\frac{W_{1}-W_{2}}{W_{1}} \times100$$

W_1_ and W_2_ represent the weight of the hydrogels at the initial time point and at any given time point, respectively.

*1.2.12 Exosome Release from BioNano spray-based hydrogel*

To evaluate the sustained release profile of exosomes from sprayable hydrogels, exosomes were incorporated at a concentration of 10^10^ particles/mL into the prepolymer solution and cast into PDMS molds to form soft hydrogels *via* photo-crosslinking (exposure to 405 nm for 60 s). The formed hydrogels were incubated in 1 mL of PBS at 37 ℃, and the release profile was analyzed at predefined time points. Prior to release studies, hydrogels were frozen for 5-6 hours and subsequently lyophilized for 16 hours. At each sampling point, the PBS supernatant was collected and replenished with fresh PBS. The amount of exosome-associated protein released over 28 days was quantified using a BCA protein assay kit (Pierce™, Thermo Fisher Scientific).

*1.2.13 Exosome internalization studies in hepatic stellate cells post-storage of lyophilized BioNano spray for 6 months*

The internalization of hydrogel-released exosomes (RE) by LX-2 cells was examined. Cells were maintained in DMEM/F12 medium supplemented with 4% v/v fetal bovine serum (FBS) and 1% v/v antibiotic-antimycotic solution. Lyophilized spray-foam constructs were stored at 4 ℃ for a maximum of six months before being used for release and *in vitro* assays.

For uptake experiments, LX-2 cells were seeded at a density of 5 x 10^3^ cells per 35 mm confocal dish and cultured for 24 h at 37 ℃ under humidified conditions. Exosomes were labeled with calcein-AM by incubating for 15 min, after which unbound dye was removed *via* centrifugal filtration. RE exosomes (30 µg/mL) were incubated for 1 h and 3 h. Following treatment, cells were fixed in 4% w/v paraformaldehyde, followed by counterstaining with DAPI to visualize nuclei. Confocal microscopy (Leica SP8, Germany) was used to evaluate exosome internalization and assess cellular morphology.

*1.2.14 Cytocompatibility assessment of the developed sprayable formulations with/without polydopamine conjugation*

The biocompatibility of GPH, P1, P2, and P3 formulations (200 µL/well) was assessed using immortalized human hepatocytes (IHH) over five days. Cells (1 x 10⁵/well) were seeded, and viability was evaluated on days 1, 2, and 5 using resazurin and MTT assays. For the resazurin assay, constructs were incubated with resazurin dye solution (8 µL/mL medium) for 3 h at 37 ℃, and fluorescence was measured (Ex 540 nm/Em 600 nm). Results were expressed as absolute values of fluorescence reading. MTT assays were performed according to standard procedures. Briefly, post incubation for 3 h, the formazan crystals were dissolved by DMSO and incubated for 20 min at RT before recording absorbance at 575 nm.

*1.2.15 Live-dead assay*

Cell viability was further assessed using a calcein-AM/propidium iodide (PI) live/dead assay. On day 5, treated 2D cultures were rinsed with sterile PBS and incubated with calcein-AM (green) and PI (red) in PBS for 30 min at 37 ℃, following the manufacturer’s protocol. After staining, samples were washed three times with PBS and imaged under hydrated conditions using a confocal microscope (Leica SPII, Germany). Viable cells emitted green fluorescence, whereas dead cells appeared red.

*1.2.16 Visualization of cellular morphology of the growing cells in the presence of the hydrogel formulations*

Tissue organization and cell morphology were assessed *via* F-actin/DAPI staining. Samples were fixed with 4% w/v paraformaldehyde for 30 min, rinsed three times with DPBS, and permeabilized in 0.1% v/v Triton X-100 with 1% w/v BSA. F-actin filaments were stained using Alexa Fluor 488-phalloidin (1:40 dilution) for 30 min at RT, while nuclei were counterstained with DAPI (1:500). Stained samples (days 2 and 5) were imaged using a confocal microscope (Leica SPII, Germany).

*1.2.17 Evaluation of human albumin synthesis by in-vitro cultured cells*

Human albumin secretion by cells cultured in the presence of the hydrogel formulations (2D control, GPH, and P2) was quantified using an ELISA kit (Sigma Aldrich, USA). On days 2 and 5, spent media from each sample was collected, and albumin levels were determined following the manufacturer’s protocol.

*1.2.18 In vitro immunocompatibility assessment by Griess assay*

RAW 264.7 murine macrophages were expanded in high-glucose DMEM in addition to 10% v/v fetal bovine serum and 1% v/v antibiotic-antimycotic solution and kept at 37 ℃ in a humidified 5% CO_2_ incubator. Nitric oxide (NO) production was indirectly quantified by measuring nitrite (NO_2_^-^) levels using the Griess-Saville assay. For the experiment, cells were treated with different formulations, with or without hUCMSCs-derived exosomes, in addition to lipopolysaccharide (LPS; 1 μg/mL) for 24 h. Untreated and LPS-only treated cells were considered as negative and positive controls, respectively. After incubation, 100 μL of supernatant from each well was mixed with an equal volume of freshly prepared Griess reagent (1% w/v sulfanilamide and 0.1% w/v N-(1-naphthyl)ethylenediamine dihydrochloride in 2.5% v/v phosphoric acid) in a 96-well plate. Following a 15 min incubation at RT for color development, absorbance was recorded at 540 nm using a microplate reader. A standard curve prepared from sodium nitrite solutions was used to determine the nitrite concentrations.

*1.2.19 Immunofluorescence staining of cultured murine macrophages*

RAW 264.7 murine macrophages (1 x 10⁵/well) were expanded in high-glucose DMEM in addition to 10% v/v fetal bovine serum and 1% v/v antibiotic-antimycotic solution and kept at 37 ℃ in a humidified 5% CO_2_ incubator. Post adherence after 24 h, different groups were considered: healthy controls, LPS treated (1 μg/mL), LPS with GPH (200 µL/well), LPS with GPH and 30 µg of exosomes, LPS with P2 (200 µL/well), LPS with P2 and 30 µg of exosomes. Post incubation for another 24 h, the cells were washed thrice with PBS followed by fixing the cells in 4% w/v filtered PFA for 30 min. Thereafter, permeabilization was carried out using 0.2% v/v Triton X-100 for 10 min, moving ahead by blocking of non-specific binding sites with a solution containing 1% w/v bovine serum albumin (BSA) and 10% v/v goat serum in PBST (PBS with 0.1% v/v Tween-20) for 1 h at RT. The fixed cells were thereafter incubated overnight at 4 ℃ with primary antibodies against CD163 (Abcam) and iNOS (Abcam). Following PBS washes, fluorescent secondary antibodies, Goat Anti-Rabbit IgG H&L (Alexa Fluor® 594; 1:200) and Goat Anti-Mouse IgG H&L (Alexa Fluor® 488; 1:200), were applied for 2 h at room temperature. Images were taken by a Leica SP5 confocal fluorescence microscope at 10x and 20x magnification.

**1.3 Development of the advanced stage of MAFLD rat model and employed therapeutic intervention**

*1.3.1 Generation of chronic metabolic dysfunction-associated fatty liver disease (MAFLD) in rats*

A total of 45 male Sprague-Dawley rats (4-6 weeks old, 150-180 g) were obtained from CSIR-CDRI, Lucknow, and housed under controlled laboratory conditions. Animals were allowed to acclimate for one week in compliance with institutional ethical standards. MAFLD was induced by administering a cholesterol-rich emulsion diet *via* oral gavage at 20 mL/kg body weight per day for 24 weeks. Control animals received an equal volume of saline. The composition of the MAFLD-inducing diet followed a previously validated protocol [3] and is provided in **Table S1**. After the dietary induction phase, animals were given intraperitoneal injections of carbon tetrachloride (CCl_4_) at a cumulative dose of 3 mL/kg body weight, divided into three doses over one week, to exacerbate hepatic inflammation, steatosis, and fibrosis. Throughout the study, rats had unrestricted access to standard chow, water, and a 30% w/v sucrose solution.

Animals were randomly divided in equal numbers into five experimental groups (n = 9, each group), including negative (untreated MAFLD; MF) and healthy control (HC) animals. Overall, the five experimental groups were healthy control (HC), chronic MAFLD (MF), MAFLD + treadmill exercise (MF-TR), MAFLD + bioadhesive spray formulation devoid of exosomes (MF-BD), and MAFLD + BioNano spray (containing exosomes; MF-BDE). **Table S2** illustrates a detailed description of the number of animals used for different experimental evaluations.

*1.3.2 Minimally invasive surgical intervention of the chronic fatty liver disease (MAFLD) induced animals*

Animals from the respective experimental groups underwent surgical intervention on the right liver lobe. Briefly, anesthesia was induced and maintained with isoflurane (2-4% v/v) delivered in 2% v/v medical-grade oxygen. The abdominal region was shaved and disinfected with povidone-iodine solution. The incision site was marked just below the sternum, running parallel to the ribcage. A midline laparotomy was performed, and the skin was gently separated from the muscle layer. Thereafter, saline-moistened cotton tips were used to expose the liver lobe, followed by spraying the bioadhesive formulations (1 mL of formulation containing 10^10^ exosomes; BioNano spray and only material; MF-BD), the liver was then exposed to 405 nm light for 60 s to ensure crosslinking and adherence to the liver tissue. Following the procedure, the incision was closed using 4-0 vicryl sutures for the muscle layer and 5-0 silk sutures for the skin, after which povidone-iodine was applied over the surgical site. Postoperatively, animals received intramuscular ceftriaxone (40 mg/kg) for antibiotic prophylaxis and tramadol (5 mg/kg) for analgesia. They were placed on a heating pad and observed until full recovery from anesthesia.

Post-surgical intervention, one animal group (n=9), devoid of any treatment, was administered for treadmill exercise daily for a period of 12 weeks before sacrifice. For the treadmill exercise group, the speed parameters were 8 m/min-18 m/min, with ramp time 300 sec; at 10º inclination for 6 days/week with 20 min for the first 3 weeks, 30 min for the next 3 weeks, 45 min for the last 6 weeks **(Video SV4)**.

**1.4 Assessing the therapeutic potential of the developed BioNano spray and Biospray to tackle the complex disease of MAFLD**

*1.4.1 Evaluating the fat content by Dual X-Ray Absorptiometry (DXA) and serum lipid profile*

To assess changes in fat composition following treatment, both qualitative and quantitative analyses were conducted. Body composition parameters, including fat mass (%), lean mass (%), and fat content in tissue (%), were measured using dual-energy X-ray absorptiometry (DXA; Medikors InAlyzer). DXA scans provided color-coded images indicating tissue density: red for high-density fat, green for medium-density fat, and blue for low-density fat. For imaging, animals were anesthetized with isoflurane (2-4% v/v) in 2% v/v medical-grade oxygen and positioned within the DXA chamber. In addition to body composition, bone mineral density (BMD) and bone mineral content (BMC) were recorded, although data are not presented due to the absence of significant changes. Given the strong metabolic component of MAFLD, lipid profile evaluation was also performed. Fasting blood samples were collected from the retro-orbital sinus on days 0, 14, 28 and 84. Serum levels of total cholesterol, triglycerides, HDL, and LDL were quantified using an automated biochemical analyzer. LDL was calculated according to the Friedewald equation [5, 6]:

Total LDL = Total cholesterol - Total HDL - (Triglycerides/5)

*1.4.2 Biochemical assessment of liver function test after sprayable hydrogel administration*

Following the sprayable administration of the bioadhesive formulations, retro-orbital route was utilized for blood collection on days 0, 7, 28, 45, and 84, under non-fasting conditions. The pre-surgical sample (day 0) was used as the baseline for comparison. Serum isolated from these samples was evaluated for liver function biomarkers using an automated biochemical analyzer (Erba CHEM-7, Mannheim, Germany). The assessed parameters included aspartate aminotransferase (AST/SGOT), alanine aminotransferase (ALT/SGPT), albumin, urea, and creatinine.

*1.4.3 Micro-Computed Tomography (µCT) of liver tissues to visualize the microvasculature*

To visualize the microvascular architecture of liver tissues from various experimental groups, a silicone-based contrast agent (Vascupaint) was employed for µCT imaging. Under anesthesia, a thoracotomy was performed to expose the heart and liver. Following careful dissection and rib-cage removal, a 18-G needle was inserted into the left ventricle, and the right atrium was incised to facilitate outflow of blood and infusates. Initially, saline was perfused through the heart to clear the vasculature, followed by administration of Vascupaint (as per the manufacturer's instructions). The infusion was continued until complete vascular perfusion was achieved, confirmed by the visual appearance of the contrast agent released through the aorta and observable staining of the vasculature. The animals were then stored at 4 ℃ overnight to allow curing and solidification of the injected silicone. Subsequently, liver tissues were carefully harvested and subjected to µCT scanning and 3D reconstruction for vascular analysis.

*1.4.4 Evaluating the insulin and glucose metabolism homeostasis post-therapeutic interventions*

Disruption of glucose homeostasis and the emergence of insulin resistance are well-established pathophysiological features of MAFLD, contributing significantly to its onset and progression. To monitor these alterations, fasting blood glucose (FBG) and random blood glucose (RBG) levels were measured on days 0, 14, 28, 45 and 84 using a glucometer (Glucocare™ Ultima, RMD Mediaids Limited, India) *via* the standard tail-prick method. In parallel, glucose tolerance tests (GTT) were conducted on days 0, 28, 45, and 84 to evaluate systemic glucose clearance dynamics.

For GTT, animals were subjected to fasting overnight (12-14 h), following which baseline FBG levels were recorded. A sucrose solution (1.5 g/kg body weight) was given using oral gavage, and subsequent blood glucose levels were measured at 30, 60, 90, and 120 min post-administration. In healthy animals, glucose levels typically return to baseline within 2 h or 120 min. However, in MAFLD models with impaired glucose and insulin regulation, this clearance is significantly delayed, making GTT a reliable indicator of metabolic dysfunction.

Additionally, fasting serum insulin levels were quantified biochemically at days 0, 45, and 84. Using the measured fasting glucose and insulin values, insulin resistance and sensitivity indices were computed employing the Homeostatic Model Assessment (HOMA), inverse insulin sensitivity (1/insulin), and Quantitative Insulin Sensitivity Check Index (QUICKI), as previously described [7, 8]. These indices provided quantitative insights into the degree of metabolic impairment associated with MAFLD progression.

The detailed formulas used for this experiment were:

HOMA-IR: [Insulin (mIU/mL) x Fasting glucose (mg/dL)]/405

QUICKI: 1/ [log insulin (mIU/mL) + log baseline glucose (mg/dL)]

HOMA-IS: 1/ insulin (mIU/mL)

*1.4.5 Ultrasonography of the experimental animals to assess the severity and extent of liver fibrosis at 12 weeks post-intervention*

Ultrasonographic imaging was conducted on both control and experimental animal groups (n = 3 per group) at 12 weeks post-implantation to evaluate liver morphology. Animals were anesthetized using 2-4% v/v isoflurane in 2% v/v oxygen, and abdominal fur was carefully removed to reduce imaging artifacts. A coupling gel was applied to the exposed area, followed by imaging using a multifrequency linear-array transducer (7.5-10 MHz). All scans were acquired in two-dimensional brightness mode (B-mode), with consistent gain and time gain compensation (TGC) settings (frequency: 4; FPS:40; power: 70%), maintained throughout the study to ensure reproducibility. Liver assessment was carried out using transverse and longitudinal scans with the focal zone centered on the hepatic tissue. Echotexture characteristics such as echogenicity, homogeneity, and presence of heterogeneity were systematically examined across all groups. This non-invasive imaging not only enabled monitoring of liver architecture but also helped to assess the optimal endpoint for subsequent histological and molecular analyses.

*1.4.6 Animal sacrifice, histological evaluation, and immunohistochemistry (IHC) analysis*

Post-intervention, animals were euthanized after 12 weeks, and the liver lobe treated with the hydrogel formulation was excised followed by overnight fixation at 4 ℃ in 4% w/v paraformaldehyde (PFA). Fixed tissues were cut into ~15 mm x 10 mm sections. Samples underwent sequential processing in a graded sucrose series (10%, 15%, and 20% w/v) before embedding in PolyFreeze cryomatrix (Sigma-Aldrich). Cryosections of 8-10 µm thickness were generated using a cryostat and mounted on poly-L-lysine-coated slides for subsequent analyses. Histological assessments included hematoxylin and eosin (H&E), Oil Red O (ORO), and Picrosirius Red staining. Additionally, immunofluorescent staining was conducted using primary antibodies against 8-hydroxy-2'-deoxyguanosine (8-OHdG), α-smooth muscle actin (α-SMA), desmin, and collagen I. To differentiate collagen subtypes, Picrosirius Red-stained sections were examined under polarized light using a Leica DM 2500 microscope, allowing visualization of collagen I and III fiber depositions.

For immunofluorescence staining, tissue cryosections were first rehydrated, rinsed with PBS, and subjected to heat-induced antigen retrieval in EDTA buffer at 95 ℃ for 20 min. Permeabilization was carried out using 0.2% v/v Triton X-100 for 10 min, moving ahead by blocking of non-specific binding sites with a solution containing 1% w/v bovine serum albumin (BSA) and 10% v/v goat serum in PBST (PBS with 0.1% v/v Tween-20) for 1 h at RT. The sections were thereafter incubated overnight at 4 ℃ with primary antibodies against 8-OHdG (1:200), α-SMA (1:50), desmin (1:200), collagen I (1:200). Following PBS washes, fluorescent secondary antibodies, Goat Anti-Rabbit IgG H&L (Alexa Fluor® 594; 1:200) and Goat Anti-Mouse IgG H&L (Alexa Fluor® 488; 1:200), were applied for 2 h at room temperature. Images were taken by a Leica SP5 confocal fluorescence microscope at 10x and 20x magnification.

*1.4.7 Gene Expression Analysis*

At 12 weeks post-implantation, animals were euthanized under deep anesthesia (2-4% v/v isoflurane in oxygen), followed by cervical dislocation. The right liver lobe was excised, sectioned into uniform blocks (6 mm x 6 mm), and snap-frozen in liquid nitrogen. The frozen samples were subsequently stored at -80 ℃ for downstream molecular analyses. Additional liver specimens containing scaffold remnants were preserved in fixative (4% w/v PFA for 16 h) and processed for histopathological examination.

To evaluate transcriptional responses, total RNA was extracted from liver tissues using TRI® Reagent (Sigma-Aldrich), following established phenol-chloroform phase separation protocols [2, 3]. Briefly, tissue samples from all experimental groups were homogenized in Trizol reagent, and RNA was isolated by organic extraction, precipitated, and rehydrated in nuclease-free diethyl pyrocarbonate (DEPC)-treated water. The concentration and purity of RNA were assessed using a NanoDrop spectrophotometer. Complementary DNA (cDNA) synthesis was performed using reverse transcriptase in a thermal cycler (Bio-Rad T100), utilizing the isolated RNA as the template. Quantitative real-time PCR (qRT-PCR) was then carried out using gene-specific primers (listed in **Table S6**) for assessment of pro-inflammatory, fibrotic, and metabolic markers, including TNF-α, TLR4, Caspase-1, IL-1β, IL-18, PPARG, PPARA, SREBP-1c, COL1A1, COL3A1, α-SMA, TGF-β1, iNOS, CD206, IL-10, IL-6, CD31, and Ki67, with β-actin serving as the internal reference gene. Thermal cycling parameters were set as follows: initial denaturation at 95 ℃ for 10 min, followed by 40 cycles of amplification (95 ℃ for 30 s, 54 ℃ for 60 s, 72 ℃ for 60 s), with a final dissociation step (95 ℃ for 60 s, 54 ℃ for 30 s, 95 ℃ for 30 s). Gene expression levels were assessed using the 2^–ΔΔCt^ method and normalized to healthy control (HC) samples, with results expressed as fold change.

*1.4.8 In silico gene and protein network analysis of differentially expressed genes*

GeneMANIA tool was utilized to identify additional gene targets influenced by the intervention. GeneMANIA (<http://www.genemania.org>) is an online gene function prediction platform that identifies functionally related genes by integrating extensive datasets encompassing co-expression, genetic interactions, protein-protein interactions, shared pathways, co-localization, and protein domain similarity [9].

Additionally, protein-protein interaction (PPI) networks were constructed using STRING v12.0 (Search Tool for the Retrieval of Interacting Genes/Proteins), which integrates experimental evidence, computational predictions, co-expression data, curated pathway resources, and literature mining. Target proteins implicated in metabolic dysregulation and fibrogenesis-PPARG, PPARA, SREBP-1c, collagen I, collagen III, α-SMA, and TGF-β, were queried to generate high-confidence interaction maps **(Tables S8-S9)**. Functional enrichment within STRING identified associated biological processes and signaling pathways. Network topologies and enrichment outputs were evaluated to delineate molecular interactions contributing to hepatic steatosis and fibrosis [9, 10].

*1.4.9 Proteomic analysis of extracted liver tissue from various experimental groups*

Following sacrifice, liver tissues were acquired, frozen in liquid nitrogen, and further stored at -80 ℃. Protein profiling (technical duplicates) was performed by Centyle Biotech Pvt. Ltd. Proteins were extracted from 80 µL of sample using 1 mL TCA-acetone (4 ℃, 2 h), pelleted, and resuspended in lysis buffer. Extracts were separated on SDS-PAGE (15% resolving, 4% stacking) at 50 mA until dye front migration was complete, stained with Coomassie Brilliant Blue, and imaged at 300 dpi. Selected gel bands were excised, reduced with 5 mM TCEP, alkylated with 50 mM iodoacetamide, and digested overnight with trypsin (1:50, 37 ℃). Peptides were desalted on C18 cartridges, vacuum-dried, and reconstituted in buffer A (5% acetonitrile, 0.1% formic acid).

Samples were centrifuged (8000 rpm, 5 min), and supernatants were analyzed on an Agilent G6550B LC-MS coupled to an Agilent 1260 Infinity HPLC. Approximately 1 µg of peptide was loaded onto a 50 cm C18 Easy-Spray column (3.0 μm) and eluted with a 60 min linear gradient of buffer B (80% acetonitrile, 0.1% formic acid) at 300 nL/min. MS1 spectra were acquired at 70,000 resolution and MS2 at 17,500 with 10 s dynamic exclusion.

***Data Processing***

The raw mass spectrometry data (.d files) were analyzed using the Comet search engine within the Trans-Proteomic Pipeline, referencing the Phaeodactylum tricornutum proteome database (UniProt ID: UP000000759). The parameters for searching were set to a precursor mass tolerance of ± 20 ppm and a fragment mass tolerance of 0.02 Da. Additionally; trypsin was used as the digestion enzyme. Cysteine residues carbamidomethylation was used as a fixed modification, whereas methionine oxidation and N-terminal acetylation were considered to be modifications.


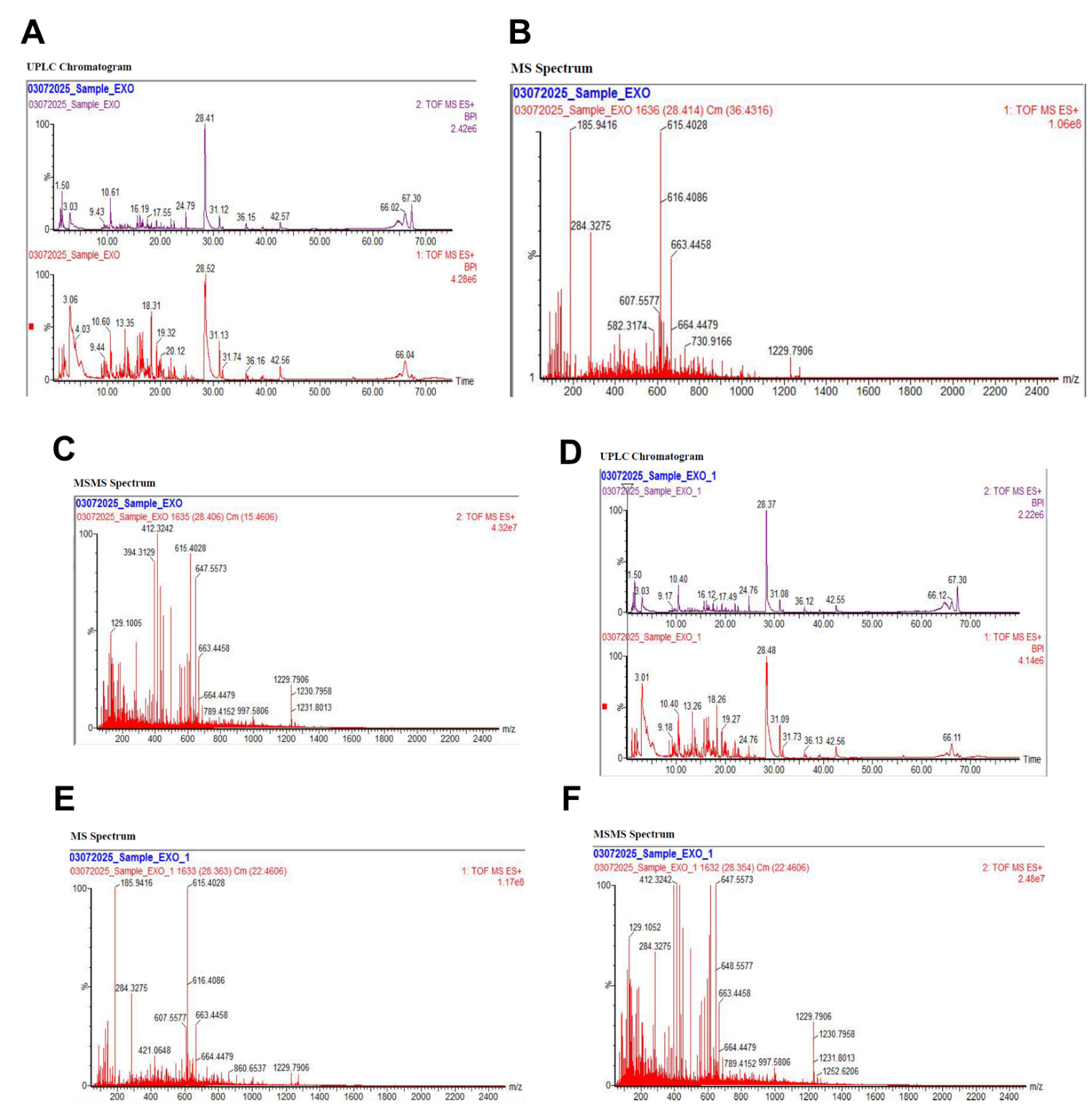


***Figure S1. MS Spectra of exosomes in technical duplicates (E1 and E2). (A)*** *UPLC chromatogram obtained from exosome proteomic profiling (E1);* ***(B-C)*** *MS and MSMS spectrum graphs (E1);* ***(D)*** *UPLC chromatogram of E2;* ***(E-F)*** *MS and MSMS spectrum graphs of E2.*


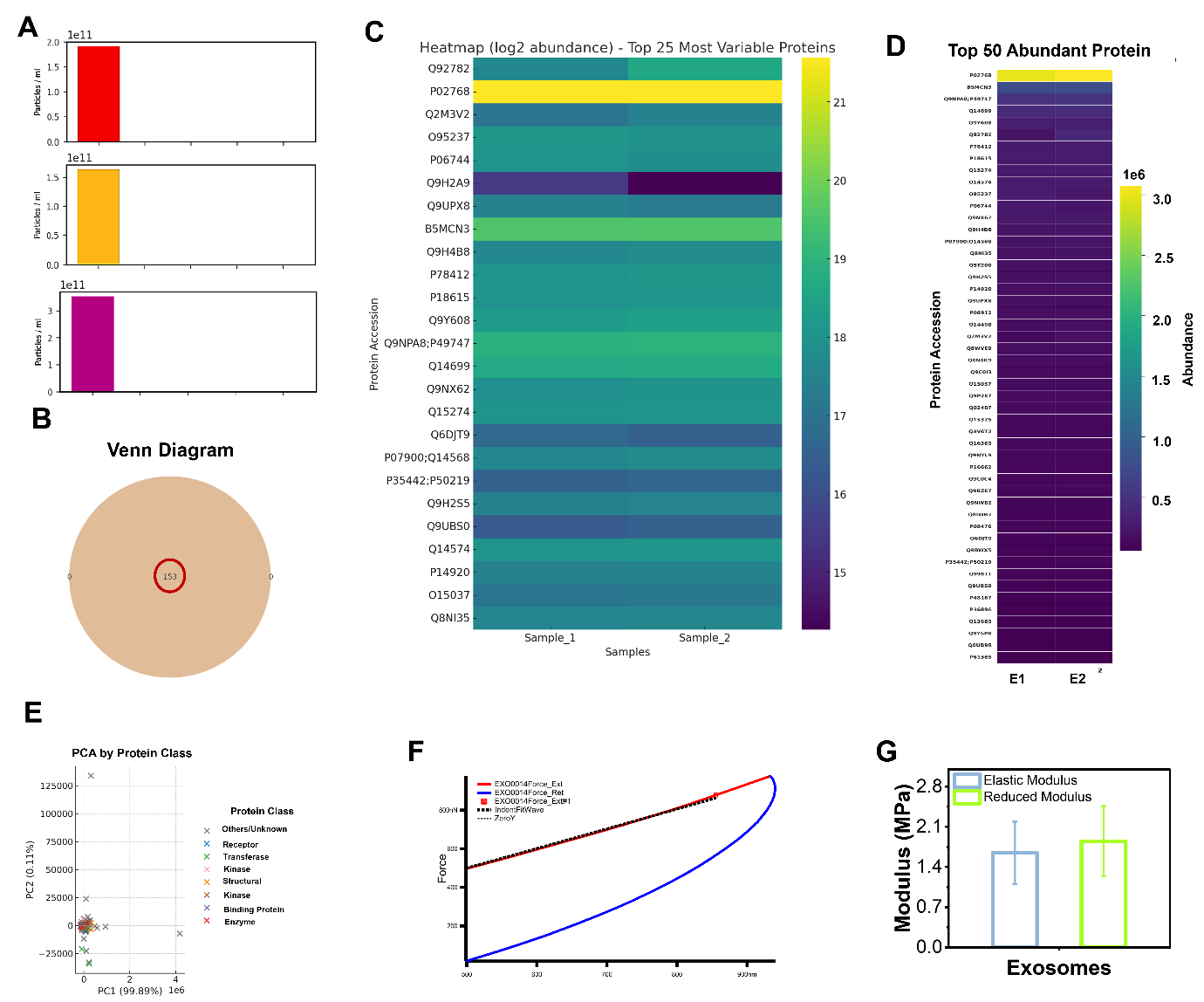


***Figure S2. Nanoparticle tracking analysis, proteomic profiling and force mapping of hUCMSCs-Exo. (A)*** *Nanoparticle tracking analysis showing the particle count per mL of exosomes, n=3;* ***(B)*** *Venn diagram representing the number of common proteins between technical duplicates (E1 and E2);* ***(C)*** *Heatmap profile of the top 25 variable proteins detected;* ***(D)*** *List of top 50 abundant proteins in isolated exosomes;* ***(E)*** *PCA plot by protein class;* ***(F)*** *Force vs Indent graph demonstrating the bioadhesive nature of the isolated exosomes, n=3;* ***(G)*** *Force mapping by AFM revealing the Young’s (elastic) and reduced modulus of 1.65 ± 0.54 MPa and 1.85 ± 0.61 MPa, respectively representing the intact nature and enriched with dense amount of bioactives.. Data is expressed as Mean ± S.D., n=3.*


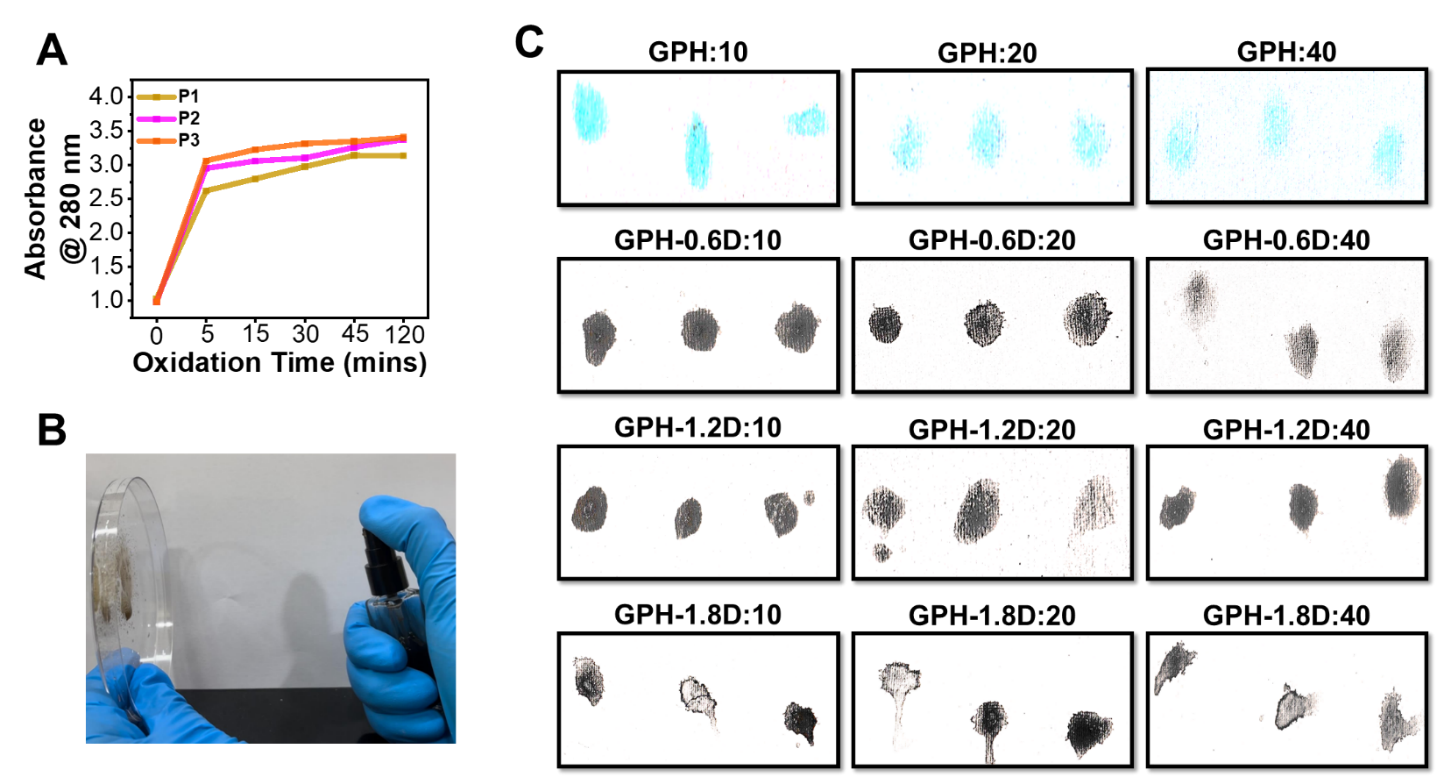


***Figure S3. Characterization of dopamine-conjugated sprayable formulations and assessing the ability to be sprayable. (A)*** *Oxidation time required to initiate non-covalent attachment of dopamine and conversion to poly-dopamine showing increasing absorbance at 280 nm after 120 min of reaction initiation signifying dopamine oxidation and polydopamine conjugation to the GelMA/MeHA blend; n=3;* ***(B)*** *Digital image depicting the ability to be sprayable;* ***(C)*** *Sprayability assessment of different formulations from distances of 10, 20, and 40 cm from the sprayed object, n=3.*


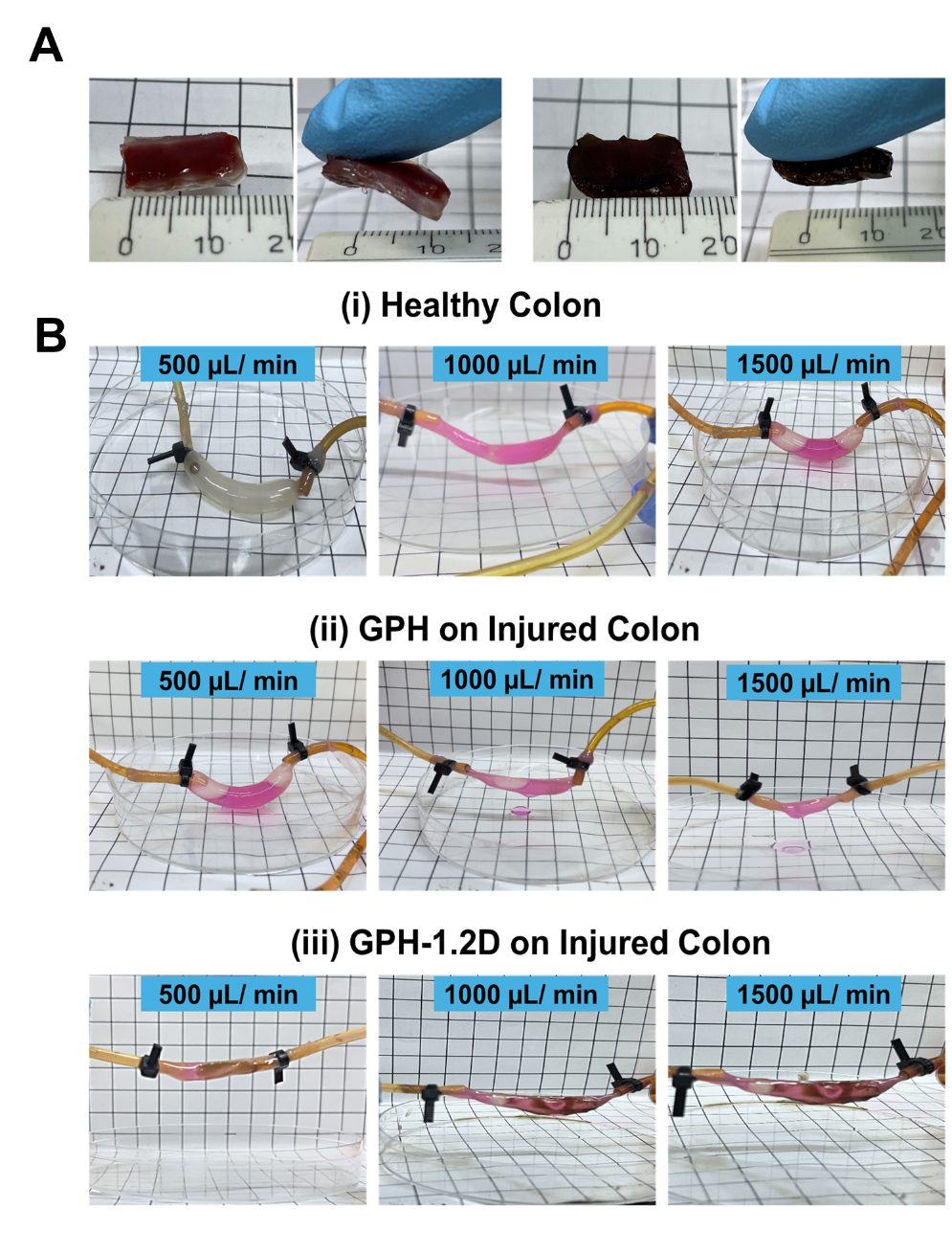


***Figure S4. Burst pressure test assessment of the sealant nature of the developed formulations. (A)*** *Greater bioadhesivity displayed by the polydopamine-conjugated hydrogel on rat liver tissue compared to GPH;* ***(B)*** *Burst pressure test assessment with GPH and chosen bioadhesive formulation; P2 (GPH-1.2D) on rat colon tissue.*

***Figure S5. Cytocompatibility and hemolysis assessment of the developed formulations. (A)*** *Live/dead assay of the treated IHH cells on day 5, n=4;* ***(B)*** *F-actin/DAPI visualization staining of the IHH cells in response to the formulations on days 2 and 5, n=4;* ***(C-F)*** *Digital images illustrating the hemolysis in the positive control (Triton X) and no hemolysis in GPH, P1, P2, and P3, respectively, n=2, comparable to the negative controls (PBS);* ***(G)*** *Albumin ELISA on days 2 and 5, Data is expressed as Mean ± S.D, n=2;****(H)*** *Degradation ability of the hydrogels in PBS over 28 days. . Data is expressed as Mean ± S.D, n=3.*

***
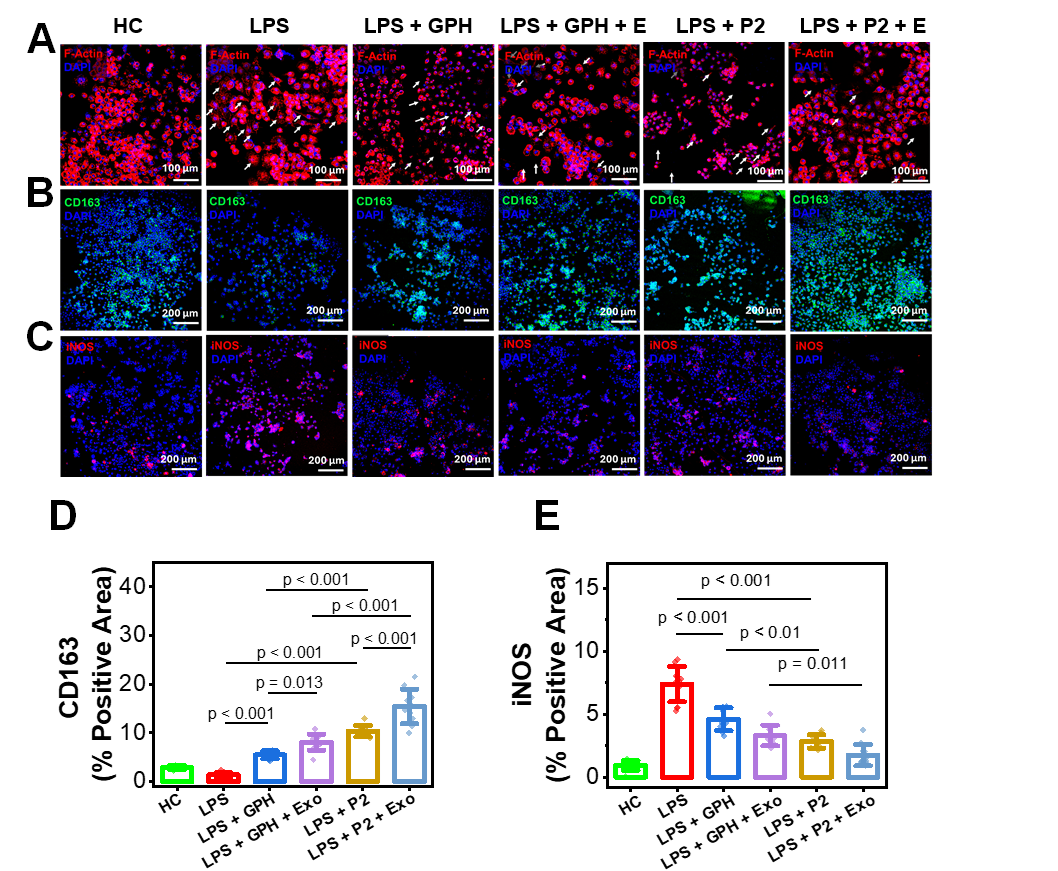
***

***Figure S6. Cytocompatibility and immunofluorescence assessment of macrophage polarization ability by the developed formulation with/without exosomes. (A)*** *F-actin/DAPI visualization of the RAW 264.7 cells when treated with GPH and P2 in the presence/absence of hUCMSCs-Exo;* ***(B)*** *Immuno-fluorescence assessment of the cells for distinctive marker of M2 phenotype (CD163);* ***(C)*** *Immuno-fluorescence evaluation of the characteristic marker of stress and M1 phenotype (iNOS), n=4;* ***(D)*** *CD163 staining quantification;* ***(E)*** *iNOS staining quantification. Data is expressed as Mean ± S.D., n≥9.*

***Figure S7. In vivo degradation assessment of the sprayed bioadhesive hydrogel over 30 days. (A)*** *Digital image representing sprayed hydrogel after administration and photocrosslinking;* ***(B)*** *Minimal presence/biodegradation of sprayed hydrogel after 30 days of administration.*


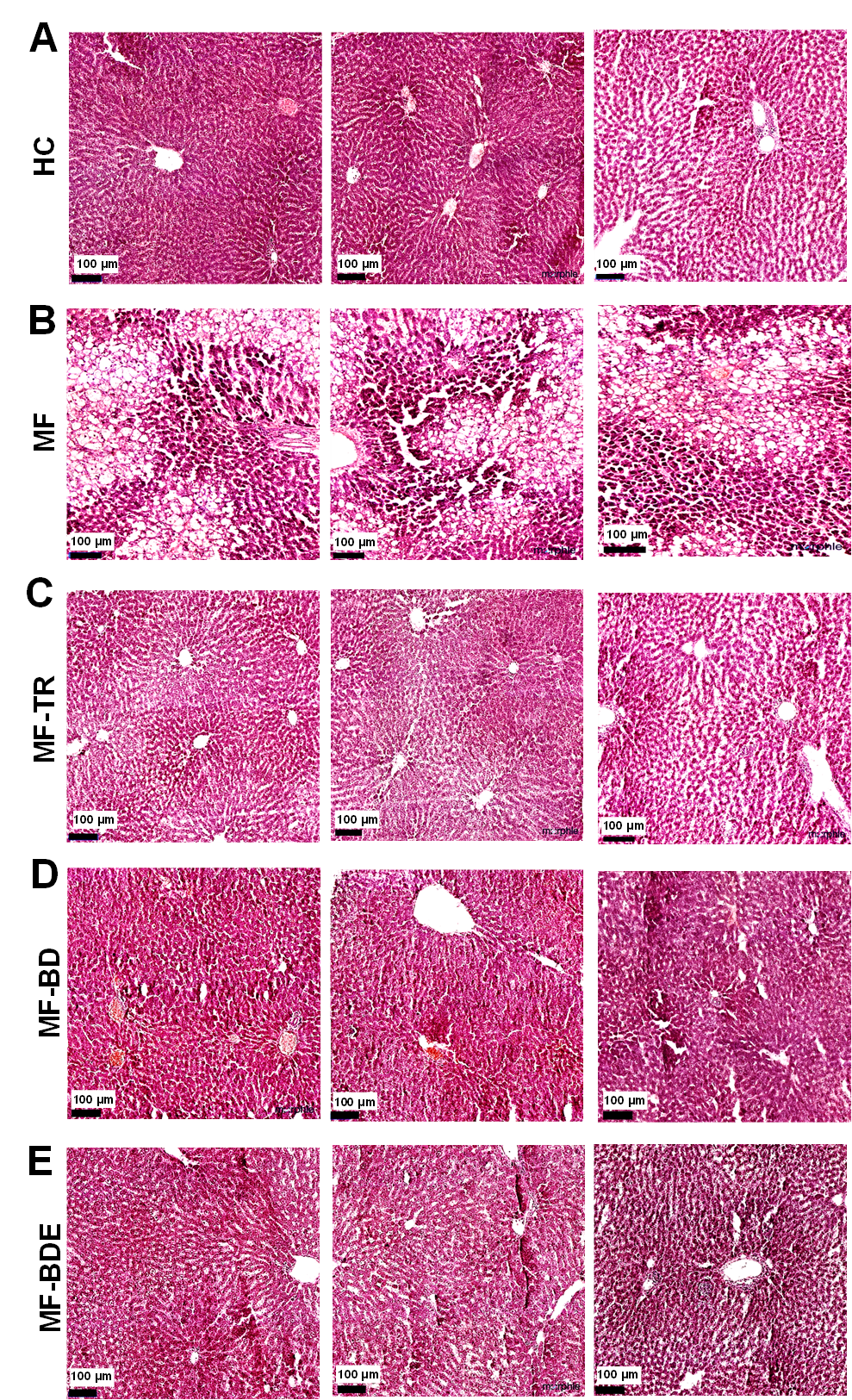


***Figure S8. Histopathological assessment to evaluate changes in liver microarchitecture across different liver lobes. (A-E)*** *Hematoxylin and eosin (H&E) staining of the liver tissues of various experimental groups of healthy (HC), untreated MAFLD (MF), treadmill exercise (MF-TR), only hydrogel spray (biospray; MF-BD) and BioNano spray-treated (MF-BDE) groups, respectively.*

*
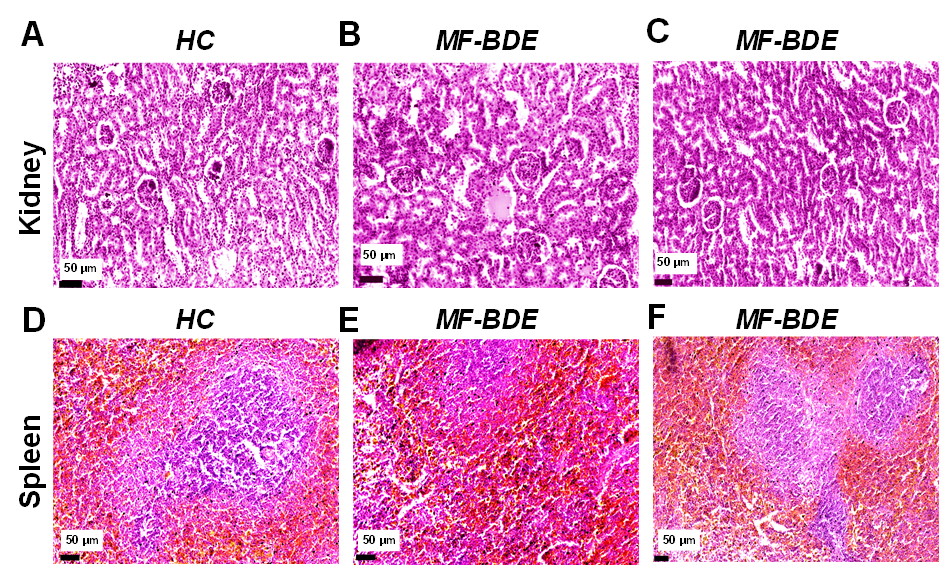
*

***Figure S9. Long-term in in-vivo biocompatibility assessment of BioNano spray (MF-BDE) treatment. (A)*** *Hematoxylin and eosin (H&E) staining assessment of healthy rat kidney;* ***(B-C)*** *Histopathological evaluation of BioNano spray-treated animals (MF-BDE) after 2 weeks and 12 weeks of administration, respectively;* ***(D)*** *Histological evaluation of healthy rat spleen as control;* ***(E-F)*** *H&E imaging of rat spleen after 2 weeks and 12 weeks of BioNano spray administration, respectively.*


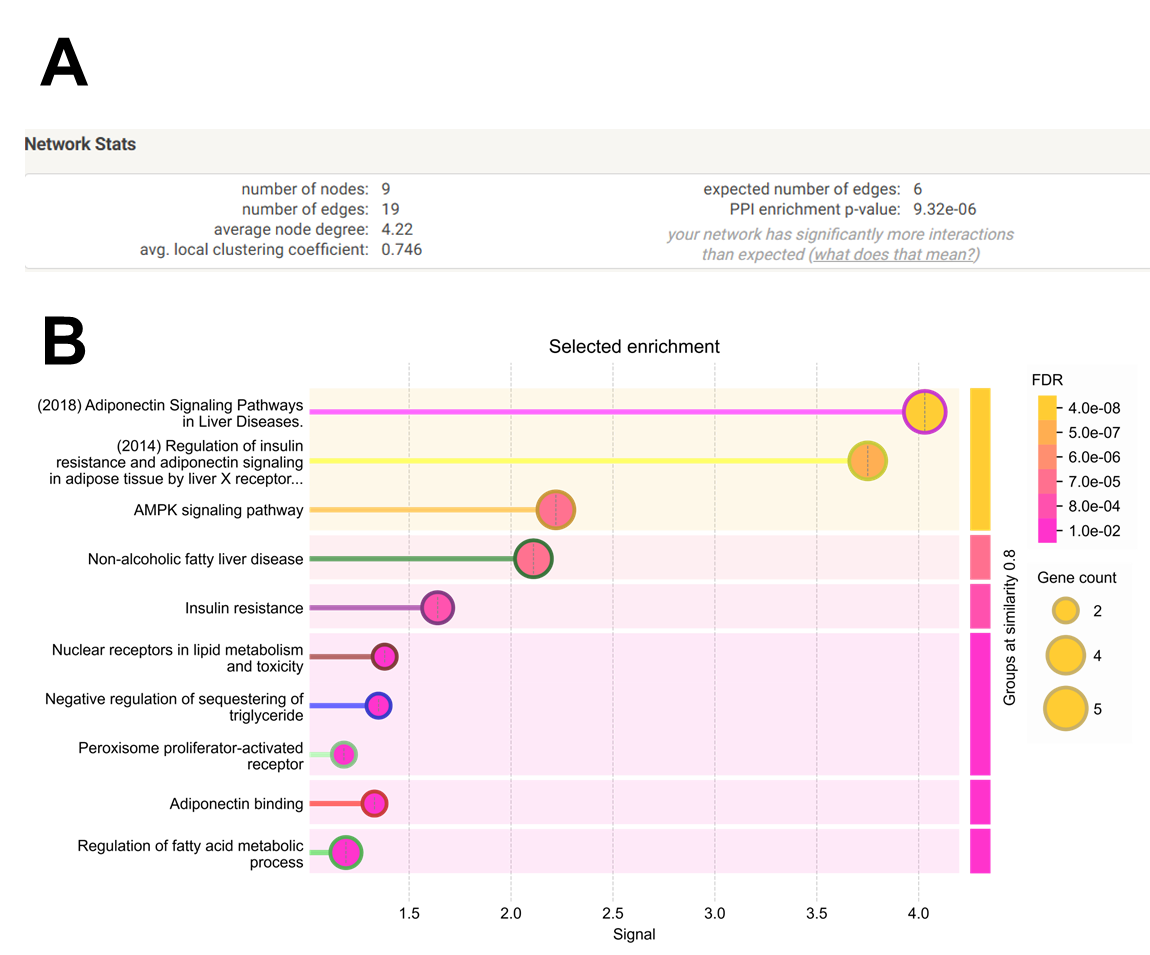
***Figure S10. STRING database analysis of protein-protein interactions based on the differentially expressed lipid metabolism genes. (A)*** *Network statistics obtained from the in-silico analysis;* ***(B)*** *Enrichment functions modulated by the concerned genes.*


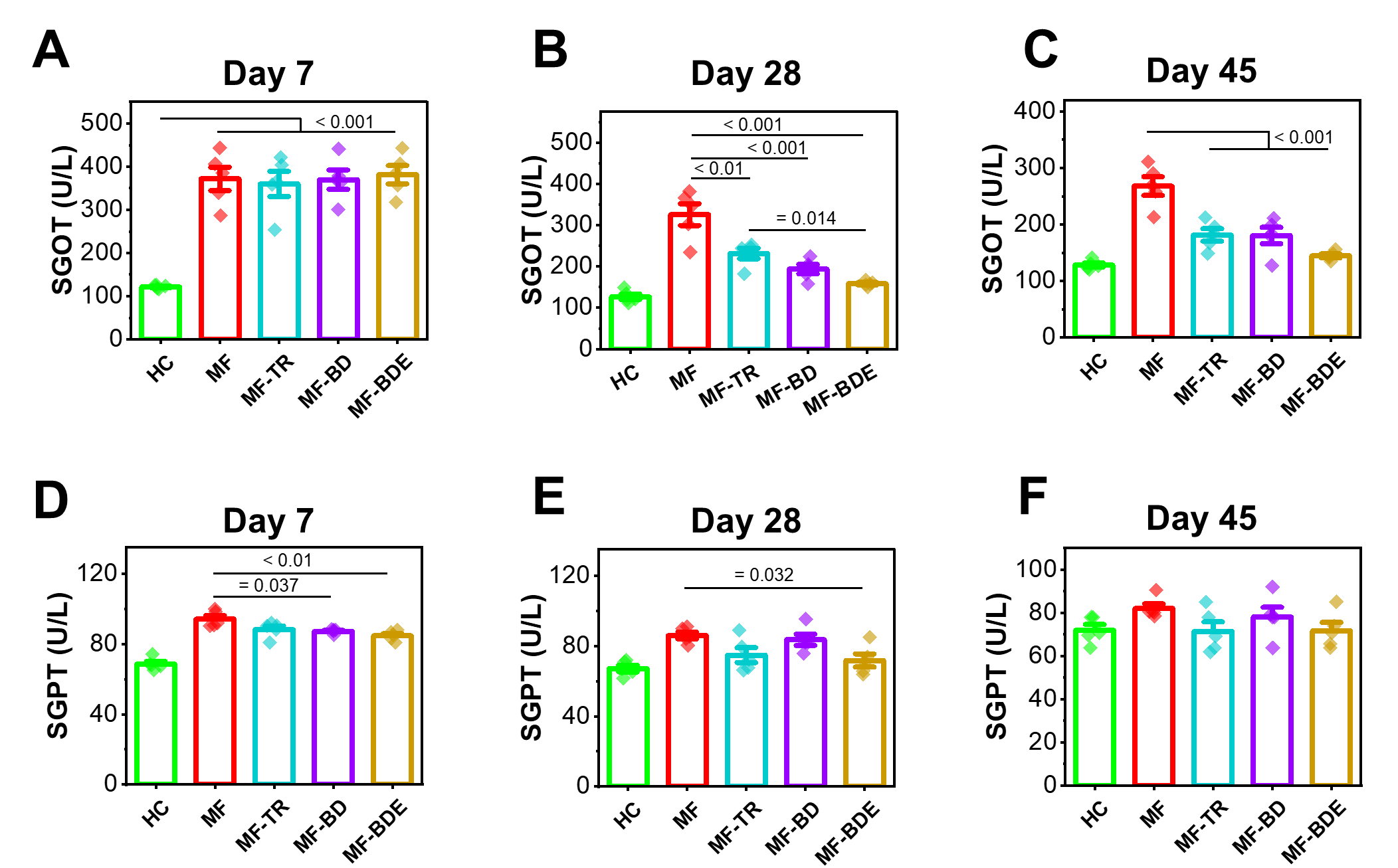
***Figure S11. Evaluation of the liver injury parameters post-therapeutic intervention. (A-C)*** *Serum aspartate transaminase (AST/SGOT) levels on days 7, 28, and 45 of evaluation;* ***(D-F)*** *Serum alanine transaminase (ALT/SGPT) levels on days 7, 28, and 45. Data is expressed as Mean ± S.E., n≥4.*


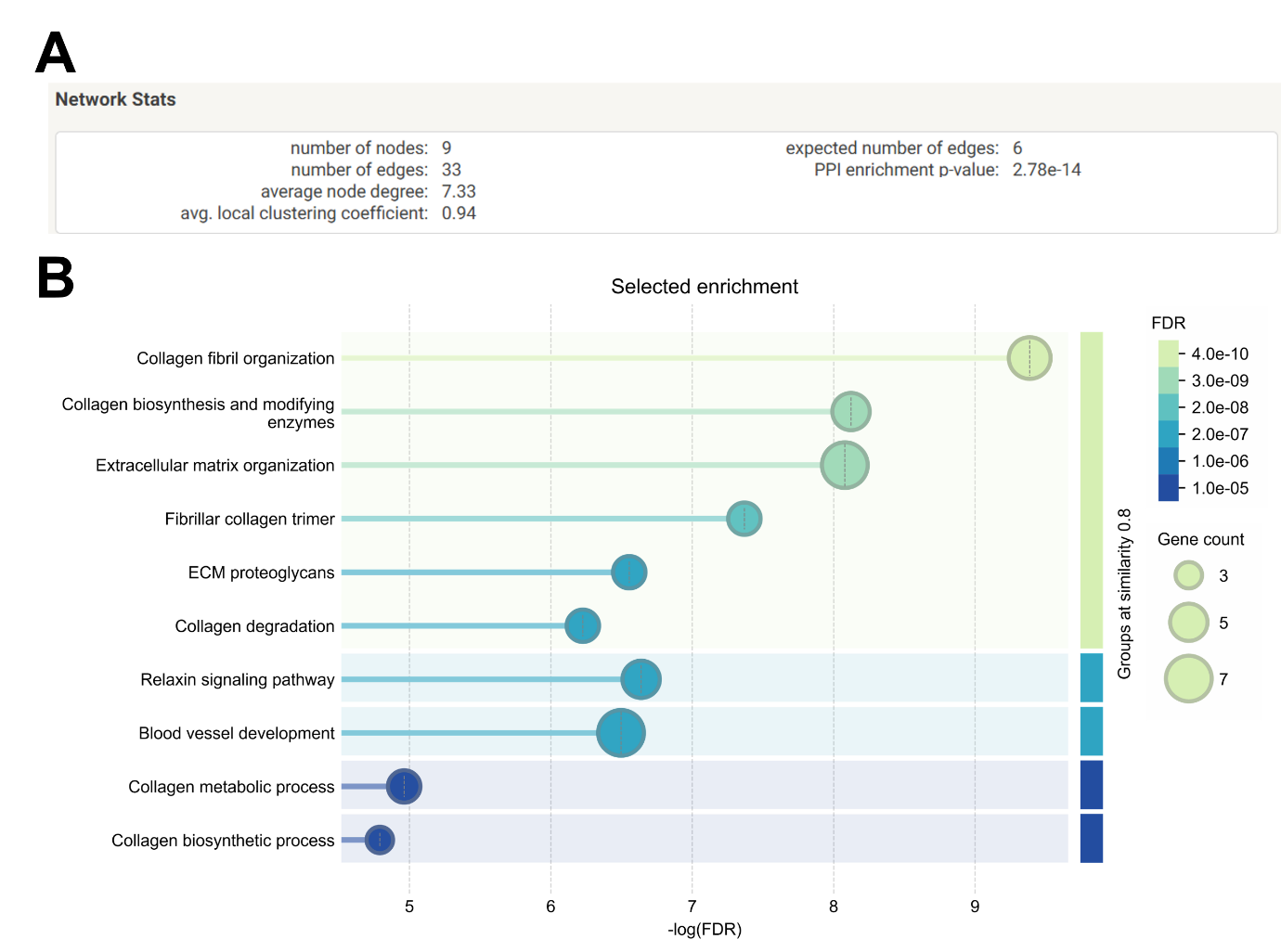
***Figure S12. STRING database analysis of protein-protein interactions and functions mediated by the differentially expressed genes associated with liver fibrosis. (A)*** *Network status depicting the number of nodes, edges, average node degree, and local clustering coefficient;* ***(B)*** *Selected enrichment showing the various functions modulated by the differentially expressed genes.*


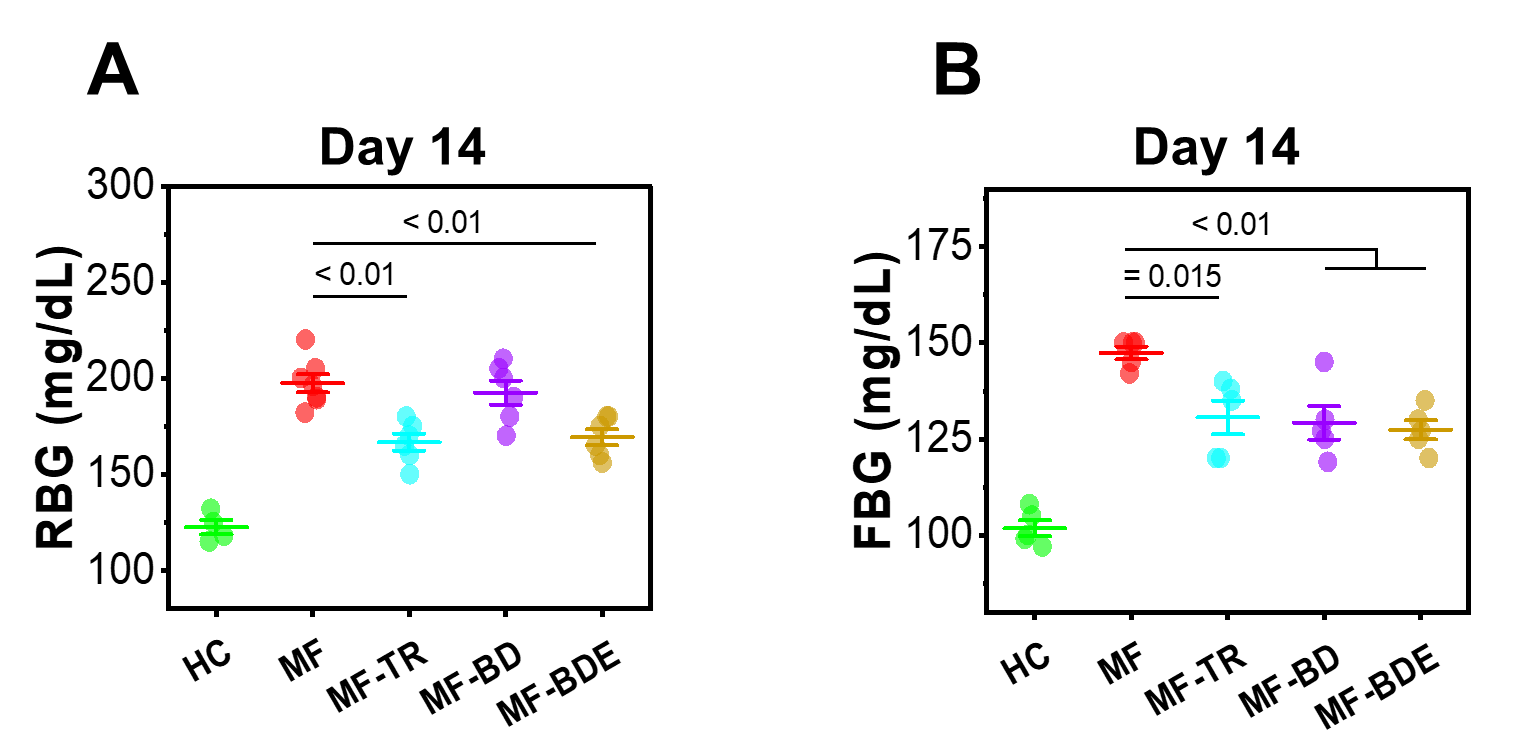


***Figure S13. Glucose metabolism evaluation of the experimental groups. (A)*** *Random blood glucose (RBG) levels on day 14;* ***(B)*** *Fasting blood glucose (FBG) levels on day 14 of assessment. Data is expressed as Mean ± S.E., n≥4.*


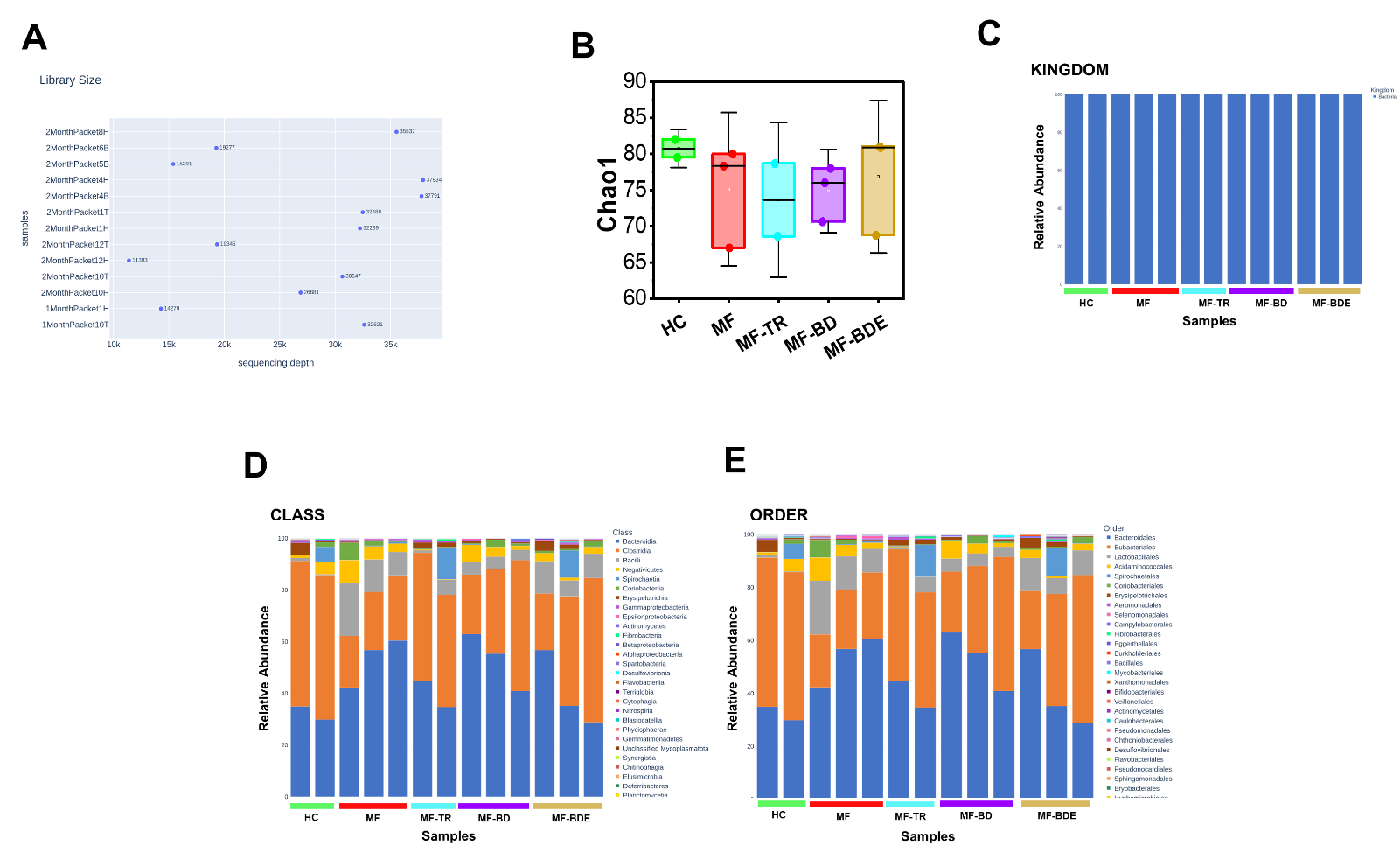
***Figure S14. 16S rRNA sequencing to assess the gut microbiome population. (A)*** *Library sizes of different samples belonging to various experimental groups;* ***(B)*** *Chao1 index of the various animal groups;* ***(C-E)*** *Relative abundance of microbiome populations at Kingdom, Class, and Order levels.*

***Figure S15. KEGG function analysis of the differential microbiome population detected and their associated functions related to metabolism and homeostasis. (A-B)*** *Abundance of the different functions in the healthy group;* ***(C-E)*** *KEGG analysis of the negative/untreated animal group;* ***(F-G)*** *Altered functions abundance in treadmill exercise group;* ***(H-J)*** *Altered abundance of functions in biospray group, and* ***(K-M)*** *In BioNano spray intervened group.*

***Figure S16. In-silico analysis of the differential expressed genes between BioNano spray (MF-BDE) vs Untreated controls (MF) responsible to modulate MAFLD induced altered metabolism. (A-C)*** *KEGG pathway analysis of several downregulated and upregulated genes responsible for lipid metabolism;* ***(D-F)*** *GO pathway analysis showing the downregulated and upregulated genes responsible for functions altering lipid metabolism;* ***(G-I)*** *KEGG pathway analysis of several downregulated and upregulated genes responsible for insulin metabolism;* ***(J-L)*** *GO pathway analysis showing the downregulated and upregulated genes responsible for functions altering insulin metabolism.*

***Figure S17. In-silico analysis of the differential expressed genes between BioNano spray (MF-BDE) vs Untreated controls (MF) responsible to modulate MAFLD induced altered metabolism. (A-C)*** *KEGG pathway analysis of several downregulated and upregulated genes responsible for inflammatory pathways;* ***(D-F)*** *GO pathway analysis showing the downregulated and upregulated genes responsible for functions altering inflammatory pathways;* ***(G-I)*** *KEGG pathway analysis of several downregulated and upregulated genes responsible for glucose metabolism;* ***(J-L)*** *GO pathway analysis showing the downregulated and upregulated genes responsible for functions altering glucose metabolism.*

***Figure S18. In-silico analysis of the differential expressed genes between BioNano spray (MF-BDE) vs Untreated controls (MF) and MF-BDE vs Healthy animal group (HC), responsible to modulate MAFLD-induced systemic alterations. (A-C)*** *KEGG pathway analysis of several downregulated and upregulated genes responsible for fibrosis;* ***(D-F)*** *GO pathway analysis showing the downregulated and upregulated genes responsible for functions altering matrix remodeling and fibrosis;* ***(G-I)*** *KEGG pathway analysis of several downregulated and upregulated genes responsible for lipid metabolism between MF-BDE vs HC;* ***(J-L)*** *GO pathway analysis showing the downregulated and upregulated genes responsible for functions altering lipid metabolism between MF-BDE vs HC.*

***Figure S19. In-silico analysis of the differential expressed genes between healthy animals (HC) vs Untreated controls (MF) responsible to modulate MAFLD induced altered metabolism. (A-C)*** *KEGG pathway analysis of several downregulated and upregulated genes responsible for lipid metabolism;* ***(D-F)*** *GO pathway analysis showing the downregulated and upregulated genes responsible for functions altering lipid metabolism;* ***(G-I)*** *KEGG pathway analysis of several downregulated and upregulated genes responsible for insulin metabolism;* ***(J-L)*** *GO pathway analysis showing the downregulated and upregulated genes responsible for functions altering insulin metabolism.*

***Figure S20. In-silico analysis of the differential expressed genes between healthy animals (HC) vs Untreated controls (MF) responsible to modulate MAFLD induced inflammation and fibrosis. (A-C)*** *KEGG pathway analysis of several downregulated and upregulated genes responsible for inflammation;* ***(D-F)*** *GO pathway analysis showing the downregulated and upregulated genes responsible for functions altering inflammatory pathways;* ***(G-I)*** *KEGG pathway analysis of several downregulated and upregulated genes responsible for glucose metabolism;* ***(J-L)*** *GO pathway analysis showing the downregulated and upregulated genes responsible for functions altering glucose metabolism;* ***(M-O)*** *KEGG pathway analysis of several downregulated and upregulated genes responsible for liver fibrosis;* ***(P-R)*** *GO pathway analysis showing the downregulated and upregulated genes responsible for functions altering liver fibrosis.*

**Table S1:** Detailed composition of cholesterol-based high-fat emulsion and high sucrose diet (HCD-HSD).

| **Components of high-fat emulsion diet (1L)** | **Weight (g)** |
| --- | --- |
| Corn Oil | 200 |
| Cholesterol | 50 |
| Autoclaved distilled water (mL) | 600 |
| Sucrose | 75 |
| Cooking salt | 5 |
| Milk powder | 40 |
| Mineral mixture | 0.75 |
| Vitamin mixture | 1.25 |
| Propylene glycol | 15.6 |
| Sodium deoxycholate | 5 |
| Tween 80 | 18.2 |

**Table S2. Detailed animal groups and the respective number of animals used for various assessments in our study.**

| **Serial No.**  **(Sl No.)** | **Experimental groups** | **No. of**  **Animals** | **DXA Analysis** | **Biochemical Assessment** | **Glucose profile/ Insulin/GTT** | **Histological evaluations** | **Gene expression**  **analysis** |
| --- | --- | --- | --- | --- | --- | --- | --- |
| 01 | Healthy control **(HC)** | 9 | 6 | 5 | 7 | 3 | 4 |
| 02 | Chronic MAFLD **(MF)** | 9 | 6 | 5 | 7 | 3 | 4 |
| 03 | Chronic MAFLD + Treadmill Exercise  **(MF-TR)** | 9 | 6 | 5 | 7 | 3 | 4 |
| 04 | Chronic MAFLD + Biospray  **(MF-BD)** | 9 | 6 | 5 | 7 | 3 | 4 |
| 05 | Chronic MAFLD + BioNano Spray **(MF-BDE)** | 9 | 6 | 5 | 7 | 3 | 4 |

**Table S3. Parameters assessed to quantify the inflammation grade from histological images.**

| **Inflammation Grade** | |
| --- | --- |
| 0 | No inflammatory foci per 200x field |
| 1 | <2 foci or 2-4 foci per 200x field |
| 2 | >4 foci per 200xfield |

**Table S4. Parameters utilized to evaluate the steatosis score.**

| **Steatosis Score** | |
| --- | --- |
| 0 | < 5% steatosis area |
| 1 | 5-33% steatosis area |
| 2 | 33-66% steatosis area |
| 3 | >66% steatosis area |

**Table S5. Details of primary antibodies utilized for immuno histological staining.**

| SL. No. | Name | Manufacturer | Dilution |
| --- | --- | --- | --- |
| 1. | Mouse monoclonal 8-OHdG | Santacruz | 1:200 |
| 2. | Mouse monoclonal α-SMA | Santacruz | 1:50 |
| 3. | Mouse monoclonal collagen type I antibody | Sigma Aldrich | 1:200 |
| 4. | Rabbit monoclonal desmin | Abcam | 1:200 |

**Table S6: List of genes with the forward and reverse primer sequences utilized for gene expression assessments.**

| **Gene** | **Forward primer (5′-3′)** | **Reverse primer (5′-3′)** |
| --- | --- | --- |
| β-actin | 5′-CTAAGGCCAACCGTGAAAAG-3′ | 5′-TACATGGCTGGGGTGTTGA-3′ |
| TNF-α | 5′-TCTGTGCCTCAGCCTCTTCT-3′ | 5′-GGCCATGGAACTGATGAGA-3′ |
| TLR4 | 5′-CAGGATGATGCCTCTCTTGC-3′ | 5′-TGATCCATGCATTGGTAGGTAA-3′ |
| Caspase-1 | 5′-ACCAGGCATATTCTTTCATGTGT-3′ | 5′-TGCTTTCTGCTCTTCAACACC-3′ |
| IL1β | 5′-TCCCACGAGTCACAGAGGA-3′ | 5′-AGCTTCAGGAAGGCAGTGTC-3′ |
| IL18 | 5′-CCTTCCATCCTTCACAGATAGG-3′ | 5′-CCTGATATCGACCGAACAGC-3′ |
| PPARG | 5′-GGGGGTGATATGTTTGAACTTG-3′ | 5′-CAGGAAAGACAACAGACAAATCA-3′ |
| PPARA | 5′-GAGGGGAAGCCCAAATAGA-3′ | 5′-GGGGCAGTGACAGGTAAGG-3′ |
| SREBP-1c | 5′-GCGTGGTGGTCGTCCTAAT-3′ | 5′-TCAGGAGCAGAAGCACTTGAC-3′ |
| Collagen 1A1 | 5′-AAGGGAGGAGAGAGTGCCAA-3′ | 5′-GTCTCTTGCTTCCTCCCACC-3′ |
| Collagen 3A1 | 5′-TGCAATGTGGGACCTGGTTT-3′ | 5′-GGGCAGTCTAGTGGCTCATC-3′ |
| α-SMA | 5′-CATCCGACCTTGCTAACGGA-3′ | 5′-AATAGCCACGCTCAGTCAGG-3′ |
| TGF-β1 | 5′-GCAACAACGCAATCTATGAC-3′ | 5′-CCTGTATTCCGTCTCCTT-3′ |
| ZO-1 | 5′-CCATCTTTGGACCGATTGCTG-3′ | 5′-TAATGCCCGAGCTCCGATG-3′ |
| MUC-2 | 5’- GCCAGATCCCGAAACCA-3’ | 5’- TATAGGAGTCTCGGCAGTCA-3’ |
| IL6 | 5’-AAGGACCAAGACCATCCAAC-3’ | 5’-ACCACAGTGAGGAATGTCCA-3’ |
| CD206 | 5’-TCAACTCTTGGACTCACGGC-3’ | 5’-CATGATCTGCGACTCCGACA-3’ |
| IL10 | 5’-GTTGCCAAGCCTTGTCAGAA-3’ | 5’-GGGAGAAATCGATGACAGCG-3’ |
| Ki67 | 5’-AGGACTTTGTGCTCTGTAACC-3’ | 5’-CTCTTTTGGCTTCCATTTCTTC-3’ |
| CD31 | 5’-CCAGAAAGACAAGGCGATCG-3’ | 5’-CGGCTGGAGGAGAGTTCTAG-3’ |
| iNOS | 5’-CCTTGTTCAGCTACGCCTTC-3’ | 5’-GGTATGCCCGAGTTCTTTCA-3’ |

**Table S7: List of genes with the forward and reverse primer sequences utilized for gene expression assessments for assessing macrophage polarization.**

| **Gene** | **Forward primer (5′–3′)** | **Reverse primer (5′–3′)** |
| --- | --- | --- |
| iNOS | 5’-AATCTCTGCCTATCCGTCTC-3’ | 5’-CAAGCCCTCACCTACTTCCTG-3’ |
| IL-6 | 5’-TAGTCCTTCCTACCCCAATTTCC-3’ | 5’-TTGGTCCTTAGCCACTCCTTC-3’ |
| IL-10 | 5’-GCTCTTACTGACTGGCATGAG-3’ | 5’-CGCAGCTCTAGGAGCATGTG-3’ |
| β-actin | 5’-TACAGCTTCACCACC-3’ | 5’-ATGCCACAGGATTTC-3’ |
| CD163 | 5’-TGTGACCATGCTGAGGATGT -3’ | 5’-CTCGACCAATGGCACTGATG -3’ |

**Table S8: STRING analysis to determine enriched protein-protein interactions associated with lipid metabolism homeostasis.**

**Number of nodes:9**

**Number of edges: 19**

**Average node degree: 4.22**

**Avg. local clustering coefficient: 0.746**

**Expected number of edges: 6**

**PPI enrichment p-value: 9.32e-06**

| **Color** | **Category** | **Term ID** | **Term description** | **Matching proteins in your network (labels)** |
| --- | --- | --- | --- | --- |
| Red | GO Function | GO:0055100 | Adiponectin binding | Adipor1, Adipor2 |
| Lightgreen | InterPro | IPR003074 | Peroxisome proliferator-activated receptor | Pparg, Ppara |
| Orange | KEGG | rno04152 | AMPK signaling pathway | Adipor1, Adipor2,Pparg,Srebf1 |
| Darkgreen | KEGG | rno04932 | Non-alcoholic fatty liver disease | Adipor1,Adipor2,Ppara,Srebf1 |
| Purple | KEGG | rno04931 | Insulin resistance | Ppargc1b,Ppara,Srebf1 |
| Cyan | KEGG | rno03320 | PPAR signaling pathway | Pparg,Ppara |
| Magenta | Publications | PMID:29735928 | (2018) Adiponectin Signaling Pathways in Liver Diseases. | Adipor1,Adipor2,Pparg,Ppara,Srebf1 |
| Yellow | Publications | PMID:24972069 | (2014) Regulation of insulin resistance and adiponectin signaling in adipose tissue by liver X receptor activation highlights a cross-talk with PPARgamma. | Adipor1,Adipor2,Pparg,Ppara |
| Blue | GO Process | GO:0010891 | Negative regulation of sequestering of triglyceride | Pparg,Ppara |
| Limegreen | GO Process | GO:0019217 | Regulation of fatty acid metabolic process | Pparg,Ppara,Srebf1 |
| Lightblue | GO Process | GO:0042593 | Glucose homeostasis | Adipor1,Adipor2,Pparg |

**Table S9: STRING analysis to determine enriched protein-protein interactions associated with liver fibrosis progression.**

**Number of nodes:9**

**Number of edges: 33**

**Average node degree: 7.33**

**Avg. local clustering coefficient: 0.94**

**Expected number of edges: 6**

**PPI enrichment p-value: 2.78e-14**

| **#color** | **category** | **term ID** | **term description** | **matching proteins in your network (labels)** |
| --- | --- | --- | --- | --- |
| maroon | GO Component | GO:0005583 | Fibrillar collagen trimer | Col3a1,Col1a1,Col5a1,Col1a2 |
| yellow | GO Function | GO:0005201 | Extracellular matrix structural constituent | Col3a1,Col1a1,Col5a1,Col1a2 |
| darkgreen | KEGG | rno04926 | Relaxin signaling pathway | Col3a1,Col1a1,Col1a2,Tgfb1,Acta2 |
| red | GO Process | GO:0030199 | Collagen fibril organization | Col3a1,Col1a1,Col5a1,Col1a2,Serpinh1,Loxl2 |
| pink | GO Process | GO:0030198 | Extracellular matrix organization | Col3a1,Col1a1,Col5a1,Col1a2,Serpinh1,Tgfb1,Loxl2 |
| lightgreen | GO Process | GO:0001568 | Blood vessel development | Col3a1,Col1a1,Col5a1,Col1a2,Tgfb1,Acta2,Loxl2 |
| tan | GO Process | GO:0032963 | Collagen metabolic process | Col1a1,Col5a1,Col1a2,Serpinh1 |
| blue | GO Process | GO:0032964 | Collagen biosynthetic process | Col1a1,Col5a1,Serpinh1 |
| limegreen | GO Process | GO:0085029 | Extracellular matrix assembly | Col3a1,Col1a2,Tgfb1 |
| navy | GO Process | GO:0007179 | Transforming growth factor beta receptor signaling pathway | Col3a1,Col1a2,Tgfb1 |
| lightblue | GO Process | GO:0033280 | Response to vitamin D | Serpinh1,Tgfb1 |
| purple | Reactome | RNO-1650814 | Collagen biosynthesis and modifying enzymes | Col3a1,Col1a1,Col5a1,Col1a2,Serpinh1 |
| khaki | Reactome | RNO-3000178 | ECM proteoglycans | Col3a1,Col1a1,Col5a1,Col1a2 |
| orange | Reactome | RNO-1442490 | Collagen degradation | Col3a1,Col1a1,Col5a1,Col1a2 |
| magenta | TISSUES | BTO:0002741 | Hepatic stellate cell | Col1a1,Tgfb1 |

**References**

1. Shiekh, P.A., A. Singh, and A. Kumar, *Exosome laden oxygen releasing antioxidant and antibacterial cryogel wound dressing OxOBand alleviate diabetic and infectious wound healing.* Biomaterials, 2020. **249**: p. 120020.

2. Singh, A., et al., *Transplantation of engineered exosomes derived from bone marrow mesenchymal stromal cells ameliorate diabetic peripheral neuropathy under electrical stimulation.* Bioactive Materials, 2021. **6**(8): p. 2231-2249.

3. Saha, T., et al., *Exosomal miRNA combined with anti-inflammatory hyaluronic acid-based 3D bioprinted hepatic patch promotes metabolic reprogramming in NAFLD-mediated fibrosis.* Biomaterials, 2025. **318**: p. 123140.

4. Mamaghani, K.R., et al., *Synthesis and microstructural characterization of GelMa/PEGDA hybrid hydrogel containing graphene oxide for biomedical purposes.* Materials Today: Proceedings, 2018. **5**(7): p. 15635-15644.

5. Warnick, G.R., et al., *Estimating low-density lipoprotein cholesterol by the Friedewald equation is adequate for classifying patients on the basis of nationally recommended cutpoints.* Clinical chemistry, 1990. **36**(1): p. 15-19.

6. Fukuyama, N., et al., *Validation of the Friedewald equation for evaluation of plasma LDL-cholesterol.* Journal of clinical biochemistry and nutrition, 2007. **43**(1): p. 1-5.

7. Szapary, P.O., et al., *Effects of pioglitazone on lipoproteins, inflammatory markers, and adipokines in nondiabetic patients with metabolic syndrome.* Arteriosclerosis, thrombosis, and vascular biology, 2006. **26**(1): p. 182-188.

8. Mirzaalian, Y., et al., *The association of quantitative insulin sensitivity indices (HOMA-IR and QUICKI) with anthropometric and cardiometabolic indicators in adolescents.* Archives of Medical Science-Atherosclerotic Diseases, 2019. **4**(1): p. 32-37.

9. Dey, S., et al., *Microfluidic Human Physiomimetic Liver Model as a Screening Platform for Drug Induced Liver Injury.* Biomaterials, 2024: p. 122627.

10. Szklarczyk, D., et al., *STRING v10: protein–protein interaction networks, integrated over the tree of life.* Nucleic acids research, 2015. **43**(D1): p. D447-D452.
